# Part 2. Mechanistic aspects of the reduction of *S*-alkyl-thionocarbonates in the presence of triethylborane and air

**DOI:** 10.1186/1860-5397-3-46

**Published:** 2007-12-12

**Authors:** Florent Allais, Jean Boivin, Van Tai Nguyen

**Affiliations:** 1 Institut de Chimie des Substances Naturelles, C.N.R.S., Avenue de la Terrasse, 91198 Gif-sur-Yvette, France; 2 Institut National de la Recherche Agronomique, Centre de Versailles, Route de St-Cyr, 78026 Versailles, France; 3 National Institute of Medicine Materials, 3B, Quang Trung, Hanoi, Vietnam

## Abstract

Experiments conducted with deuterated compounds demonstrated that during the reduction of *S*-alkylxanthates with triethylborane, the hydrogen atom transferred has several competing origins. Hydrogen abstraction from water (or an alcohol) is the most favourable route.

## Background

In the first part of this series,[1] we showed that trialkylboranes and especially commercial solutions of Et_3_B are efficient reducing agents that permit the conversion, at room temperature, of *S*-alkylxanthates, iodides and *O*-alkylxanthates into the corresponding alkanes with good to excellent yields. Such a process complies with the long-standing pursuit of an environmentally acceptable process for desulfurisation, dehalogenation or deoxygenation that operates under mild reaction conditions. In this context, special attention must be paid to a paper published by Jaszberenyi and Barton in 1990.[2] The authors described a reduction process of *O*-alkylxanthate and related compounds with Bu_3_SnH/Et_3_B/air at room temperature. They mentioned very briefly that when Bu_3_SnH was omitted, the reduction still occurred. The *O*-cyclododecyl *S*-methylxanthate derived from cyclododecanol afforded cyclododecane in a remarkable 62% yield. No hypothesis about the origin of the hydrogen atom that replaced the original xanthate function was proposed. Recently, as the work reported here was largely completed as already mentioned in the first part of this series,[3–4] Wood and coworkers also observed an "anomalous" reduction of a closely related tertiary *O*-alkylxanthate into the corresponding alkane instead of an expected rearrangement.[5] In a subsequent report on the reduction of *B*-alkylcatecholboranes, Renaud and coworkers showed that the O-H bond in methanol may also be the source of hydrogen. However, the deuterium incorporations in experiments aimed at elucidating the mechanism are relatively low,[6] and obviously the experimental results do not support the hypothesis that the O-H group is the sole source of the hydrogen transferred.

## Results and discussion

In this note, we wish to report our findings concerning the intriguing question of the origin of the hydrogen atom that replaces the radicophilic group in the reduction of *S*-alkyl-thionocarbonates. Several hypotheses may reasonably be proposed. The first possibility is a hydrogen transfer from the solvent. However, dichloromethane, 1,2-dichloroethane and hexane (from the commercial solution of Et_3_B) utilised in the preceding article,[3] are considered to be poor hydrogen atom donors, especially at low temperature.[7] There are only rare reports concerning the ability of an alkane (cyclohexane) to cleanly transfer a hydrogen atom to a specific type of carbon radical.[8–9] Disproportionation between the ethyl radical and the carbon-centred radical derived from the xanthate would have probably given the corresponding olefins, especially in the case of a tertiary radical. Such olefins were never found. A more seemly hypothesis is a hydrogen abstraction from the α-position of the boron atom. This reaction is well documented and energetically acceptable (BDE (C-H) = 80 ± 3 kcal/mol). For example, the methyl radical abstracts α-boronyl hydrogen of Et_3_B 124 times faster than hydrogen in the methyl group of toluene.[10] We also envisioned the possible intermediacy of a transient organoborane species that would undergo protonolysis during work-up. However, a "simple" trialkylborane intermediate is not a plausible route since the attack of a carbon radical on the boron atom is not favourable.[10]

**Figure 1:**
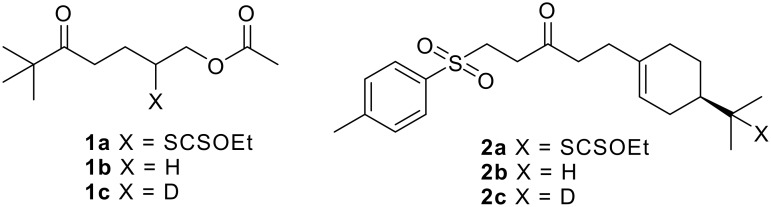
Xanthates **1a**, **2a** and their corresponding alkanes.

In an attempt to gain information about the mechanism, we performed deuteration experiments (Figure 1 and Table 1). Most of the results reported herein concern the reduction of xanthate **1a** [see Supporting Information File 1, Supporting Information File 2, and Supporting Information File 3]. This substrate was chosen because of its low molecular weight and its simple structure that ensure easy spectral analyses (NMR, MS, GC-MS) and also because a putative 1,5-hydrogen shift between the intermediate radical and a hydrogen atom in the α-position to the ketone cannot intervene. This point was discussed at the end of the first part of this series.[3] On the other hand, we proved that a similar 1,5-hydrogen shift in which the acetyl group would be implicated does not occur either (see below). The standard experiment (entry 1) performed without any source of deuterium serves to evaluate the natural abundance of ^13^C in compound **1c** and to calibrate the deuterium measurements.

**Table 1:** Deuteration experiments

Entry	Xanthate	Products^e^	Solvent	D %

1	**1a**	**1b**	Et_2_O	0
2	**1a**	**1b + 1c**	CDCl_3_/CD_3_OD ^a^	88
3	**1a**	**1b + 1c**	(CH_2_Cl)_2_/CD_3_OD ^a^	85
4	**1a**	**1b + 1c**	(CH_2_Cl)_2_/CH_3_OD ^a^	76
5	**1a**	**1b + 1c**	(CH_2_Cl)_2_/D_2_O ^a^	83
6	**1a**	**1b + 1c**	THF/D_2_O ^a^	83
7	**1a**	**1b + 1c**	THF 3 h, then D_2_O ^a^	<1
8	**1a**	**1b + 1c**	3 h then CH_3_OD ^a^	6
9	**1a**	**1b + 1c**	20 min then CH_3_OD ^a^	17
10	**1a**	**1b + 1c**	THF; D_2_O/H_2_O = 25 equiv/25 equiv	6
11	**1a**	**1b + 1c**	H_2_O/D_2_O = 5 equiv/100 equiv^b^	66
12	**1a**	**1b + 1c**	H_2_O/D_2_O = 20 equiv/80 equiv^b^	32
13	**2a**	**2b + 2c**	(CH_2_Cl)_2_/CH_3_OD^a^	93^f^
14	**1a**	**1b + 1c**	CDCl_3_^c^	57^g^
15	**1a**	**1b + 1c**	CDCl_3_^c^/D_2_O^d^	83
16	**1a**	**1b + 1c**	C_6_D_6_^c^	<1
17	**1a**	**1b + 1c**	C_6_D_6_^c^/D_2_O^d^	70
18	**1a**	**1b + 1c**	CDCl_3_^c^/H_2_O^d^	3
19	**1a**	**1b + 1c**	THF-*d*_8_	<1^h^

Reduction of *S*-alkylxanthates (0.3–0.4 mmol) with Et_3_B (5 equiv from a commercial solution in hexanes)/dry air for 3 h, unless otherwise stated. Xanthates were reduced according to method A except in experiment 13 where method B was used. ^a^ 50 equiv of deuterated methanol or water were used. ^b^ No other organic solvent than hexanes from the commercial Et_3_B solution. ^c^ Reactions performed with freshly prepared 1M solutions of pure Et_3_B in CDCl_3_ or C_6_D_6_. ^d^ 5 equiv of D_2_O or H_2_O. ^e^ All the yields in pure **1b + 1c** are superior to 61% except for experiment 16 (yield 40%). ^f^ Yield **2b** + **2c** = 70%. ^g^ When the reaction was performed with CDCl_3_ dried over K_2_CO_3_, the deuterium incorporation fell to 15.8%. ^h^ This experiment was carried out twice.

A possible source of hydrogen could be an alcohol present as a contaminant in various solvents, therefore we chose to examine the effect of addition of methanol to the reaction mixture. When xanthate **1a** was treated with Et_3_B/dry air, according to method A,[3] in a mixture of chloroform-*d* and methanol-*d*_4_, the incorporation of deuterium was determined to be 88% (entry 2). The analysis of the spectral data (^1^H, ^2^H and ^13^C NMR and mass) unambiguously shows that the incorporated deuterium is located at the same position as the parent xanthate. This proves that no 1,5-hydrogen shift occurred. The replacement of chloroform-*d* by 1,2-dichloroethane gave similar results (85%, entry 3). More interestingly, the incorporation was still high when methanol-*d*_4_ was replaced by CH_3_OD (76%, entry 4). Methanol-*d*_4_ or CH_3_OD can be in turn replaced by D_2_O without significant change in the level of deuterium incorporation (entries 5 and 6), even in a solvent known to be a good hydrogen donor such as THF (entry 6). Experiments 2–6 established that, in the presence of deuterated methanol or water, the incorporated deuterium may come from rupture of an O-D bond. The results given in entries 7 and 8 show that there is no noticeable incorporation of deuterium when D_2_O or CH_3_OD are added after 3 h, a period after which all the starting material **1a** has been consumed. On the other hand, experiment 9, where CH_3_OD was added after 20 min before consumption of all the starting material, indicates that the deuterated compound is formed *pro-rata* with time of contact with CH_3_OD (compare entries 9 and 4). Under the same conditions, we observed that there is no more incorporation of deuterium when CH_3_OD was added after 30 min (results not shown). Therefore, there is no accumulation of an intermediate species responsible for the conversion of the xanthate group into the alkane. Experiments 10, 11 and 12 prove that the incorporation of deuterium does not parallel the ratio H_2_O/D_2_O. Even small amounts of H_2_O led to a significant decrease of deuterium incorporation. This may be explained as the consequence of a significant isotopic effect or of an alternative mechanism in which the O-H(D) bond is not implicated. We showed that the incorporation of deuterium was uppermost in the case of tertiary xanthate **2a** (entry13).

Eventually, we performed several experiments using solutions of pure Et_3_B in deuterated solvents, with or without added H_2_O or D_2_O (entries 14–19). When chloroform-*d* was used as solvent, the percentage of deuterium incorporation is still high (57%, entry 14). By contrast, when the reaction is performed in benzene-*d*_6_, no deuterium is incorporated (entry 16). These two experiments indicate that, depending on its ability to transfer hydrogen, the solvent may also be a source of hydrogen. Finally, experiments 6, 15 and 17 show that when D_2_O is present the incorporation of deuterium is very high and thus transfer of D from D_2_O surpasses that from the other sources. Experiment 18 corroborates this conclusion: deuterium incorporation from CDCl_3_ is suppressed when H_2_O is present. Noteworthy is the observation that when the reaction is performed in THF-*d*_8_, no deuterium incorporation occurs (entry 19).

The above experiments shed light on the role of water or alcohols and solvents in the reduction of *S*-alkylxanthates and related compounds. Hydrogen transfer from the O-H bond present in water or in an alcohol is not an obvious hypothesis in radical chemistry because of the high BDE of the O-H bond. Our results corroborate Wood's findings concerning reduction of *O*-alkylxanthates.[5] This author also came to the conclusion that water is the source of hydrogen and proposed two routes. The first one invokes an innovative concept in which water, complexed to a trialkylborane, is the hydrogen atom donor. Gaussian 3G calculations indicate that such a complexation would lower the BDE of the O-H bond by 30 kcal/mol when compared to free H_2_O, thus rendering hydrogen abstraction from water a plausible process. The second route implies hydrogen abstraction from •O-O-H radical (O-H BDE 47.6 kcal/mol). The latter could possibly be formed by interaction of dialkylboron peroxy radical R_2_BOO• with water and should depend on the amount of dioxygen. It is worth noting that both routes necessitate at least stoechiometric amounts of water. Wood and his colleagues[5] serendipitously observed this anomalous reduction of *O*-alkylxanthates, working on very small scales (0.006 mmol). Conspicuously, in further experiments aimed at elucidating the origin of the hydrogen atom, the authors deliberately added high quantities (5 to 20 equiv) of H_2_O or D_2_O.

This mechanism is different from the process involved in the case of α-acyl xanthates where an intermediate enol boronate is a likely intermediate that can be easily protonolysed during work-up. When xanthate **1a** was reduced with Et_3_B/air in methanol-d_4_, a mixture of compound **1b** and its mono-deuterated analogue (55% deuterium incorporation, determined by ^1^H NMR) was obtained. However, because of a possible deuterium-proton exchange through enolisation, this observation cannot be used to ascertain the mechanism.

The results reported in Table 1 prove that competitive radical mechanisms are operative: when water (or an alcohol) is present, the abstraction of the hydrogen atom obviously takes place by breaking an O-H bond, whereas in the absence of water, this hydrogen transfer could happen by hydrogen abstraction from the solvent (provided that it is a reasonably good hydrogen atom donor). However, despite all our efforts to operate under strictly anhydrous conditions, we observed good yield of hydrogen incorporation in experiments carried out in completely deuterated solvents (Table 1, entries 14 and 16). This suggests that no water is needed and that a third source of hydrogen atom, different from water and solvent, might be involved.

**Figure 2:**
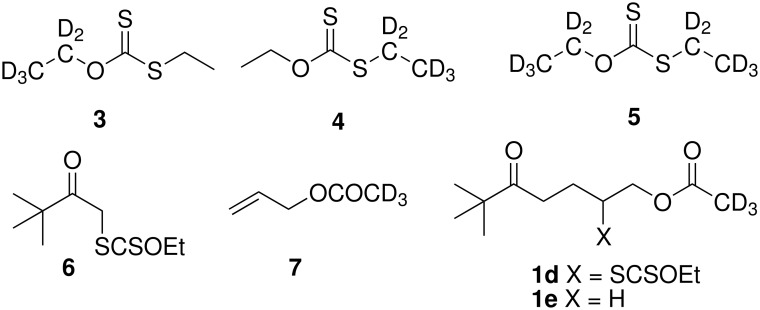
Deuterated *O*-ethyl-*S*-ethyl dithiocarbonates **3–5**, deuterated adduct **1d** and alkane **1e**.

We first focused our attention on *O*-ethyl-*S*-ethyl dithiocarbonate as a possible source of hydrogen atom. This by-product is produced by reaction of ethyl radical, generated from Et_3_B, with the xanthate function. Deuterated compounds **3–5** were easily prepared from ethanol, carbon disulfide and ethyl bromide (Figure 2). The results reported in Table 2 clearly show that no deuterium transfer is observed from either compounds **3**, **4**, or **5** when xanthate **1a** was subjected to reduction (Method A) in the presence of one equivalent of *O*-ethyl-*S*-ethyl dithiocarbonate, using benzene as solvent to suppress any hydrogen atom transfer from the solvent.

**Table 2:** Reduction of xanthate **1a** in the presence of deuterated *O*-ethyl *S*-ethyl dithiocarbonates **3–5** in C_6_H_6_.

Entry^a^	Xanthate	Additive (1 equiv)	Et_3_B (eq)^b^	Yield % **1b** + **1c**	Yield % **1a**	D%

1	**1a**	**3**	5	40	55	<1
2	**1a**	**4**	5	36	58	<1
3	**1a**	**5**	5	37	58	<1

^a^ Reactions performed in Teflon "glassware" using 0.312 mmol of xanthate **1a**. ^b^ Freshly prepared 1 M solutions of pure Et_3_B in C_6_H_6_ were used.

Along the same lines, we prepared compound **1d** by addition of xanthate **6** to deuterated allyl acetate **7** (Figure 2). The reaction of xanthate **1d** with Et_3_B (5 equiv) in benzene (Method A) afforded the corresponding reduced compound **1e**. Examination of NMR and mass spectra of compounds **1d** and **1e** shows that during both the addition and the reduction processes no deuterium loss or scrambling occurred. In particular, a putative 1,5-hydrogen transfer between the transient radical and the CD_3_ group is clearly ruled out.

**Figure 3:**
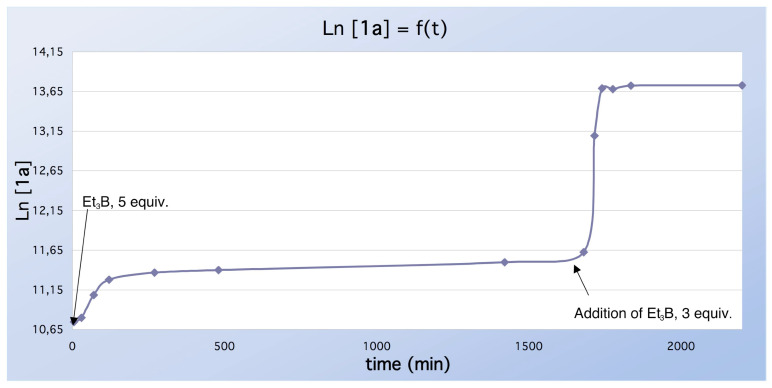
Kinetics of the reduction of compound **1a** in C_6_H_6_ at 20°C.

Incidentally, we noticed that when the reduction of xanthate **1d** (or **1a**) with 5 equiv of Et_3_B was performed in benzene, the reaction was not complete even after 18 h, and much starting material was recovered (see Table 2). We therefore performed a kinetic monitoring of the reaction using a GC technique. The reduction was carried out according to Method A (0.2 M solution of xanthate **1a** in a freshly prepared solution of 1 M Et_3_B in benzene, *i.e.* 5 equiv). The experimental graph (Figure 3) shows that after 100 min the formation of the reduced compound **1b** slows down rapidly to reach a plateau. Upon addition of a further three equivalents of Et_3_B (1 M solution in benzene, t = 1700 min), the reduction started again and rapidly attained another plateau. Plotting of Ln [**1a**] *vs* time indicates that, when BEt_3_ is used in excess (0 < t < 120 min), the reduction follows a pseudo-first order kinetic equation relative to the concentration [**1a**]. Linear regression calculations gave Ln [**1a**] = 10.7 + 4.9 10^-3^ t with a correlation factor of 0.988. GCMS analysis at 1800 min showed that all the starting material was consumed. The yield of compound **1b** then reached 82% (isolated yield). At this time, we cannot explain why the reduction is more sluggish in benzene than in other solvents. Noteworthy, Wood and colleagues [5] used a huge amount of Et_3_B (20–50 equiv) when performing the reaction in benzene.

**Scheme 1:**
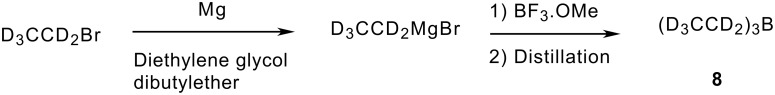
Preparation of Et_3_B-*d*_15_.

As put forth above, a hydrogen abstraction from Et_3_B is an energetically credible hypothesis. To verify this, we undertook the synthesis of Et_3_B-*d*_15_. After testing an improvement of a procedure published by H.C. Brown and co-workers in which Et_3_B is prepared from ethyl bromide, we succeeded in obtaining Et_3_B-*d*_15_ **8** in high purity from commercially available ethyl bromide-*d*_5_ (Scheme 1).[11] The selectively deuterated compounds (CH_3_CD_2_)_3_B **9** and (CD_3_CH_2_)_3_B **10** were prepared in a similar fashion from CH_3_CD_2_Br and CD_3_CH_2_Br, respectively [see Supporting Information File 4].

When xanthate **1a** was reacted with perdeuterated triethylborane (5 equiv), an 8.3% incorporation of deuterium was observed in the resulting reduced compounds **1b** + **1c** (Table 3, entry 1). This result is perfectly reproducible as shown by comparison of entries 1 and 2. When the reaction was performed in a dried Teflon round bottom flask, the incorporation increased to 26.7%. This result is also quite reproducible (entry 3 *vs* entry 4). We have shown above that no deuterium transfer was seen from dithiocarbonate **4**, a by-product formed during the reduction of compound **1a** in the presence of deuterated triethylborane. Therefore, one must assume that the deuterium transfer occurs *directly* from Et_3_B-*d*_15_ to the transient carbon radical formed by fragmentation of the xanthate function in compound **1a**. Interestingly, the deuterium abstraction occurs not only from the methylene group as anticipated, but also from the methyl group (entries 5 and 6). However, the abstraction from the methylene group, after statistical correction, is approximately 5.5 times more efficient than from the methyl group. Examination of Table 3 also strongly suggests that the surface of the glassware might play a role in hydrogen atom donation: substituting merely glass by Teflon brings about an increase of the deuterium incorporation by a factor of three. Conversely, when the reaction was carried out in the same Teflon flask under the same conditions as in experiments 3 and 4, but in the presence of small glass rings, the incorporation of deuterium dropped back to 8.3% (entry 7). Complexation between the O-H groups on the surface of the silica and triethylborane, analogous to that postulated between water and triethylborane, appears to be a consistent hypothesis to explain the hydrogen transfer. However, this hypothesis seems difficult to test directly as the preparation of deuterated glassware is not a straightforward process: the replacement of hydrogen by deuterium on the surface of silica requires very harsh conditions.[12–13]

**Table 3:** Reduction of xanthate 1a with Et_3_B-*d*_15_, Et_3_B-*d*_6_ and Et_3_B-*d*_9_ in C_6_H_6_.

Entry^b^	Xanthate **1a** (mmol)	Deuterated Et_3_B	**1b + 1c** Yield %	**1a** (recovered) Yield %	D %

1	0.300	Et_3_B-*d*_15_ **8**	38	52	8.3
2	0.300	Et_3_B-*d*_15_ **8**	39	60	8.3
3	0.300^a^	Et_3_B-*d*_15_ **8**	37	61	26.7
4	0.300^a^	Et_3_B-*d*_15_ **8**	38	55	25.9
5	0.300^a^	Et_3_B-*d*_6_ **9**^d^	43	48	12.9
6	0.300^a^	Et_3_B-*d*_9_ **10**^d^	40	52	3.5
7^c^	0.300^a^	Et_3_B-*d*_15_ **8**	35	57	8.3

^a^ The reaction was performed in a dry Teflon 25 mL round bottom flask.^b^ Experiments 1–2 were carried out using the same 25 mL round bottom flask. ^c^ Same reaction conditions as in experiments 3 and 4, except that small glass rings (1 g) were introduced into the flask. All the reactions were performed using 5 equiv of triethylborane. ^d^ (CH_3_CD_2_)_3_B **9** and (CD_3_CH_2_)_3_B **10** were prepared from commercially available CH_3_CD_2_Br and CD_3_CH_2_Br respectively, according to the same procedure as the one described for the synthesis of perdeuterated triethylborane.

## Conclusion

Undeniably, the reduction of *S*-alkyl-thionocarbonates conceals several subtleties. In our quest for the origin of the transferred hydrogen, we demonstrated by unambiguous deuteration experiments that three types of sources are implicated. The first one relies on hydrogen donation from an O-H group (presumably complexed to the trialkylborane). This O-H group may belong to water, an alcohol, and, in all likelihood, to the surface of the glassware. We showed that, when present, water or an alcohol (methanol) is the preferred hydrogen donor. The second involves a hydrogen transfer from the solvent, as far as it constitutes an acceptable hydrogen donor. The last source is triethylborane itself. The hydrogen abstraction occurs at the α position to boron and also, to a minor extent, at the β position. It is important to note that the reduction gave good yields even under anhydrous conditions (or without alcohol). This allows extensions of this method to water-sensitive compounds to be envisioned. This will be the subject of a forthcoming paper. In fact, the qualitative results reported in this article indicate that the overall reduction process seems to be the result of a delicate balance between three different elementary mechanisms.

Very recently, Newcomb,[14] following Wood's and Renaud's reports, carried out kinetic estimations of the rate of hydrogen abstraction by an alkyl radical from the complex between Et_3_B and water using the routine radical clock method. This author also came to the conclusion that hydrogen abstraction from the α position of Et_3_B must be invoked. No spectral or chemical evidence was furnished either to ascertain the formation of a complex between Et_3_B and water or to demonstrate that hydrogen abstraction from triethylborane is effective. However, it is very gratifying for us to note that this author found the same order of magnitude for the rate of the hydrogen transfer as we estimated in this paper and in the preceding part of this series.[3]

Despite a lack of experimental proof at this time, one may postulate that the reduction of *O*-alkylxanthates and iodides with triethylborane/air also embodies the same plurality of mechanisms.[3] The reactivity of the combination alkylborane/air/water touches many other domains and certainly deserves further study.

## Supporting Information

File 1 Procedure for the preparation of deuterated triethylboranes, Procedures for the reduction with deuterated triethylboranes. Preparation of deuterated Et_3_B (*d*-15, *d*-9, and *d*-6).

File 2 Part 2. Mechanistic aspects of the reduction of *S*-alkyl-thionocarbonates in the presence of triethylborane and air. Detailed procedures for preparation of new compounds and their spectroscopic data, pp 1–10.

File 3 Part 2. Mechanistic aspects of the reduction of *S*-alkyl-thionocarbonates in the presence of triethylborane and air. Detailed procedures for preparation of new compounds and their spectroscopic data, pp 11–43.

File 4 Part 2. Mechanistic aspects of the reduction of *S*-alkyl-thionocarbonates in the presence of triethylborane and air. Detailed procedures for preparation of new compounds and their spectroscopic data, pp 44–56.
