# Supplementary material for: Part 2. Mechanistic aspects of the reduction of S-alkyl-thionocarbonates in the presence of triethylborane and air
Source: Beilstein J Org Chem. 2007 Dec 12;3:46. doi: 10.1186/1860-5397-3-46 (PMC2213662; doi:10.1186/1860-5397-3-46)
Supplement: File 1 — Procedure for the preparation of deuterated triethylboranes, Procedures for the reduction with deuterated triethylboranes. Preparation of deuterated Et3B (d-15, d-9, and d-6). [file Beilstein_J_Org_Chem-03-46-s001.pdf]

Part 2. Mechanistic aspects of the reduction of *S*-alkyl-thionocarbonates in the presence of triethylborane and air

Florent Allais,[1,2] Jean Boivin,*[1] and Van Tai Nguyen*[1,3]

Addresses: [1]Institut de Chimie des Substances Naturelles, C.N.R.S., Avenue de la Terrasse, 91198 Gif-sur-Yvette, France, [2]Present address: Institut National de la Recherche Agronomique, Centre de Versailles, Route de St-Cyr, 78026 Versailles, France, [3]Present address: National Institute of Medicine Materials, 3B, Quang Trung, Hanoi, Vietnam,

Email: Florent Allais – florent.allais@versailles.inra.fr; Jean Boivin*- jean.boivin@icsn.cnrs-gif.fr; Van Tai Nguyen* - htqt.imm@hn.vnn.vn

Procedure for the preparation of deuterated triethylborane

Deuterated triethylborane **8**: To a flame-dried 3-necked round-bottom flask, equipped with an addition funnel and a Vigreux distillation apparatus, were added successively magnesium turnings (3.71 g, 152.80 matg), freshly distilled boron trifluoride dimethyletherate (3.48 g, 2.81 mL, 30.56 mmol), a crystal of iodine and anhydrous diethyleneglycol dibutyl ether 20 mL, b.p. 256 °C). The reaction was initiated by addition of pure ethylbromide-d_5 (few drops) while vigourously stirring the reaction mixture under argon atmosphere. The remaining ethylbromide-d_5 (10.45 g, 91.68 mmol) was dissolved in anhydrous diethyleneglycol dibutyl ether (15 mL) and was slowly added over a period of 2 h, giving rise to a noticeable warming. The reaction mixture was stirred at 50 °C for an additional period of 2 h. The distillation was achieved under argon directly from the reaction mixture, at atmospheric pressure, and afforded pure Et_3B-d_{15} (1.90 g, 55 %). B.p. 93-97 °C. Pure Et_3B-d_{15} 8 was used to prepare 1 M solutions in anhydrous benzene and deuterated benzene. These solutions were prepared under inert atmosphere (glove bag) and stored in Sure-Pac cylinders.

^{2}H NMR (76.8 MHz, C_6D_6): δ=6.25 (s, 6D), 6.02 (s, 9D). ^{13}C NMR (125 MHz, C_6D_6): δ=19.94-17.64 (m, CD_2), 7.34 (hept, J=18.3 Hz). ^{11}B (160.5 MHz, C_6D_6): δ=87.47 (s, $W_{1/2}$=82 Hz, internal standard: $BF_3.Et_2O$ in a sealed capillary).

Procedures for the reduction with deuterated triethylboranes

Methods A and B have been described in Part 1 of this series.
