# Supplementary material for: Part 2. Mechanistic aspects of the reduction of S-alkyl-thionocarbonates in the presence of triethylborane and air
Source: Beilstein J Org Chem. 2007 Dec 12;3:46. doi: 10.1186/1860-5397-3-46 (PMC2213662; doi:10.1186/1860-5397-3-46)
Supplement: File 2 — Part 2. Mechanistic aspects of the reduction of S-alkyl-thionocarbonates in the presence of triethylborane and air. Detailed procedures for preparation of new compounds and their spectroscopic data, pp 1–10. [file Beilstein_J_Org_Chem-03-46-s002.pdf]

# Supporting Information Available

# Part 2. Mechanistic Aspects of the Reduction of *S*-Alkyl-thionocarbonates in the Presence of Triethylborane and Air

*Florent Allais,*[a] *Jean Boivin,**[a] *and Van Tai Nguyen**[a,b]

a) Institut de Chimie des Substances Naturelles, C.N.R.S.,

Gif-sur-Yvette 91198, France.

b) National Institute of Medicinal Materials

jean.boivin@icsn.cnrs-gif.fr *and* htqt.imm@hn.vnn.vn

# Table of Contents


| | |
|---|---|
| **General Experimental Methods** | **Page S3** |
| **Reagents and Starting Materials** | **Page S3** |
| **Deuterium measurements:** | **Page S4** |
| **Deuterated compounds (1c) and (2c)** | **Page S5** |
| **Synthesis of O,S-diethyl dithiocarbonates (3-5)** | **Page S6** |
| **Allyl acetate-d3 (7)** | **Page S8** |
| **2-[(Ethoxycarbonothioyl)thio]-6,6-dimethyl-5-oxoheptyl acetate-$d_3$** | **Page S8** |
| **6,6-Dimethyl-5-oxoheptyl acetate-$d_3$** | **Page S8** |
| **Synthesis of triethylborane and deuterated triethylboranes (8), (9), and (10)** | **Page S9** |
| **References** | **Page S10** |
| **Spectral Data** | **Page S11** |
| **Deuterium incorporation measurements** | **Page S54** |

**General Experimental Methods:**

Solvents and reagents were purified according to standard literature techniques[1] and stored under argon. Experiments that required an inert atmosphere were carried out under dry argon in a flame dried glass system. Column chromatography was carried out on silica gel (230-400 mesh) by gradual elution with mixtures of n-heptane and ethyl acetate. TLC was carried out on using $F_{254}$ $SiO_2$ coated aluminum plates (0,2 μm, analytical). Vizualisation was accomplished with UV light (254 nm), typical TLC indicating solution (*p*-anisaldehyde/sulfuric acid/ethanol mixture). $^1H$ and $^{13}C$ NMR spectra were recorded in deuteriochloroform on 300, 400 or 500 MHz spectrometers. $^2H$ NMR spectra were recorded in chloroform on a 600 MHz spectrometer. The chemical shifts are reported δ unit, parts per million (ppm) relative to deuterated solvents ($^1H$, δ 7.269 ppm; $^{13}C$, δ 77.23 ppm) or $Me_4Si$. Splitting pattern of an apparent multiplet associated with an averaged coupling constant were designated as s (singlet), d (doublet), t (triplet), q (quartet), m (multiplet), dd (doublet of doublet), dt (doublet of triplet), dq (doublet of quartet) and br (broadened). Coupling constants (*J* value) were reported in Hz unit. Deuterium incorporations were determined by analysis of isotope patterns obtained from electrospray ionization (ESI) or chemical ionization (CSI) with 2-methylpropane spectra.

**Reagents and Starting Materials:**

Triethylborane (1M solution in hexanes), tri-*n*-butylborane (1M in THF), and $CH_3OD$ (99% isotopic purity) were purchased from commercial suppliers, used without further purification, and stored under nitrogen atmosphere. $D_2O$ (99.90% isotopic purity) and $CD_3OD$ (99.80% isotopic purity) were used without further purification. All other commercially reagents were used as received.

1M solution of $Et_3B$ in $CDCl_3$ and 1M solution of $Et_3B$ in $C_6D_6$ were prepared under argon atmosphere (glove bag, Ar atmosphere) by dissolving commercial $Et_3B$ (caution! pyrophoric) in $CDCl_3$ and $C_6D_6$ respectively.

Compounds **1a**, **1b**, **2a**, **2b** and **6** are known substances.[2]

**Deuterium measurements:**

Measurements were made on a LCT Micromass , UK equipped with an electrospray source (Z-source) in positive mode. The external reference (Gramicidine S, MH+ 571.3608) was introduced in parallel with the sample in a continuous manner (lockspray configuration). The resolution of the apparatus is 6500 and results are given with a maximum deviation of 5mDa. The sample is solubilised in methanol and injected via an HPLC equipped with an automatic sampler Alliance 2795 Waters. The TDC Stop is 180 mV. The TDC parameter is a threshold value that determines if the signal received by the TDC must be recorded as an ion. If this value is high, the noise is diminished but sensitivity is lower and distortion in the isotopic pattern may occur. In the experiments reported in the paper (Table 2), this threshold value corresponds to 10 % of the higher peak. This "filter" is applied as a default parameter before recording of the data file. Therefore, the M+ Na + 1 peak in the blank (undeuterated) experiment 1 (Table 2) is only 1-2 %: this corresponds to the normal isotopic ($^{13}C$) abundance (~ 12 %) - 10 %. A simulation of the percentage calculations shows that the maximum deviation is < 8 % of the real value that would be measured on an "unfiltered" spectrum for peaks M + Na / M + Na + 1 of intensities 110/12 (or 12/112 by symmetry). No M + Na + 1 peak can be inferior to 12 % of the M + Na peak for a molecule containing 11 Carbon atoms. The deviation is null of course for peaks of equal intensities. We therefore consider that the estimation of the deuterium content is only slightly affected by the intrinsic limitation of the mass spectroscopic technique we used.

Experiments reported in Tables 2 and 3 and in Figure 1 were analysed as follows: Deuterium incorporation percentages were determined by GCMS analysis of the reduced xanthates (**1b**, **1c**). Equipment and method: the GCMS apparatus was equipped with a simultaneous double injection system with a FID detection line and a mass line. Column: DB-5ms of 30 m length. Carrier gas: He flow 24.9 mL.min-1; pressure 134.8 kPa. Injection temperature: 250 °C. Temperature program: $T_{init}$ = 120 °C, hold for 1 min, then 8 °C/min ramp until T = 250 °C. Chemical ionization was achieved with 2-methylpropane. Crude reaction mixtures were concentrated in vacuo and dissolved in methanol to get ca. 1 g.L$^{-1}$ samples. Retention times for **1b** and **1c** were 5.43 min. Mass spectra and relative abundance of fragments were obtained thanks to the qualitative analysis command. This allowed us to measure and calculate the deuterium incorporation percentages by looking at peaks specified in the Note.

**Deuterated compounds 1c and 2c:**

**Compound 1c**

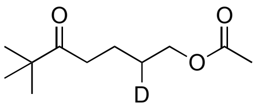

Experiment 3 (see Table 2) is taken as an example. The same procedure was used for experiments 2, 4-18 (except experiment 13).

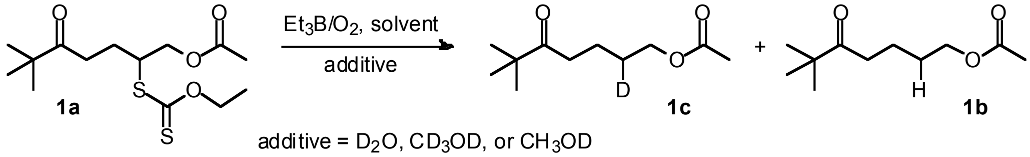


The title compound **1c** was prepared following the general procedure for the radical reductions (method A), starting from xanthate **1a** (0.127 g, 0.40 mmol, dried by several azeotropic evaporations with dry toluene), and $Et_3B$ (1 M solution in hexanes, 1.98 mL, 1.98 mmol, 5 eq.) in 1,2-dichloroethane (1.5 mL) and $CD_3OD$ (0.5 mL). Silica gel column chromatography (eluent heptane/EtOAc = 9/1) gave a yellow oil (0.049 g, 61%). Deuterium incorporation 85%.

$^1$H NMR (500 MHz, $CDCl_3$): δ=4.06 (t, $^3J$=5.3 Hz, 2H), 2.51 (t, $^3J$=6.8 Hz, 2H), 2.04 (s, 3H), 1.61 (m, 3.24H), 1.13 (s, 9H); $^2$H NMR (600 MHz, $CHCl_3$): δ=1,61; $^{13}$C NMR (75 MHz, $CDCl_3$): δ=275.4, 171.1, 64.1, 44.0, 35.7, 27.7 (t, $^3J$=19.4 Hz, C-D), 26.4, 21.0, 20.1; IR (neat): ν =2962, 1739, 1700, 1363, 1237, 1150 cm$^{-1}$; HRMS (ESI): $[M+Na]^+$ Calcd for $C_{11}H_{19}D\,O_3Na$: 224.1373. Found 224.1382.

**Compound 2c**

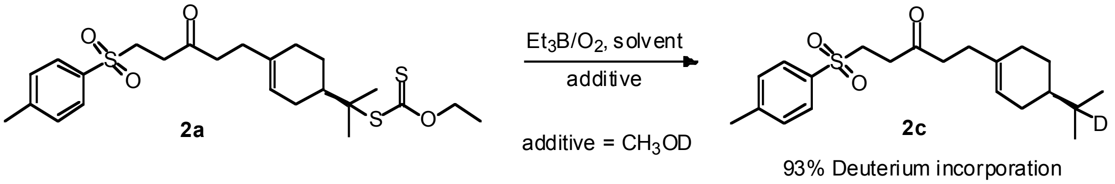

The title compound **2c** was prepared following the general procedure for radical reductions (method B). $Et_3B$ (1 M solution in hexanes, 1.04 mL, 1.04 mmol, 5 eq.) and $CH_3OD$ (0.5 mL). Xanthate **2a** (0.1 g, 0.21 mmol) dissolved in $(CH_2Cl)_2$ (2.0 mL). Silica gel column chromatography (eluent heptane/EtOAc = 9/1) gave a white solid (0.053 g, 70%). Deuterium incorporation 93%.

m.p. 89.9-91.0°C; $^1$H NMR (250 MHz, $CDCl_3$): δ=7.78 (d, $^3J$=8.3 Hz, 2H), 7.36 (d, $^3J$=8.0 Hz, 2H), 5.4 (brs, 1H), 3.35 (t, $^3J$=7.6 Hz, 2H), 2.89 (t, $^3J$=7.6 Hz, 2H), 2.52 (distorted triplet, $^3J$=1.0 Hz, $^3J$=8.3 Hz, 1H), 2.45 (s, 3H), 2.18 (t, $^3J$ =7.5 Hz, 2H), 2.04-1.83 (m, 3H), 1.79-1.65 (m, 2H), 1.29-1.12 (m, 2H), 0.87 (s, 3H), 0.86 (s, 3H); $^2$H NMR (600 MHz, $CHCl_3$): δ=1,43; $^{13}$C NMR (75 MHz, $CDCl_3$): δ=206.0, 144.9, 136.1, 135.6, 130.0, 128.0, 121.6, 50.6, 41.2, 39.9, 35.0, 31.6 (t, J=19.2 Hz, C-D), 31.3, 29.0, 28.8, 26.2, 21.6, 19.8, 19.5; IR (neat): ν =2915, 1712, 1311, 1283, 1144, 1085, 819, 656, 626 $cm^{-1}$; HRMS (ESI): $[M+Na]^+$ Calcd. for $C_{21}H_{29}DO_3NaS$: 386.1876 Found: 386.1859.

**Synthesis of *O*,*S*-diethyl dithiocarbonates**

**Synthesis of sodium ethyldithiocarbonate**

To a solution of NaOH (2.14 g, 53.58 mmol) in absolute ethanol (200 mL) at rt was added carbon disulfide (4.0 g, 3.16 mL, 52.53 mmol). The mixture was allowed to stir overnight at rt. The solvent was then evaporated and the white residue wad dried under vacuum to afford sodium ethyldithiocarbonate as a yellow solid (6.8 g, 90%).[3] Known compound.

**Synthesis of deuterated sodium ethyl-*S*-thiocarbonate**

To a solution of NaOH (0.78 g, 19.58 mmol) in deuterated ethanol (1 g, 1 mL, 19.19 mmol) at rt was added carbon disulfide (2.92 g, 2.31 mL, 38.38 mmol). The mixture was allowed to stir overnight at rt. The solvent was then evaporated and the white residue was dried under vacuum to afford deuterated sodium ethyldithiocarbonate as a yellow solid (2.14 g, 75%).

***O*,*S*-diethyl dithiocarbonate-$d_5$ (3)**

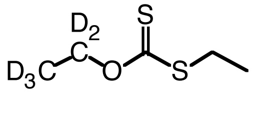


A slurry containing deuterated sodium ethyldithiocarbonate (0.44 g, 2.95 mmol) and ethylbromide (0.32 g, 0.28 mL, 2.95 mmol) in acetone (1.5 mL) was stirred at rt for 15 hours. The sodium

bromide was removed by filtration and washed with acetone, the filtrate was concentrated *in vacuo*. The resulting crude yellow oil was purified by flash chromatography (Heptane/AcOEt 8:2) to afford compound **3** as a yellow liquid (0.450 g, 90%).

$^{1}$H NMR (500 MHz, $CDCl_3$): δ=3.01 (q, $^{3}J$=7.5 Hz, 2H), 1.23 (t, $^{3}J$=7.5 Hz, 2H); $^{13}$C NMR (75 MHz, $CDCl_3$): δ=215.3, 69.2, 68.8, 68.5, 68.2, 68.0, 29.9, 14.2, 13.9, 13.3, 12.9, 12.6, 12.3, 12.1, 11.8; IR (neat) υ=2927, 2243, 1229, 1070, 1034 cm$^{-1}$.

***O,S*-diethyl dithiocarbonate-$d_5$ (4)**

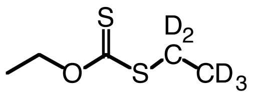


A stirred slurry containing sodium ethyldithiocarbonate (1.40 g, 9.71 mmol) and deuterated ethylbromide (1.11 g, 0.72 mL, 9.71 mmol) in acetone (4.5 mL) was stirred at rt for 15 hours. The sodium bromide was removed by filtration and washed with acetone, the filtrate was concentrated *in vacuo*. The resulting crude yellow oil was purified by flash chromatography (Heptane/AcOEt 8:2) to afford compound **4** as a yellow liquid (1.2 g, 80%).

$^{1}$H NMR (500 MHz, $CDCl_3$): δ=4.63 (q, $^{3}J$=6.9 Hz, 2H), 1.40 (t, $^{3}J$=7.2 Hz, 2H); $^{13}$C NMR (75 MHz, $CDCl_3$): δ=214.1, 69.1, 29.6, 29.2, 29.0, 28.7, 28.3, 14.4, 12.8, 12.6, 12.3, 12.1, 11.8, 11.6; IR (neat) υ=2982, 2360, 2226, 1205, 1050 cm$^{-1}$.

***O,S*-diethyl dithiocarbonate-$d_{10}$ (5)**

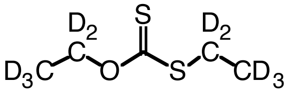


A stirred slurry containing deuterated sodium ethyldithiocarbonate (630 mg, 4.22 mmol) and deuterated ethylbromide (481 mg, 0.32 mL, 4.22 mmol) in acetone (2.1 mL) was stirred at rt for 15 hours. The sodium bromide was removed by filtration and washed with acetone, the filtrate was concentrated *in vacuo*. The resulting crude yellow oil was purified by flash chromatography (Heptane/AcOEt 8:2) to afford compound **5** as a yellow liquid (641 mg, 95%). $^{13}$C NMR (75 MHz, $CDCl_3$): δ=215.1, 69.5, 69.2, 68.9, 68.6, 68.3, 30.1, 29.8, 29.4, 29.1, 28.8, 13.4, 13.1, 12.9, 12.8, 12.6, 12.5, 12.4, 12.2; IR (neat) υ=2925, 2228, 1231, 1077, 1041, 1006 cm$^{-1}$.

**Allyl acetate-$d_3$ (7)**

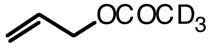

To a flame-dried round bottom flask under argon wad added allylic alcohol (0.30 g, 0.35 mL, 5.16 mmol), pyridine (5 mL) and DMAP (0.32 g, 2.58 mmol) at rt. Deuterated acetic anhydride (0.56 g, 0.49 mL, 5.16 mmol) was then added and the solution was stirred at rt for 18 hours. The mixture was then quenched with a saturated aqueous $NH_4Cl$ solution. The mixture was extracted with diethylether and the combined organic layers were dried over sodium sulfate then filtrated. After concentration *in vacuo*, the crude oil (0.52 g, 98%) was used in the next step without further purification.

$^1$H NMR (500 MHz, $CDCl_3$): δ=5.91 (ddt, $^3J$=5.7, 11.4 and 17.1 Hz, 1H), 5.33-5.19 (m, 2H), 4.55 (dt, $^3J$=1.2 and 5.7 Hz, 2H); $^{13}$C NMR (75 MHz, $CDCl_3$): δ=134.2, 118.4, 65.3; IR (neat) υ=2944, 2360, 1737, 1373, 1225, 1028 cm$^{-1}$.

**2-[(Ethoxycarbonothioyl)thio]-6,6-dimethyl-5-oxoheptyl acetate-*d*$_3$ (1d)**

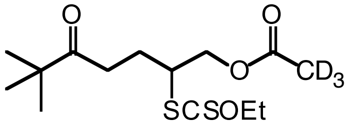

$^1$H NMR (500 MHz, $CDCl_3$): δ=4.53 (q, $^3J$=7.0 Hz, 2H), 4.19 (ABX syst., A part, $J_{AB}$=11.5 Hz, $J_{AX}$=5.0 Hz, 1H), 4.11 (ABX syst., B part, $J_{AB}$=11.5 Hz, $J_{BX}$=6.5 Hz, 1H), 3.83 (ABX syst., X part, $J_{AX}$=5.0 Hz, $J_{BX}$ =6.5 Hz, 1H), 2.23 (t, $^3J$=7.6 Hz, 2H), 2.00 (m, 1H), 1.69 (m, 1H), 1.31 (t, $^3J$=7.0 Hz, 3H), 1.02 (s, 9H). $^{13}$C NMR (75 MHz, $CDCl_3$): δ=214.7, 213.0, 170.8, 70.3, 65.7, 49.0, 44.2, 33.6, 29.3, 26.4, 13.7; IR (neat) υ=2918, 2358, 1740, 1704, 1215, 1047 cm$^{-1}$.

**6,6-Dimethyl-5-oxoheptyl acetate-*d*$_3$ (1e)**

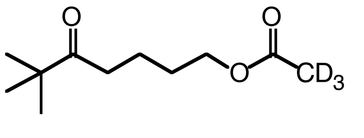

$^1$H NMR (500 MHz, $CDCl_3$): δ=4.03 (t, $^3J$= 5.7 Hz, 2H), 2.49 (t, $^3J$= 6.7 Hz, 2H), 1.59 (m, 4H), 1.11 (s, 9H). $^{13}$C NMR (75 MHz, $CDCl_3$): δ=215.7, 174.4, 64.4, 44.3, 36.0, 28.3, 26.6, 20.5; IR (neat) υ= 2958, 2359, 1735, 1705, 1254 cm$^{-1}$. HRMS (ESI): $[M+Na]^+$ Calcd. for $C_{11}H_{17}D_3NaO_3$: 226.1514 Found: 226.1511.

**MS analysis of compounds (1d) and (1e):**

GCMS analysis of compound **1d** displays peaks at *m/z* 324 ($MH^+$), 261 ($MH^+$-AcOH-$d_3$), and 141 ($MH^+$-AcOH-$d_3$ - $EtOCS_2$). The relative isotope distributions for these peaks are [324 (100), 325 (26), 326 (13)], [261 (100), 262 (16), 263 (11)], and [141 (100), 142 (10)], respectively. These distributions perfectly fit with the calculated values made on the basis of a natural abundance 1.10 % for $^{13}C$ and 4.21 % for $^{34}S$. Therefore, peaks at m/z 324, 261, and 141 correspond respectively to [$C_{14}H_{21}D_3O_4S_2$+H], [$C_{12}H_{20}O_2S_2$+H], and [$C_9H_{16}O$+H] formulas.[4] This demonstrates that the three deuterium atoms in xanthate **1d** are exclusively localized on the acetyl group. This is also the case for the reduced compound **1e** derived from **1d**. The GCMS shows peaks at *m/z* 204/205 ($MH^+$, relative intensities 100/12) and 141/142 ($MH^+$-AcOH-$d_3$, relative intensities 100/10). The same reasoning as above permits to assert that during both the addition and the reduction processes no deuterium loss or scrambling occur.

**Synthesis of triethylborane and deuterated triethylborane:**

**<u>Caution!</u> Triethylborane is highly pyrophoric.[5] The preparation and all the operations were conducted under inert atmosphere (Argon) using syringes and canula.**

**Triethylborane:** To a flame-dried 3-necked round-bottom flask, equipped with an addition funnel and a Vigreux distillation apparatus, were added successively magnesium turnings (3.71 g, 152.80 matg), freshly distilled boron trifluoride dimethyletherate (3.48 g, 2.80 mL, 30.56 mmol), a crystal of iodine and anhydrous diethyleneglycol dibutyl ether (60 mL). The reaction was initiated by addition of pure ethylbromide (few drops) while vigorously stirring the reaction mixture under argon atmosphere. The remaining ethylbromide (9.99 g, 91.68 mmol) was dissolved in anhydrous diethyleneglycol dibutyl ether (30 mL) and was slowly added over a period of 2 h, giving rise to a noticeable warming. The reaction mixture was stirred at 50 °C for an additional period of 2 h. The distillation was achieved under argon directly from the reaction mixture, at atmospheric pressure and afforded pure $BEt_3$ (1.25 g, 42 %). B.p. 93-97 °C (lit. 95 °C).[6] The above $Et_3B$ was used to

prepare 1 M solutions in $CDCl_3$, benzene and deuterated benzene. These solutions were prepared under inert atmosphere (glove bag) and stored in Sure-Pac cylinders.

**Perdeuterated triethylborane $Et_3B$-$d_{15}$ (8):** see the Experimental Section in the Note.

**Deuterated triethylborane $Et_3B$-$d_6$ (9):** see preparation of compound **8**. Starting from ethylbromide-$d_2$ (5 g), yield 73 %. $^1$H NMR (500 MHz, $CDCl_3$): δ=0.92. (s). $^2$H NMR (76.8 MHz, $C_6D_6$): δ=6.25 (s).

**Deuterated triethylborane $Et_3B$-$d_9$ (10):** see preparation of compound **8**. Starting from ethylbromide-$d_3$ (5 g), yield 70 %. $^1$H NMR (500 MHz, $CDCl_3$): δ=1.11 (s). $^2$H NMR (76.8 MHz, $C_6D_6$): δ=6.07 (s).

---

**References:**

[1] Perrin, D. D.; Armarego, W. L. F.; Perrin, D.R., *Purification of Laboratory Chemicals*, Pergamon Press, 1980.

[2] Boivin, J.; Nguyen, V. T. *J. Org. Chem.,* **2007**, submitted.

[3] Hlasiwetz, H. *Justus Liebigs Ann. Chem.* **1862**, *122*, 88.

[4] 2-Methylpropane was used for chemical ionization.

[5] The combination triethylborane/oxygen was used on military aircrafts SR-71 "Blackbird" to light up the Pratt & Whitney J58 engines and to light up the afterburner in flight. 600 mL of triethylborane were sufficient for at least 16 starts, restarts, or afterburner lights. See also: http://www.nasa.gov/centers/dryden/pdf/88598main_H-2280.pdf.

[6] Brown, H. ; Racheria, U. *J. Org. Chem.* **1986**, *51*, 427-432.
