# Supplementary material for: Part 2. Mechanistic aspects of the reduction of S-alkyl-thionocarbonates in the presence of triethylborane and air
Source: Beilstein J Org Chem. 2007 Dec 12;3:46. doi: 10.1186/1860-5397-3-46 (PMC2213662; doi:10.1186/1860-5397-3-46)
Supplement: File 3 — Part 2. Mechanistic aspects of the reduction of S-alkyl-thionocarbonates in the presence of triethylborane and air. Detailed procedures for preparation of new compounds and their spectroscopic data, pp 11–43. [file Beilstein_J_Org_Chem-03-46-s003.pdf]

t235f5.001
21/09/05

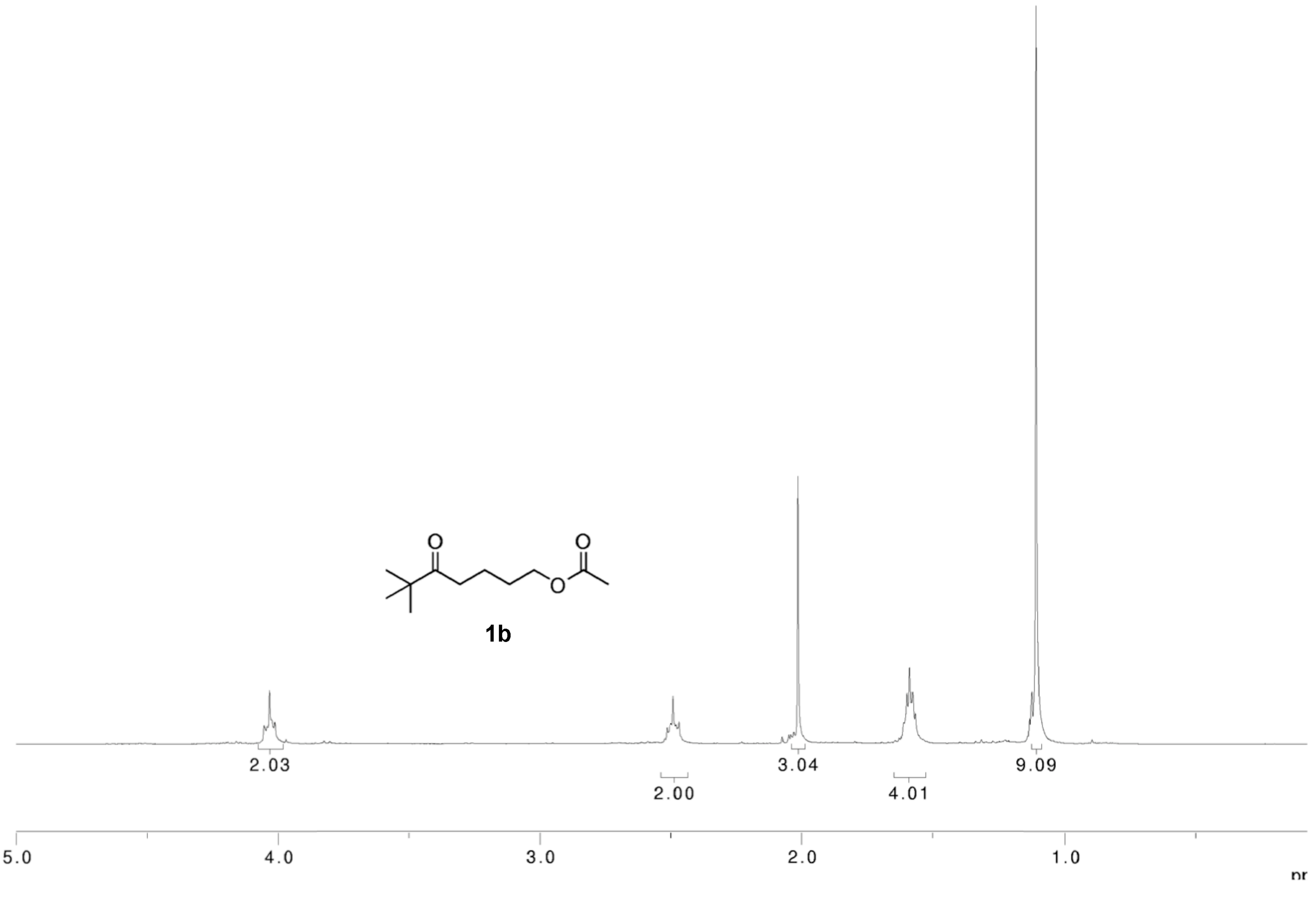

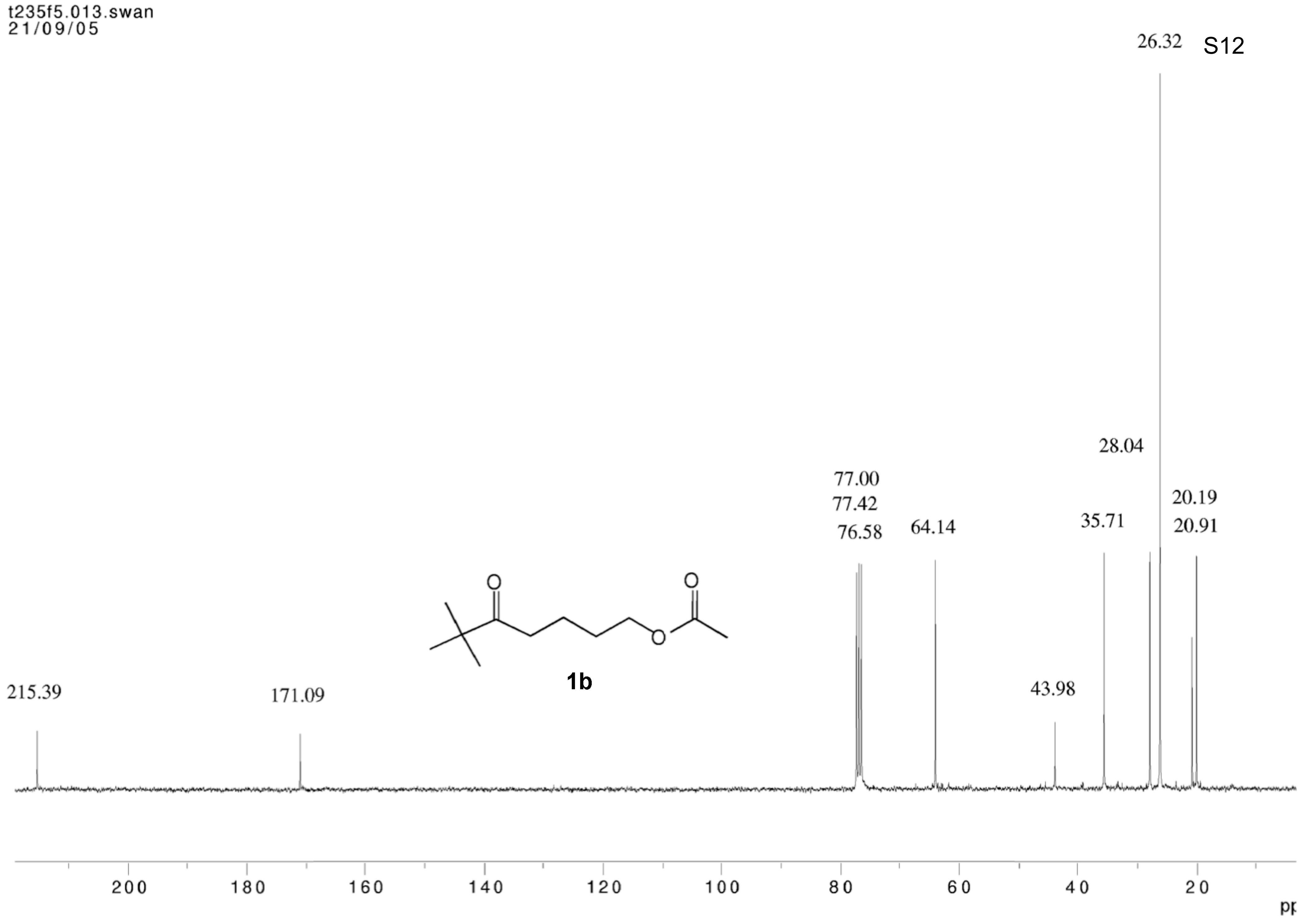
t235f5.013.swan
21/09/05
26.32
28.04
77.00
77.42
76.58
64.14
35.71
20.19
20.91
215.39
171.09
43.98
1b
200
180
160
140
120
100
80
60
40
20
pp

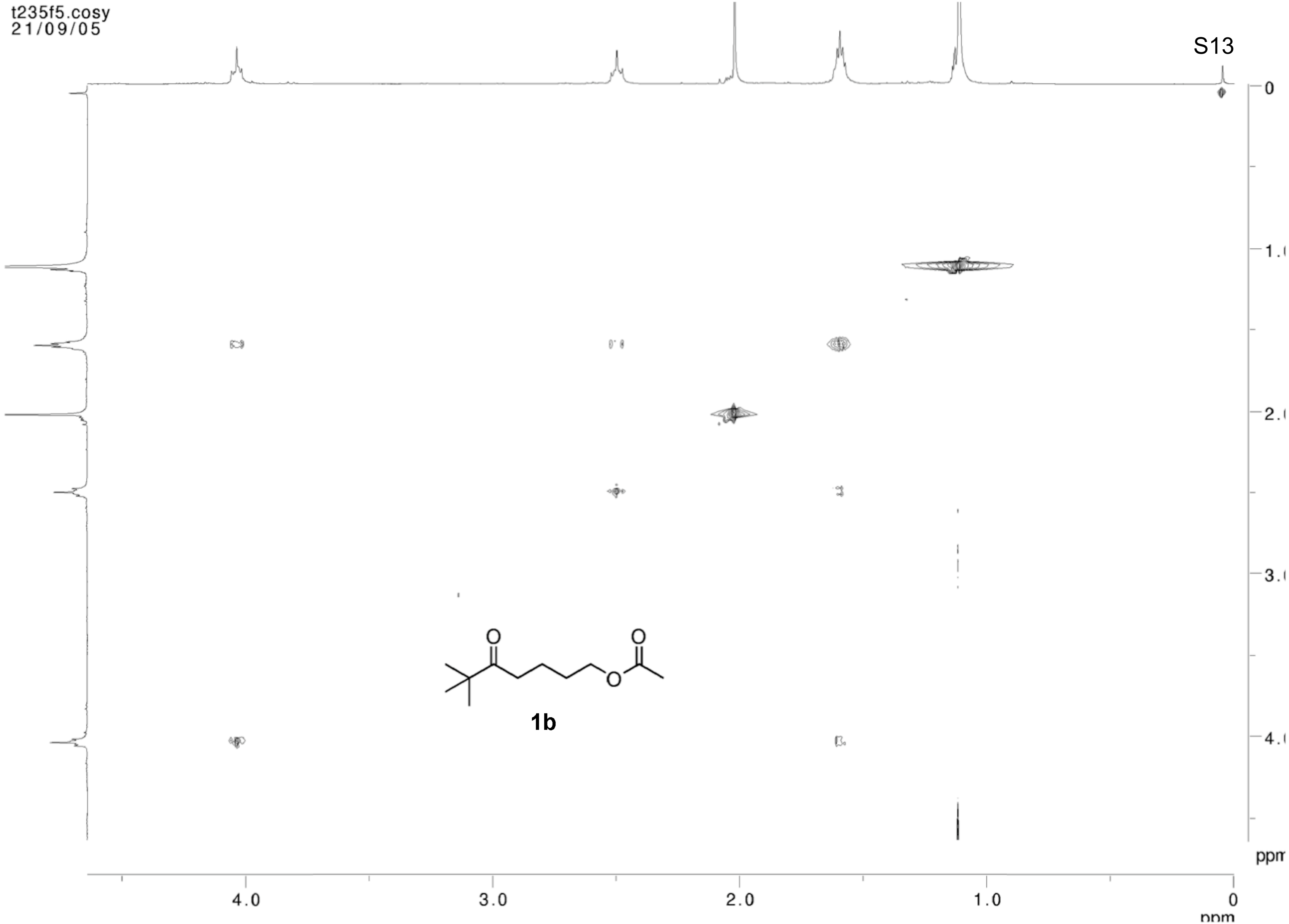
t235f5.cosy
21/09/05
1b
ppm
4.0
3.0
2.0
1.0
0
ppm

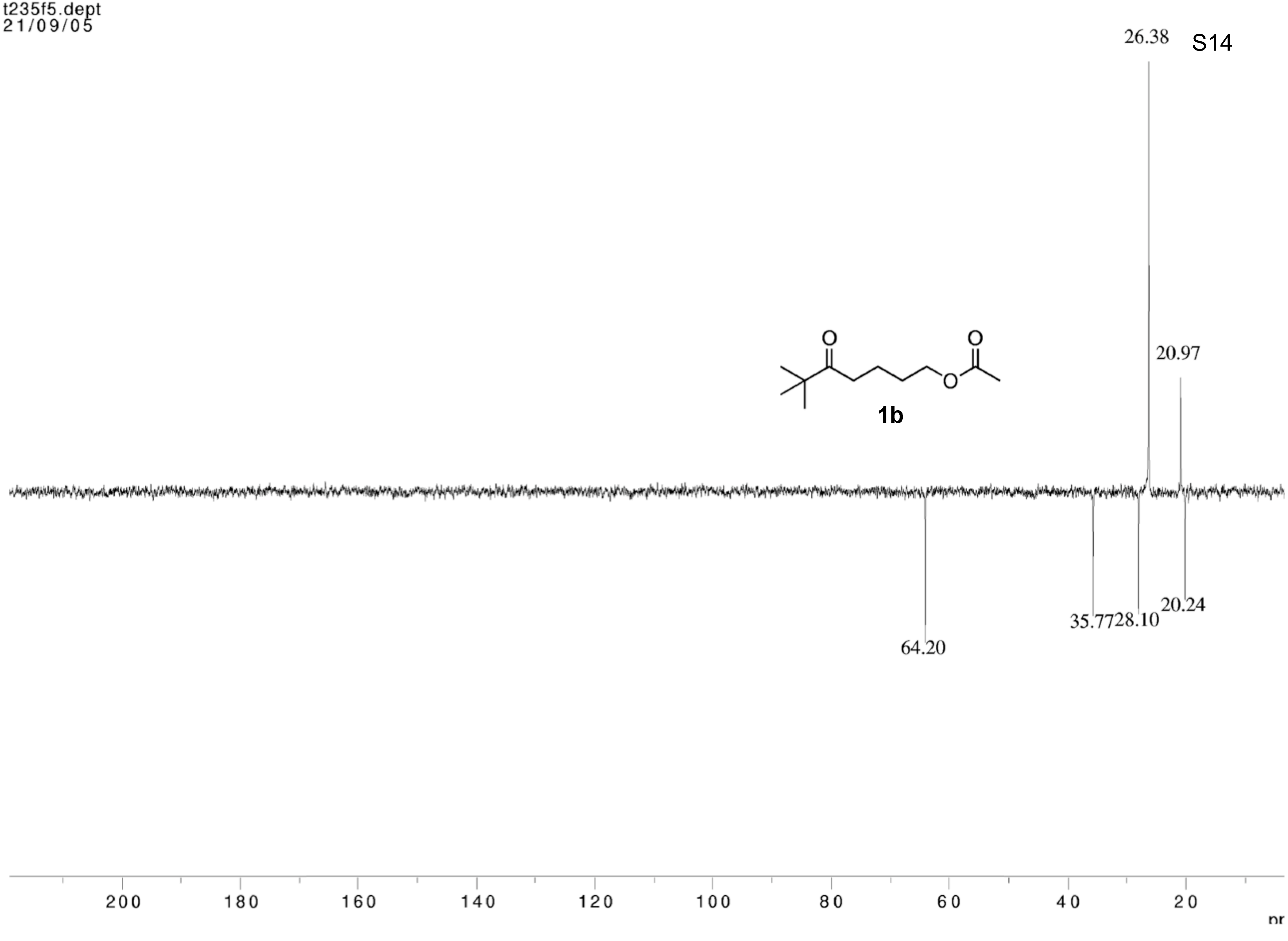
t235f5.dept
21/09/05
26.38
20.97
1b
64.20
35.77
28.10
20.24
200
180
160
140
120
100
80
60
40
20

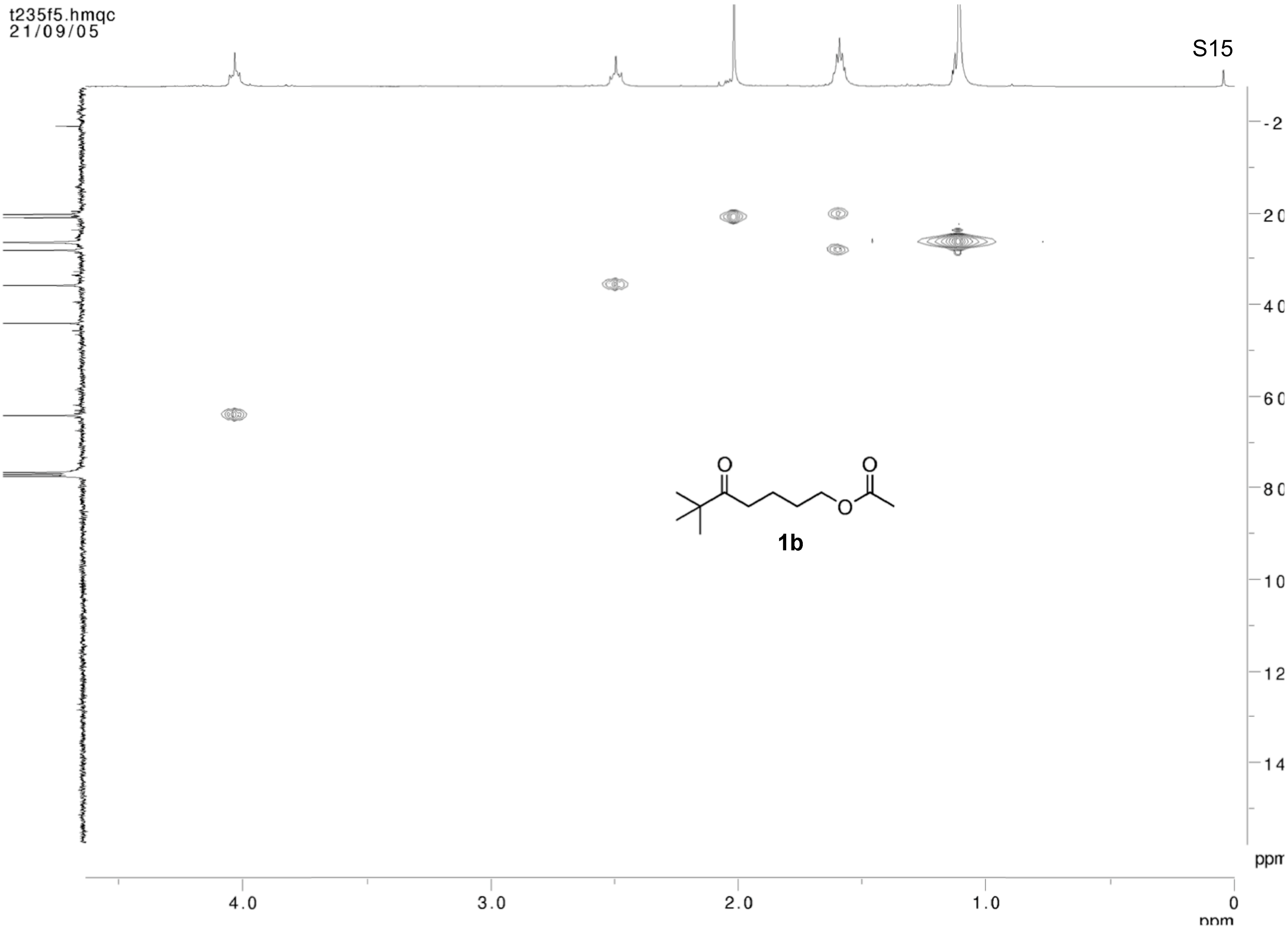
t235f5.hmqc
21/09/05
1b
-2
20
40
60
80
100
120
140
ppm
4.0
3.0
2.0
1.0
0
ppm

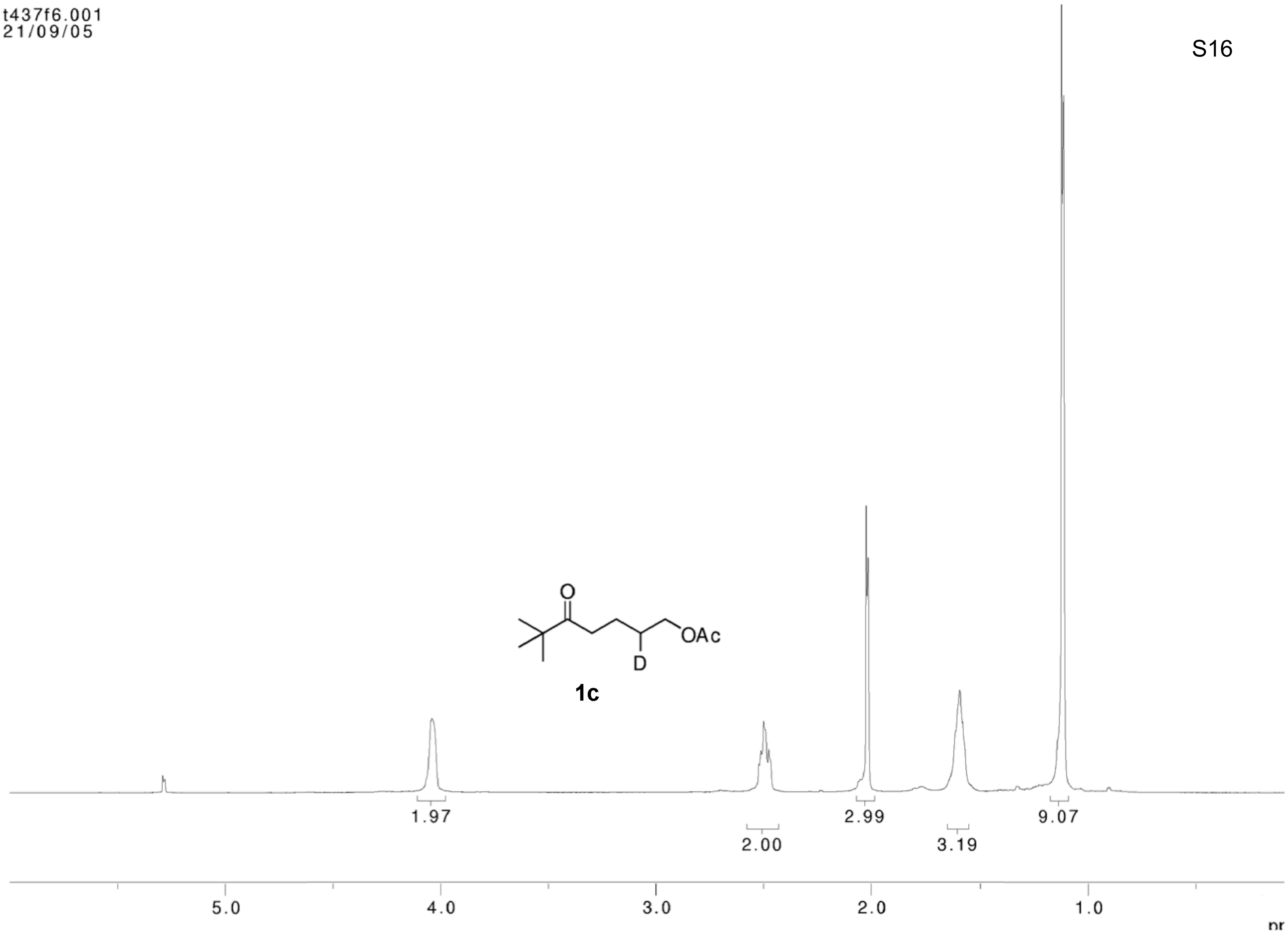
t437f6.001
21/09/05
O
OAc
D
1c
1.97
2.00
2.99
3.19
9.07
5.0
4.0
3.0
2.0
1.0

t437f6.013
21/09/05

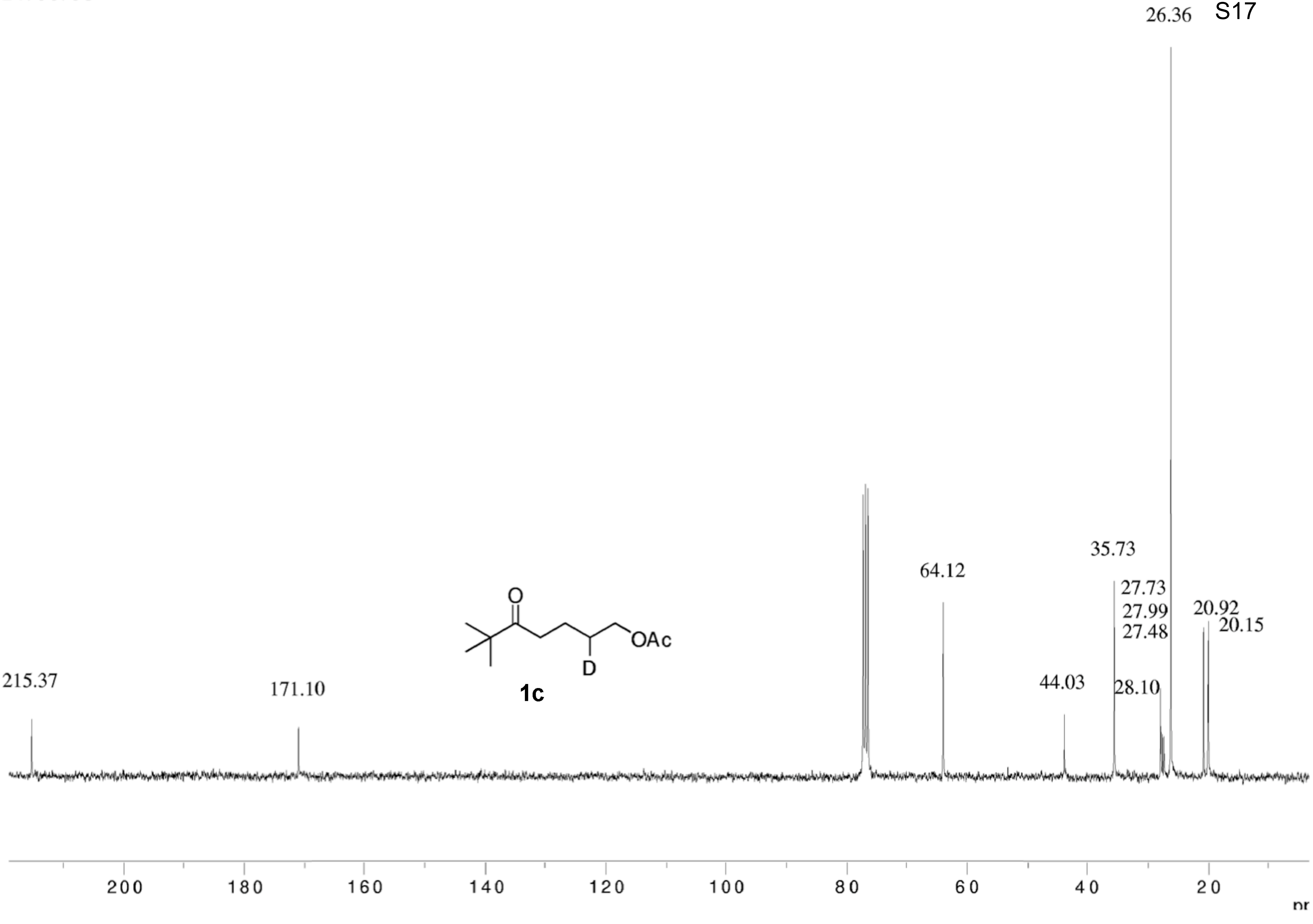

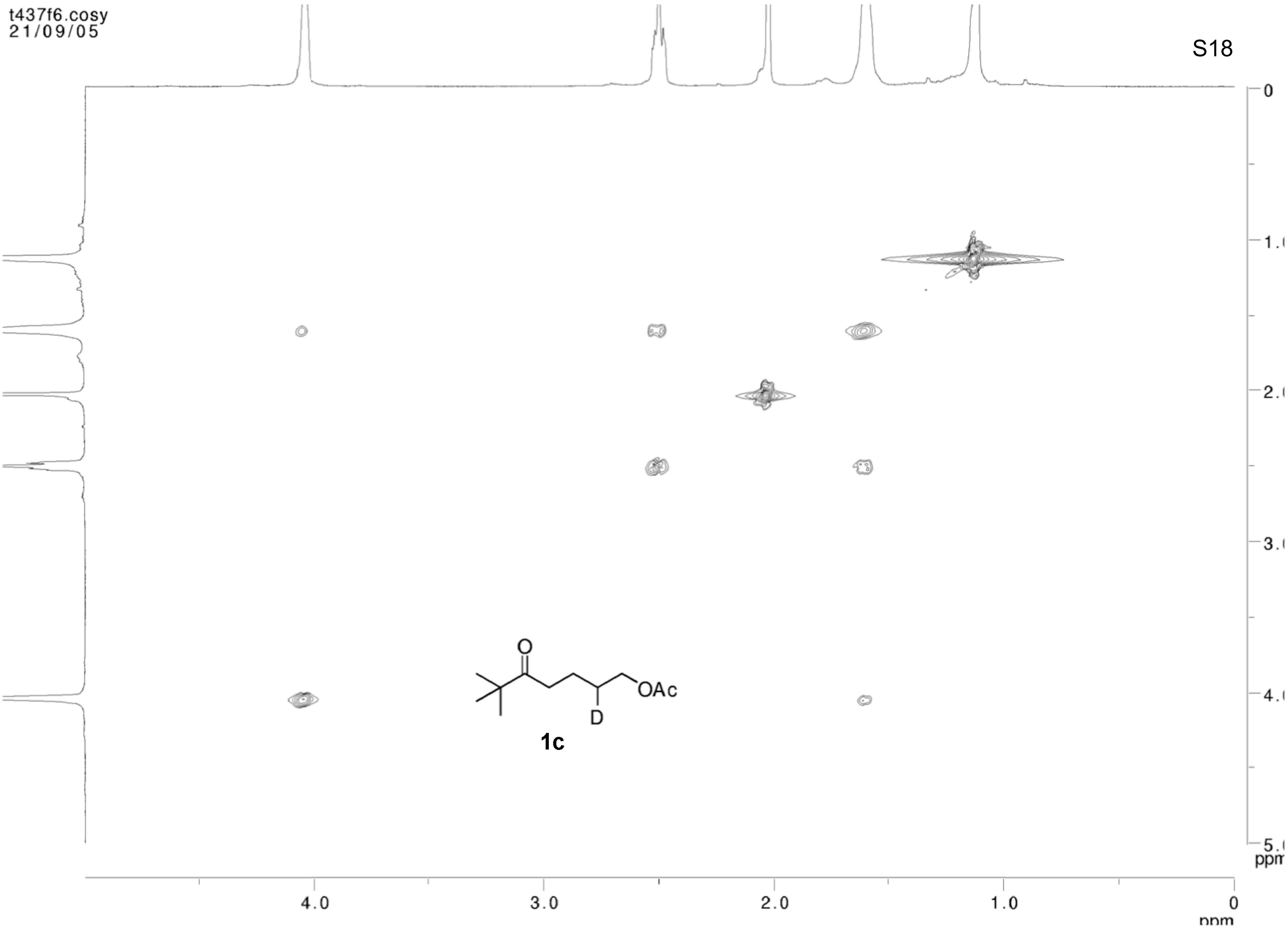
t437f6.cosy
21/09/05
O
OAc
D
1c
0
1.(
2.(
3.(
4.(
5.(
ppm
4.0
3.0
2.0
1.0
0
ppm

t437f6.dept
21/09/05

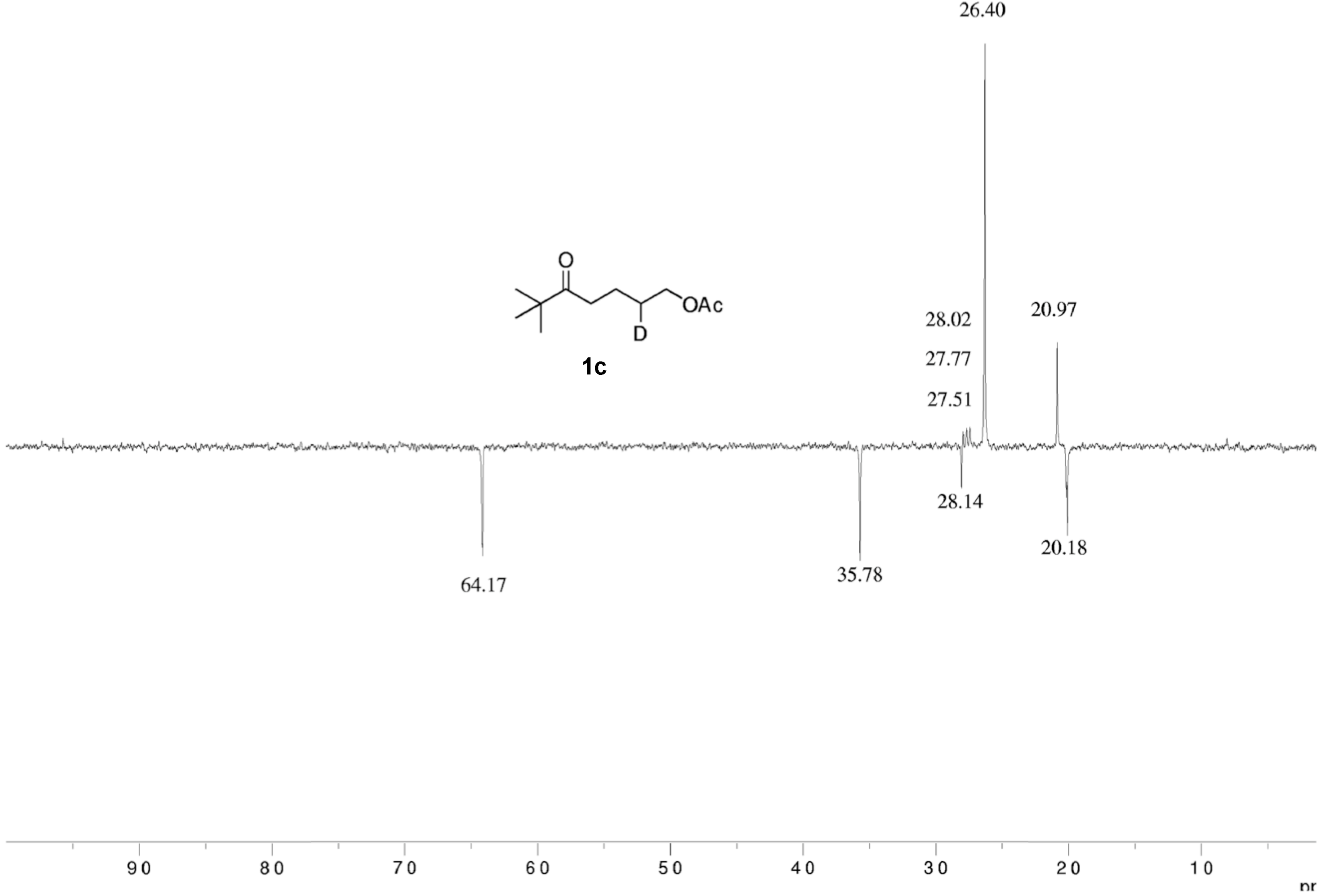

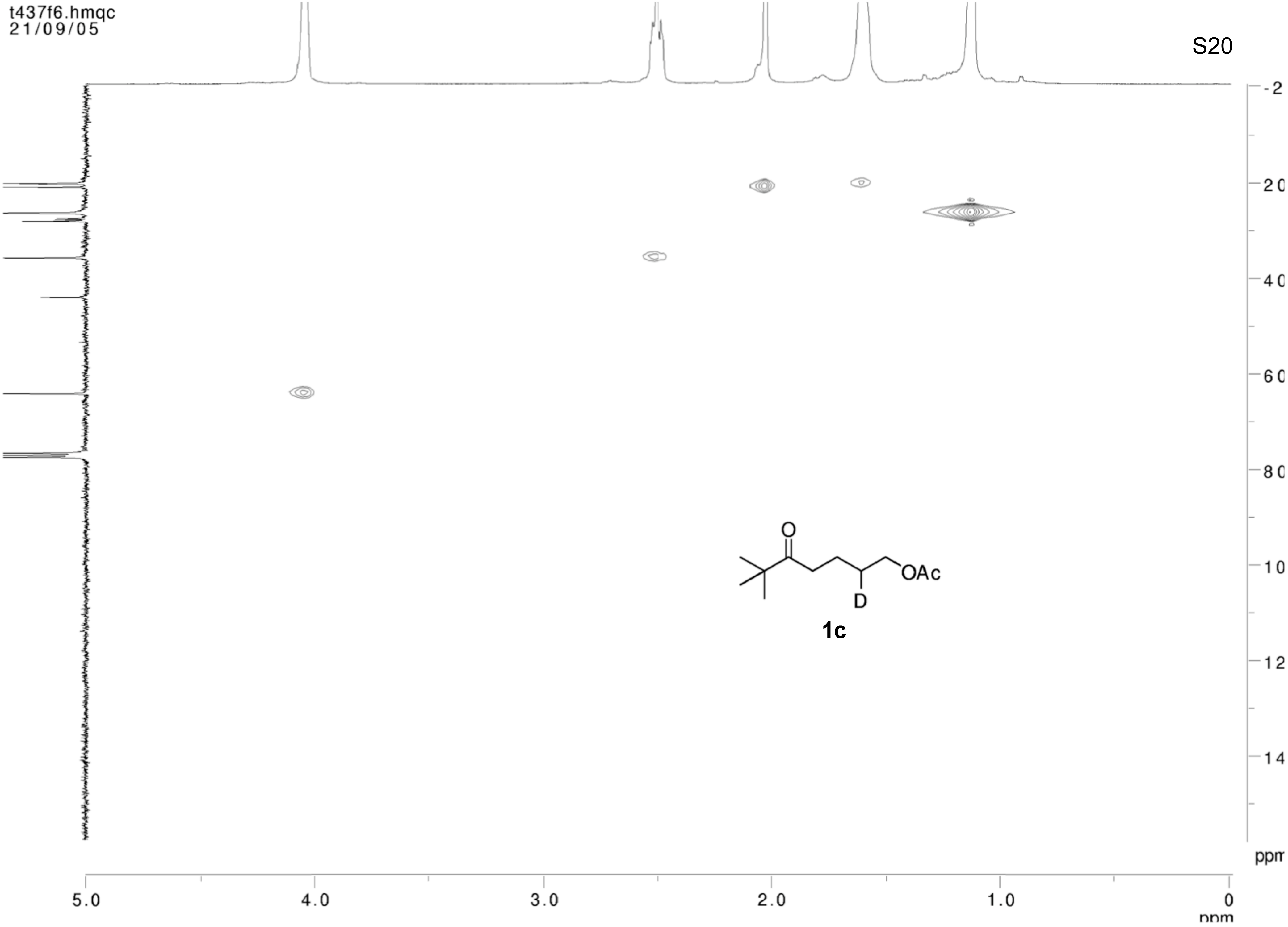
t437f6.hmqc
21/09/05
O
OAc
D
1c
ppm
5.0
4.0
3.0
2.0
1.0
0
ppm

t437f6.deut
21/09/05

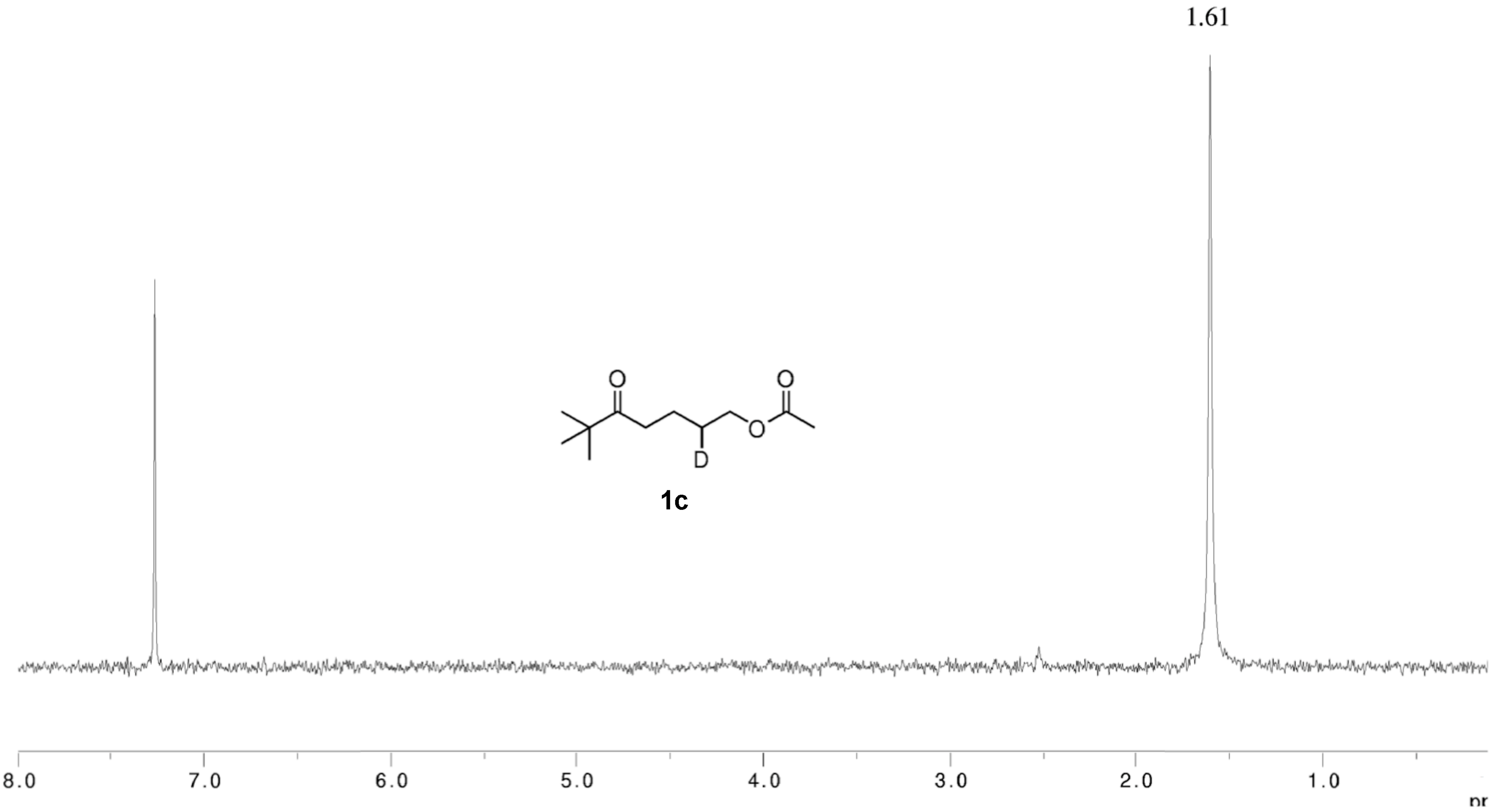

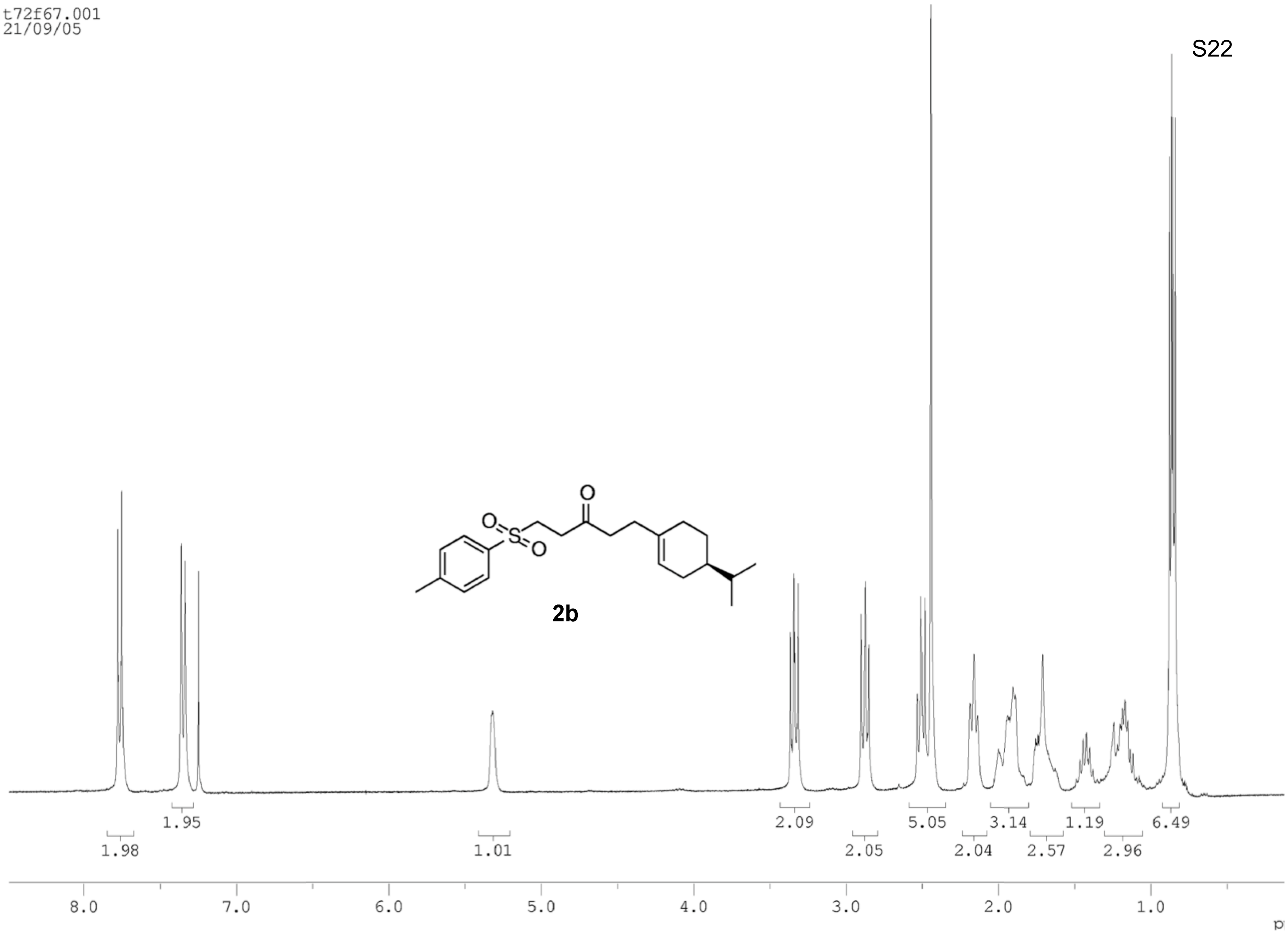
t72f67.001
21/09/05
2b
1.98
1.95
1.01
2.09
2.05
5.05
2.04
3.14
2.57
1.19
2.96
6.49
8.0
7.0
6.0
5.0
4.0
3.0
2.0
1.0

t72f67.013
21/09/05

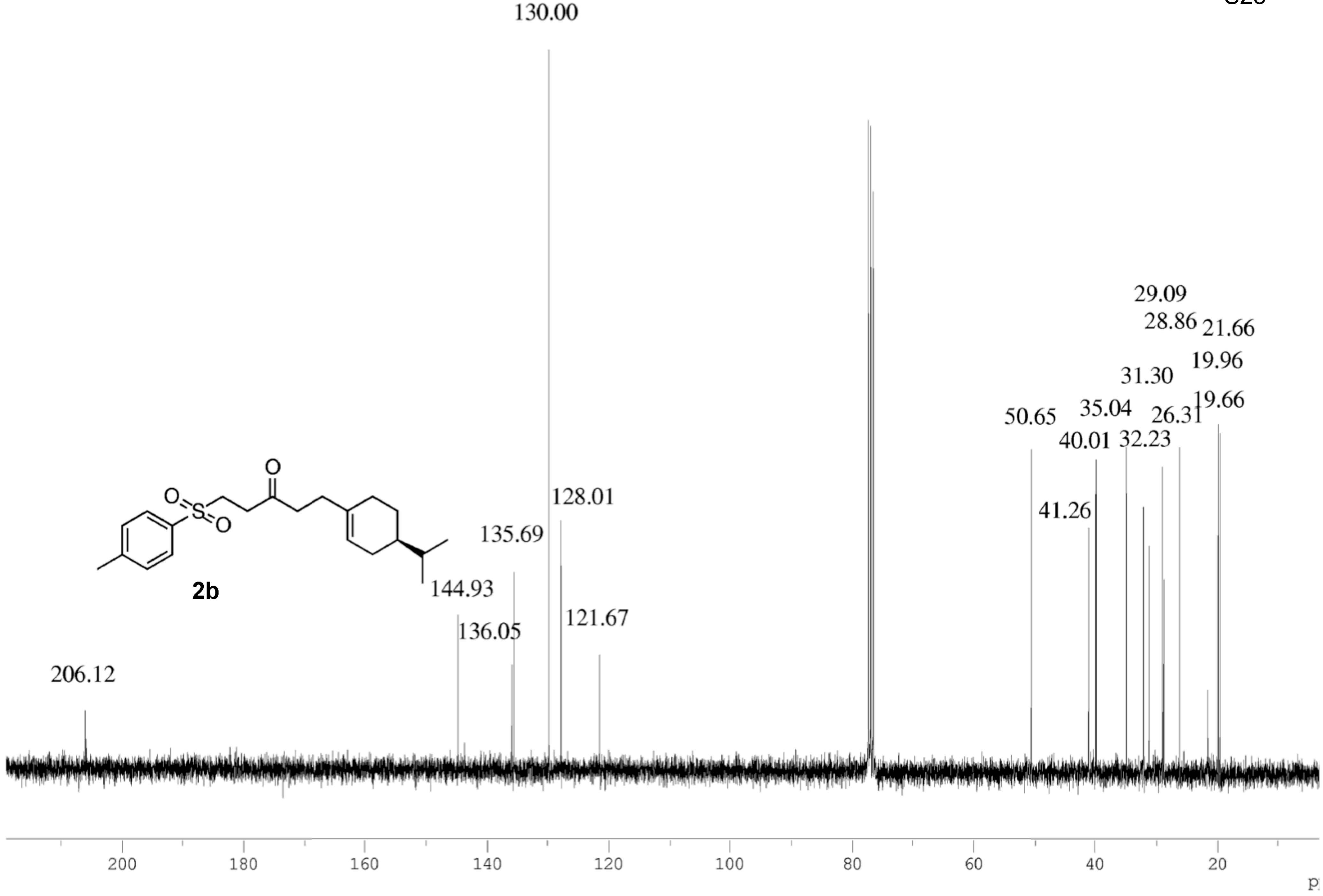

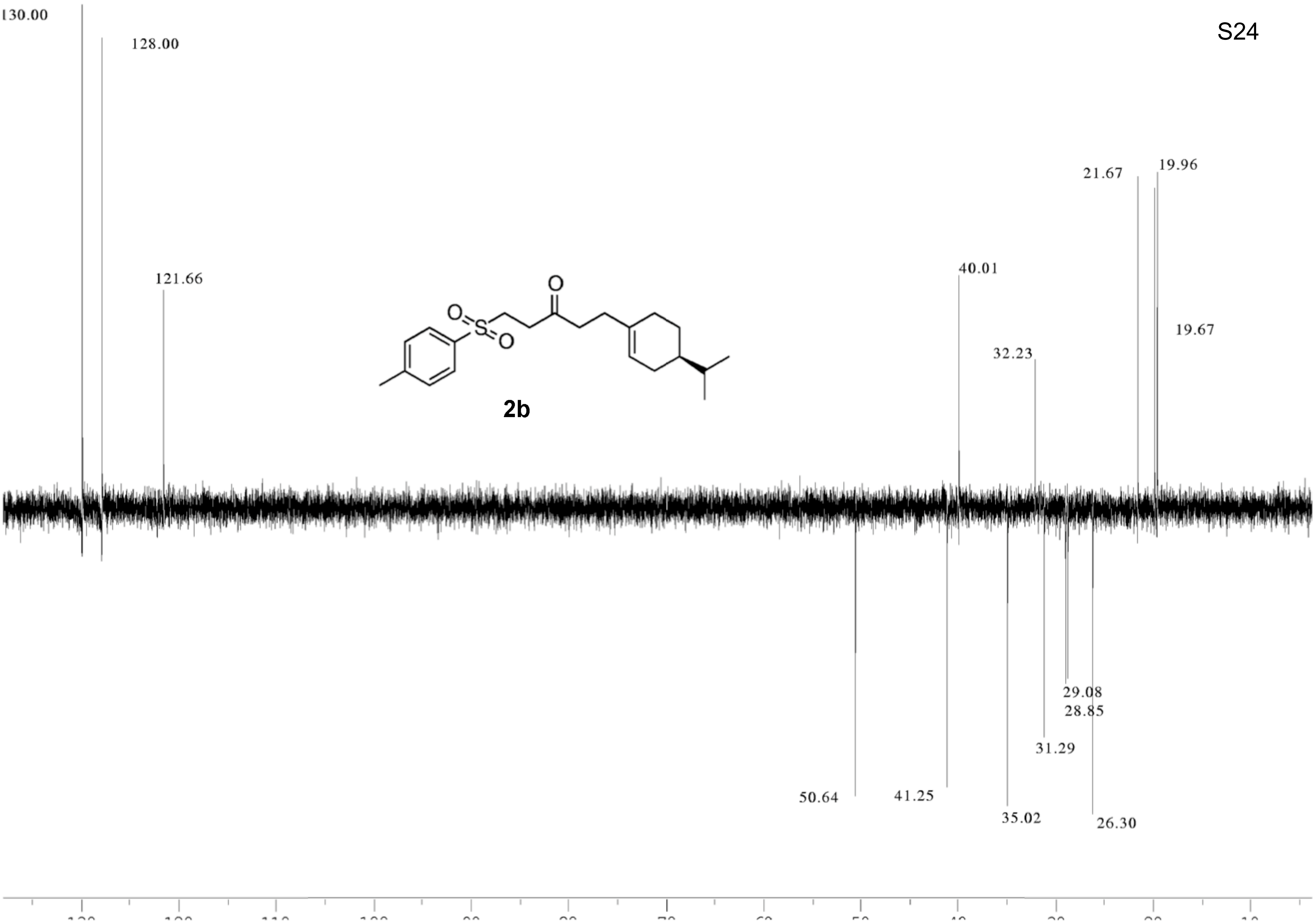
130.00
128.00
121.66
2b
40.01
32.23
21.67
19.96
19.67
50.64
41.25
35.02
31.29
29.08
28.85
26.30

t72f67.hmqc
21/09/05

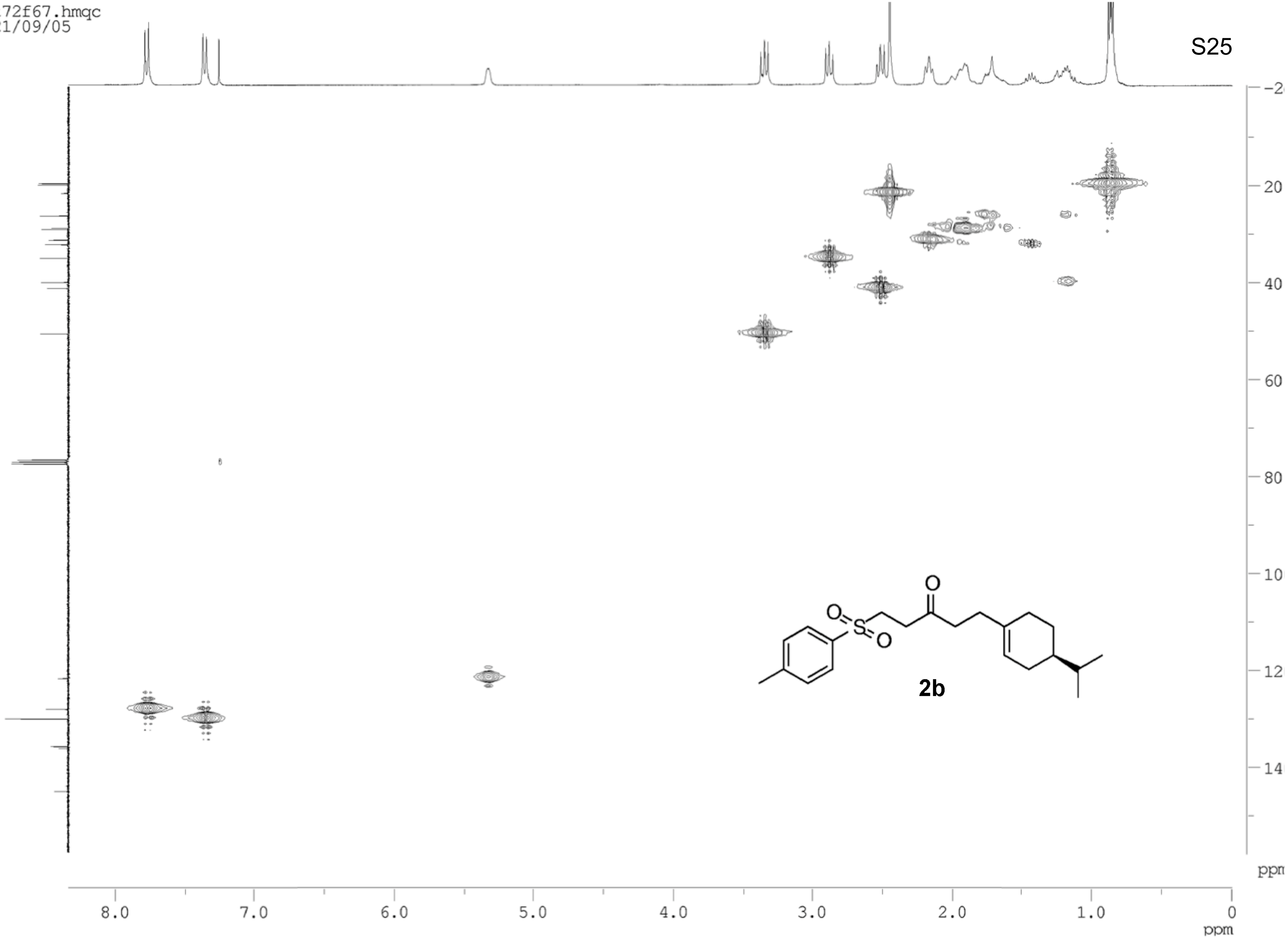

2b

t449f11.001
23/09/05

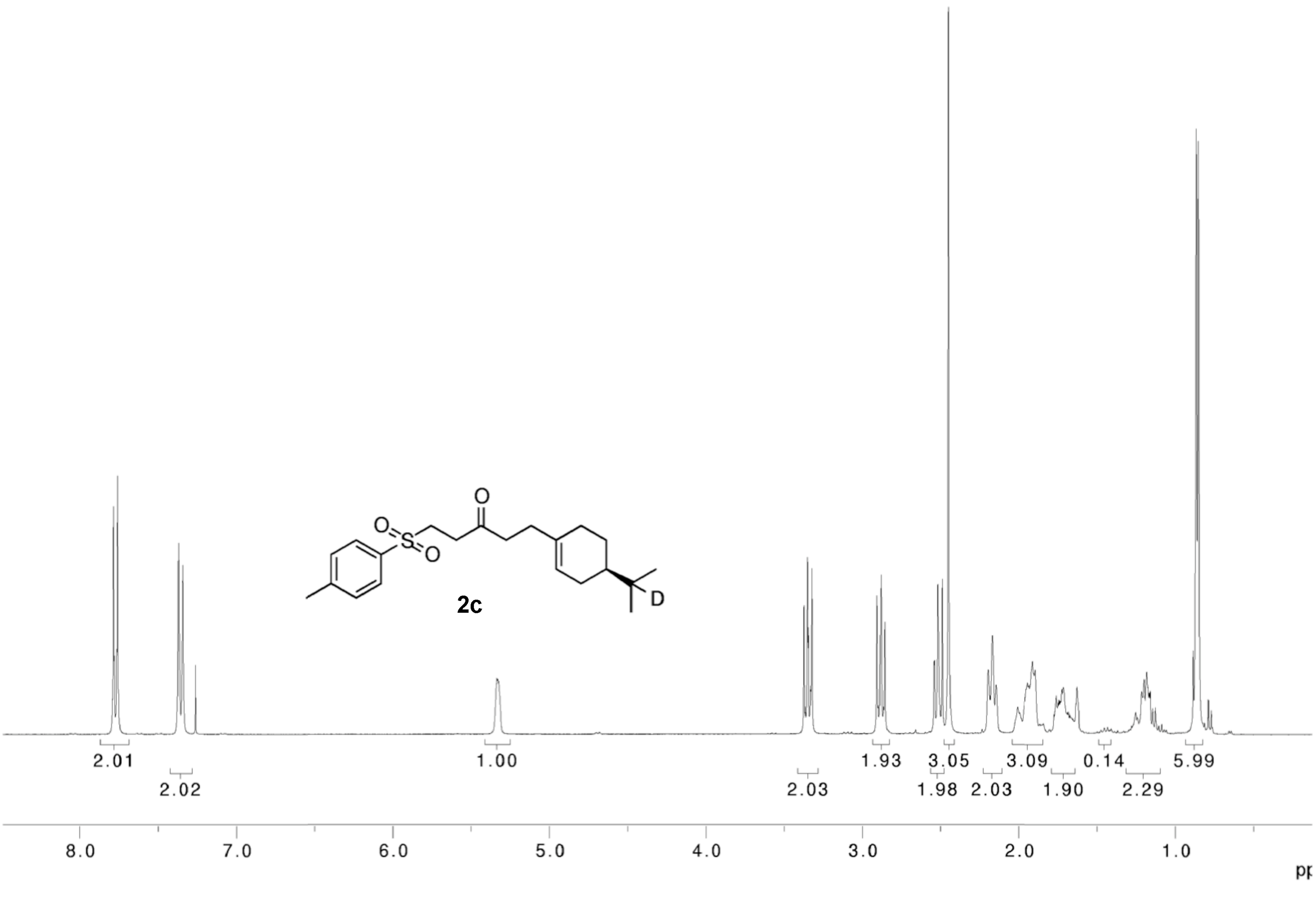

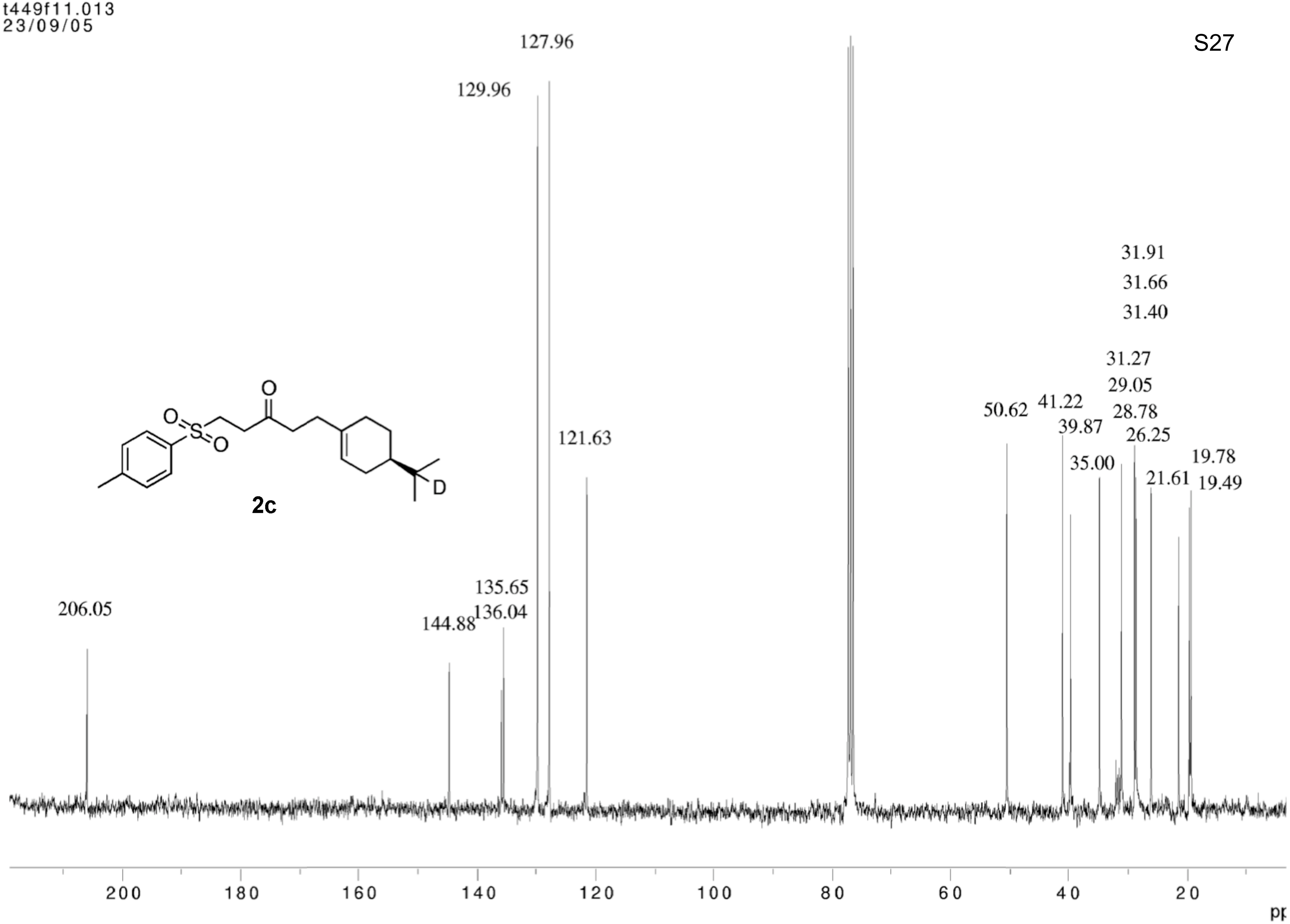
t449f11.013
23/09/05
127.96
129.96
121.63
135.65
136.04
144.88
206.05
50.62
41.22
39.87
35.00
31.91
31.66
31.40
31.27
29.05
28.78
26.25
21.61
19.78
19.49
O
O
S
O
D
2c
200
180
160
140
120
100
80
60
40
20
pp

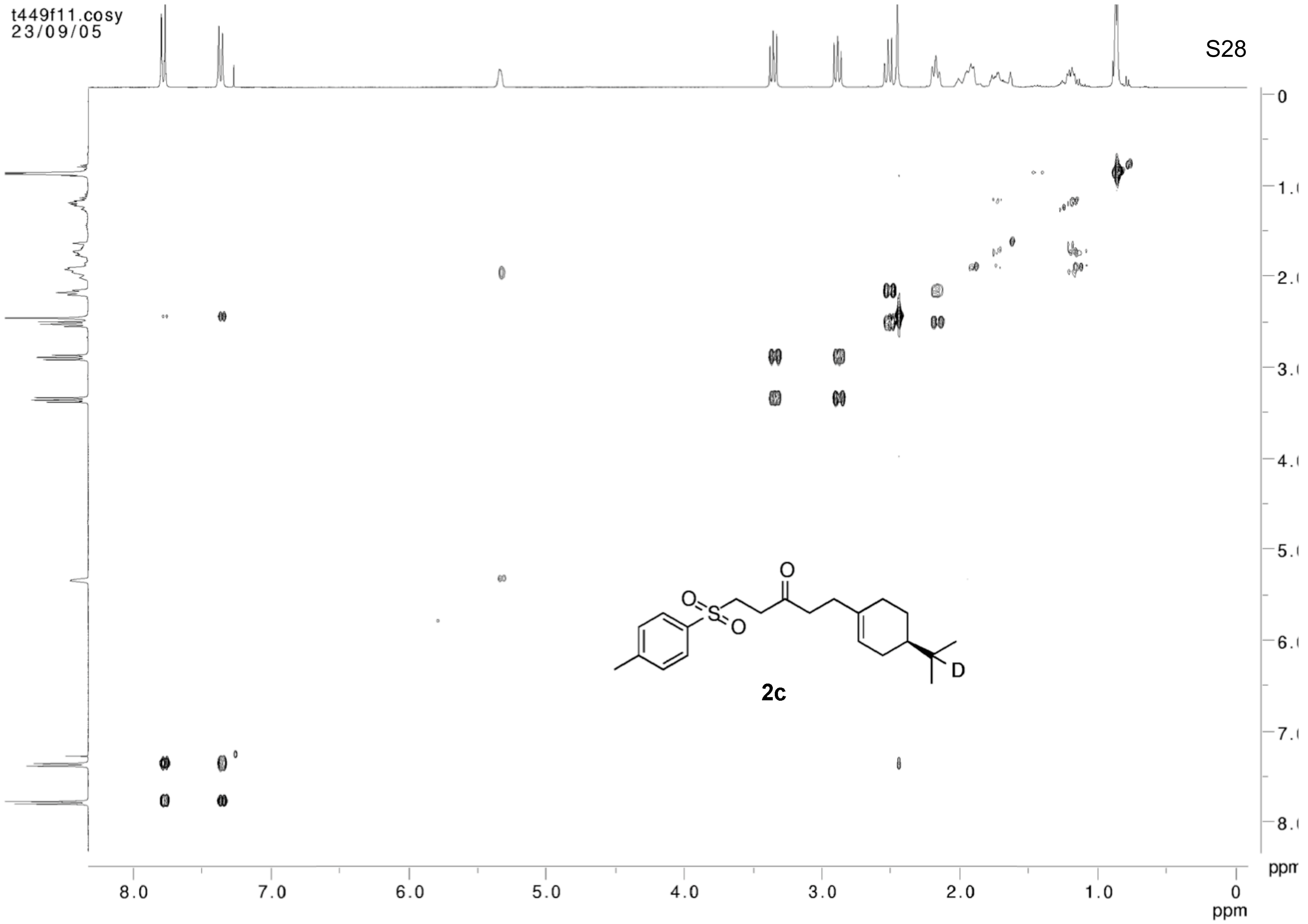
t449f11.cosy
23/09/05
0
8.0
7.0
6.0
5.0
4.0
3.0
2.0
1.0
ppm
2c
O
S
D

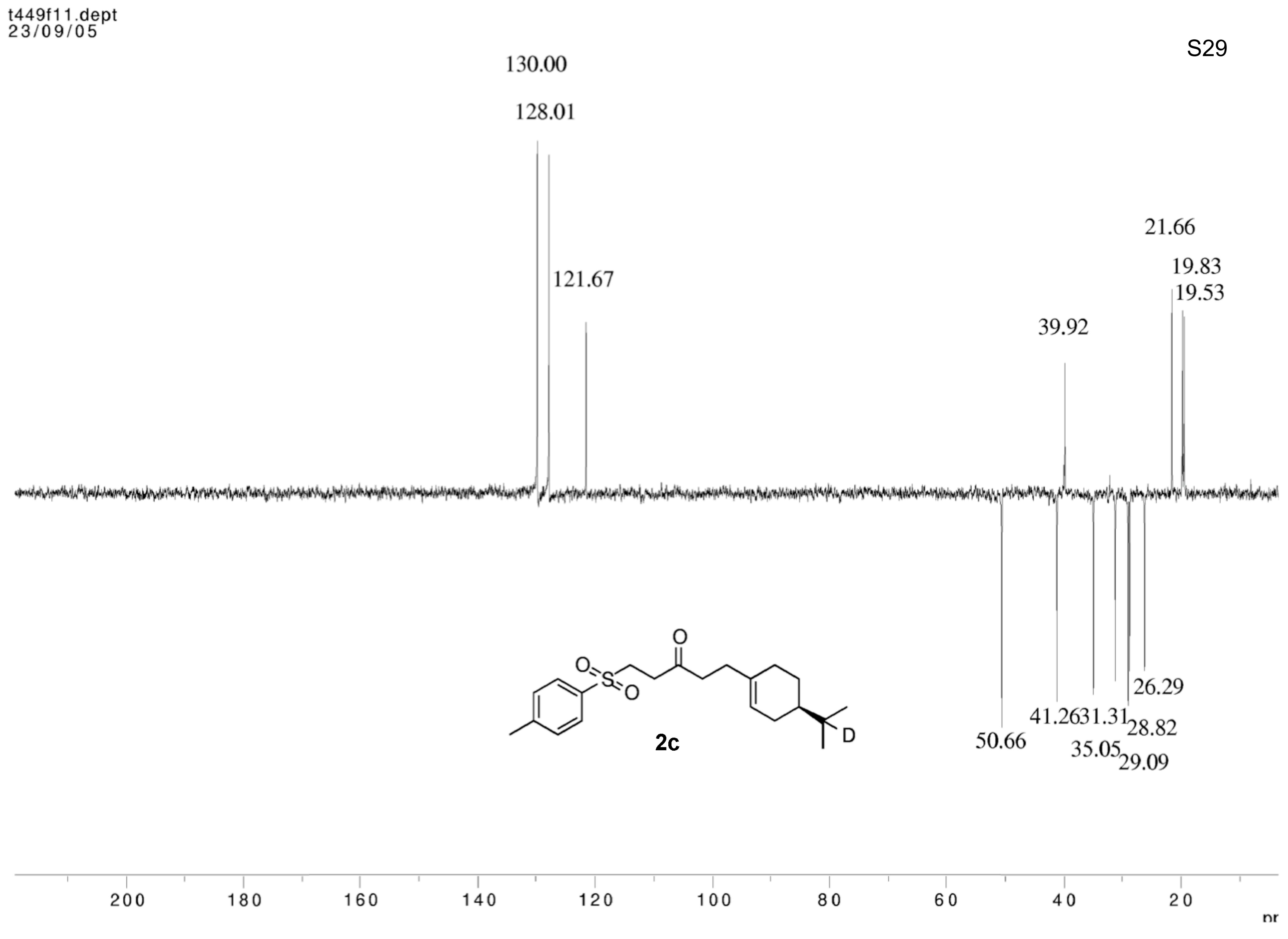
t449f11.dept
23/09/05
130.00
128.01
121.67
39.92
21.66
19.83
19.53
50.66
41.26
31.31
35.05
29.09
28.82
26.29
2c
O
S
D
200 180 160 140 120 100 80 60 40 20
pr

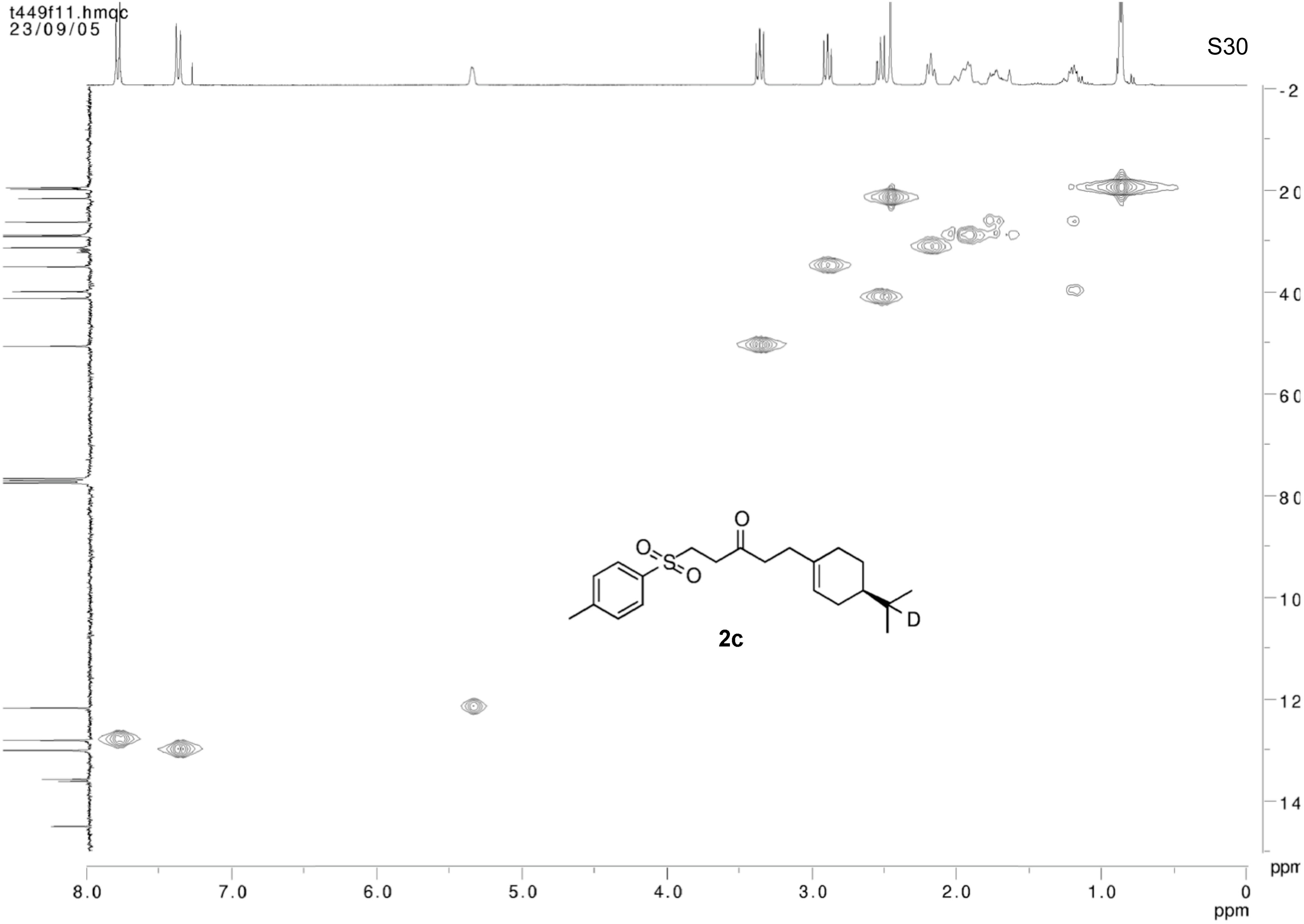
t449f11.hmqc
23/09/05
2c
D
O
S
ppm
8.0
7.0
6.0
5.0
4.0
3.0
2.0
1.0
0
ppm
20
40
60
80
100
120
140

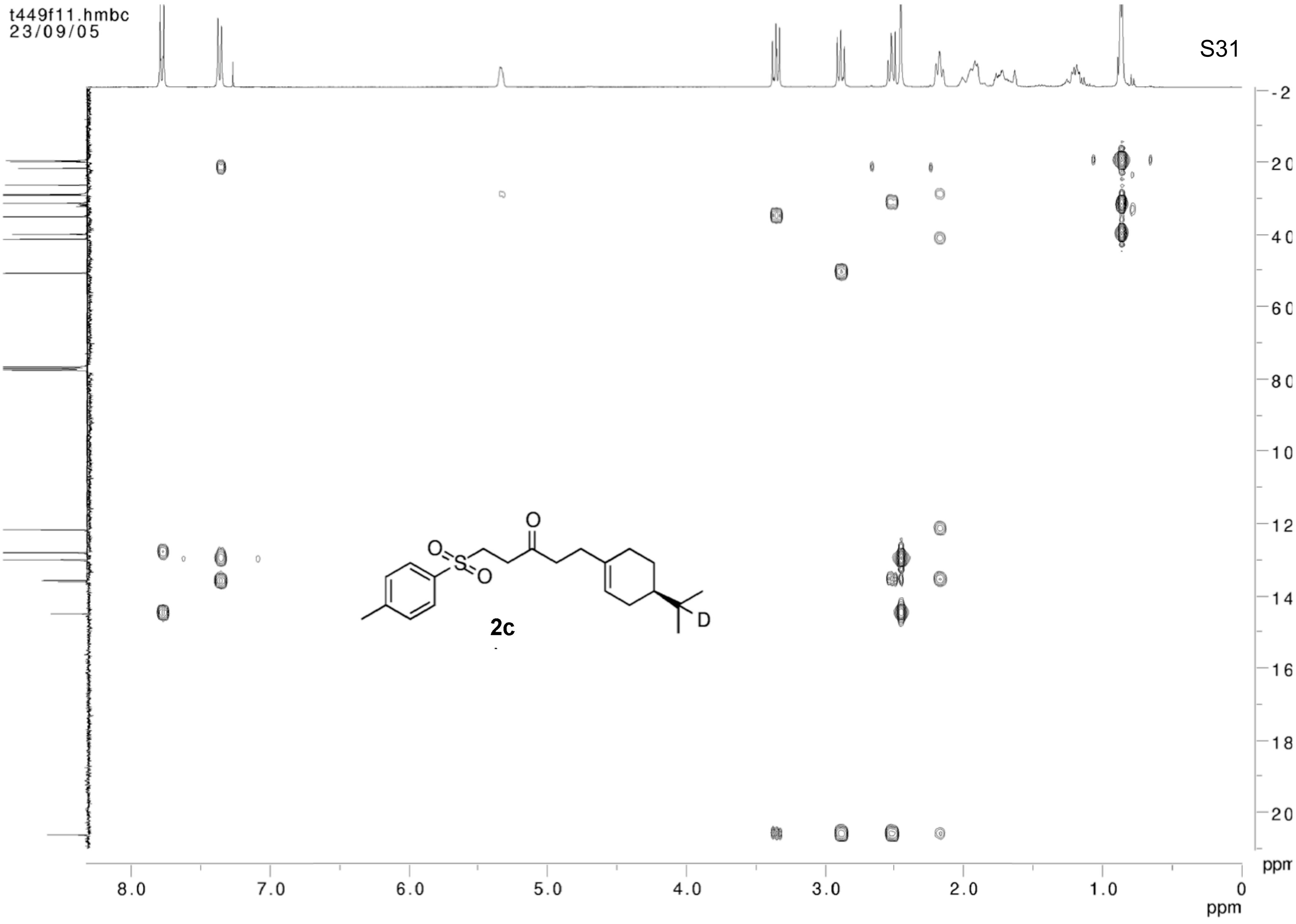
t449f11.hmbc
23/09/05
2c
D
O
S
8.0
7.0
6.0
5.0
4.0
3.0
2.0
1.0
0
ppm
ppm

t449f11.deut
21/09/05

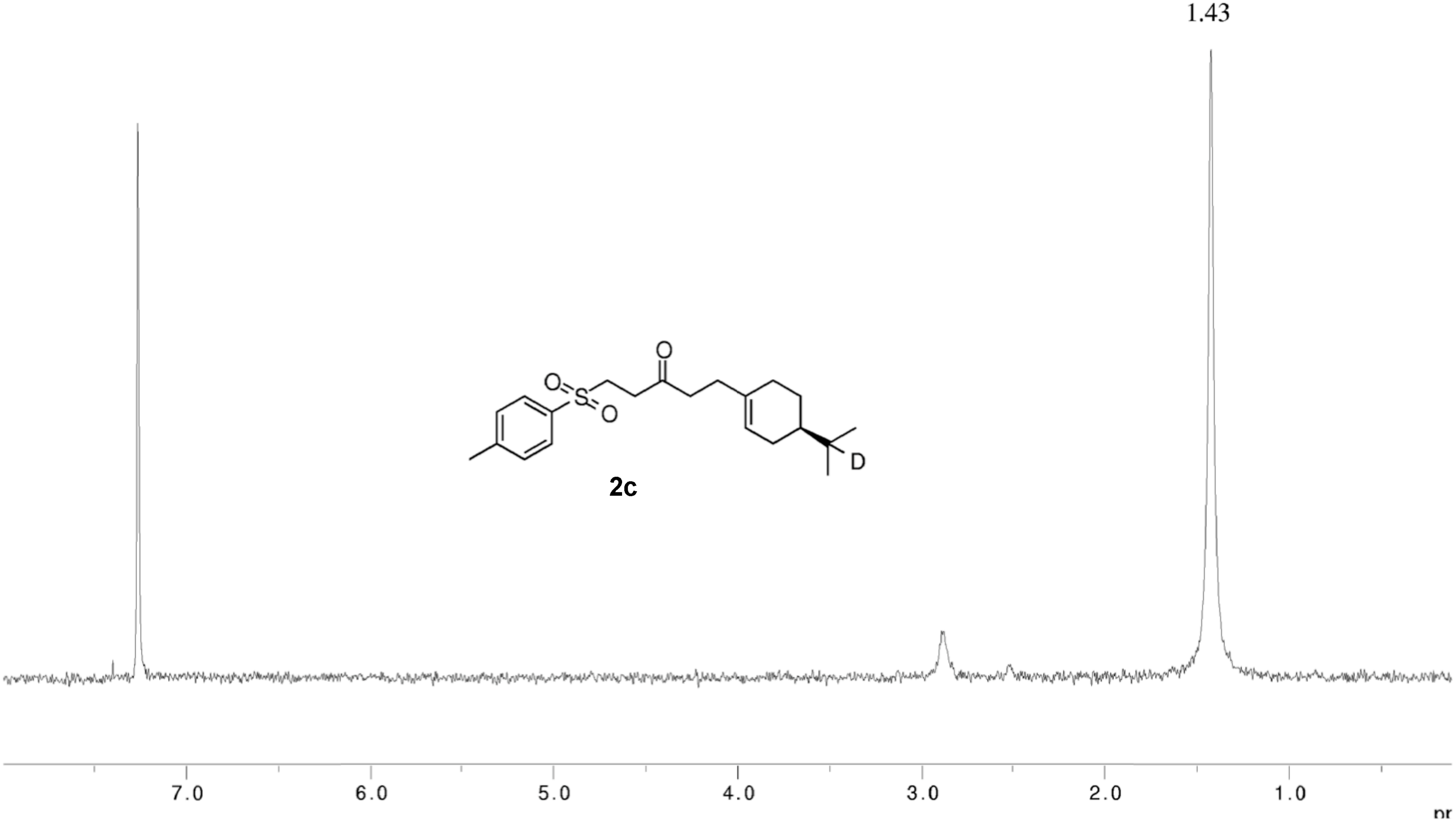

22FAL70 1 (1D 1H) CDCl3 300MHz

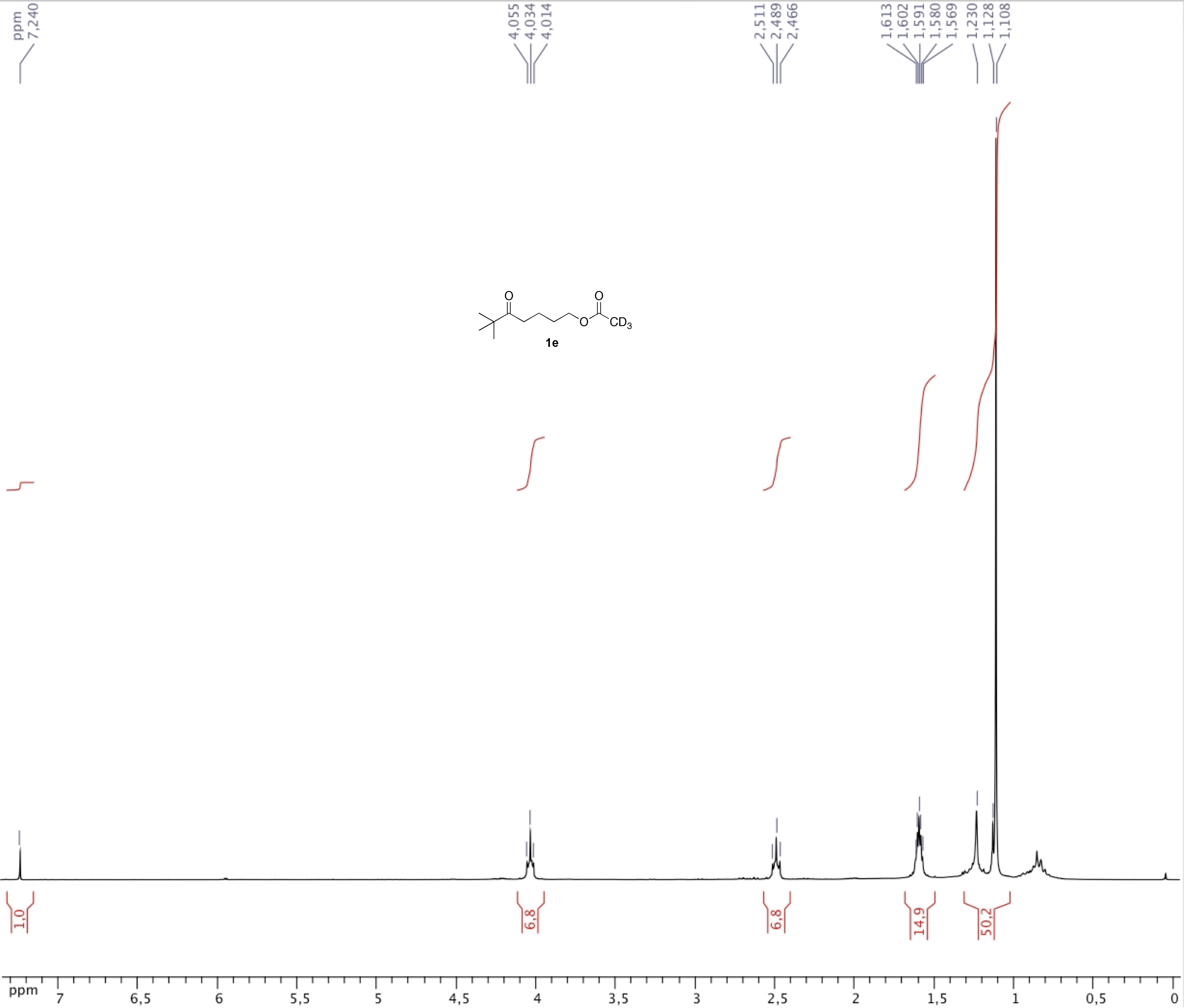

Parameters

22FAL70 1 (1D 1H)

F1 :
- Size : 65536 points complex
- Spectral Width : 6172.8394 Hz
- Carrier Frequency : 300.17184 MHz
- Nucleus : 1H

Type : 1D
Spectro : BRUKER 300 MHz
Probe : 5 mm QNP 1H/13C/31P/19F Z-GR
Date : Wed Apr 04 19:56:42 CEST 2007
Temperature : 300.0 K
Solvent : CDCl3

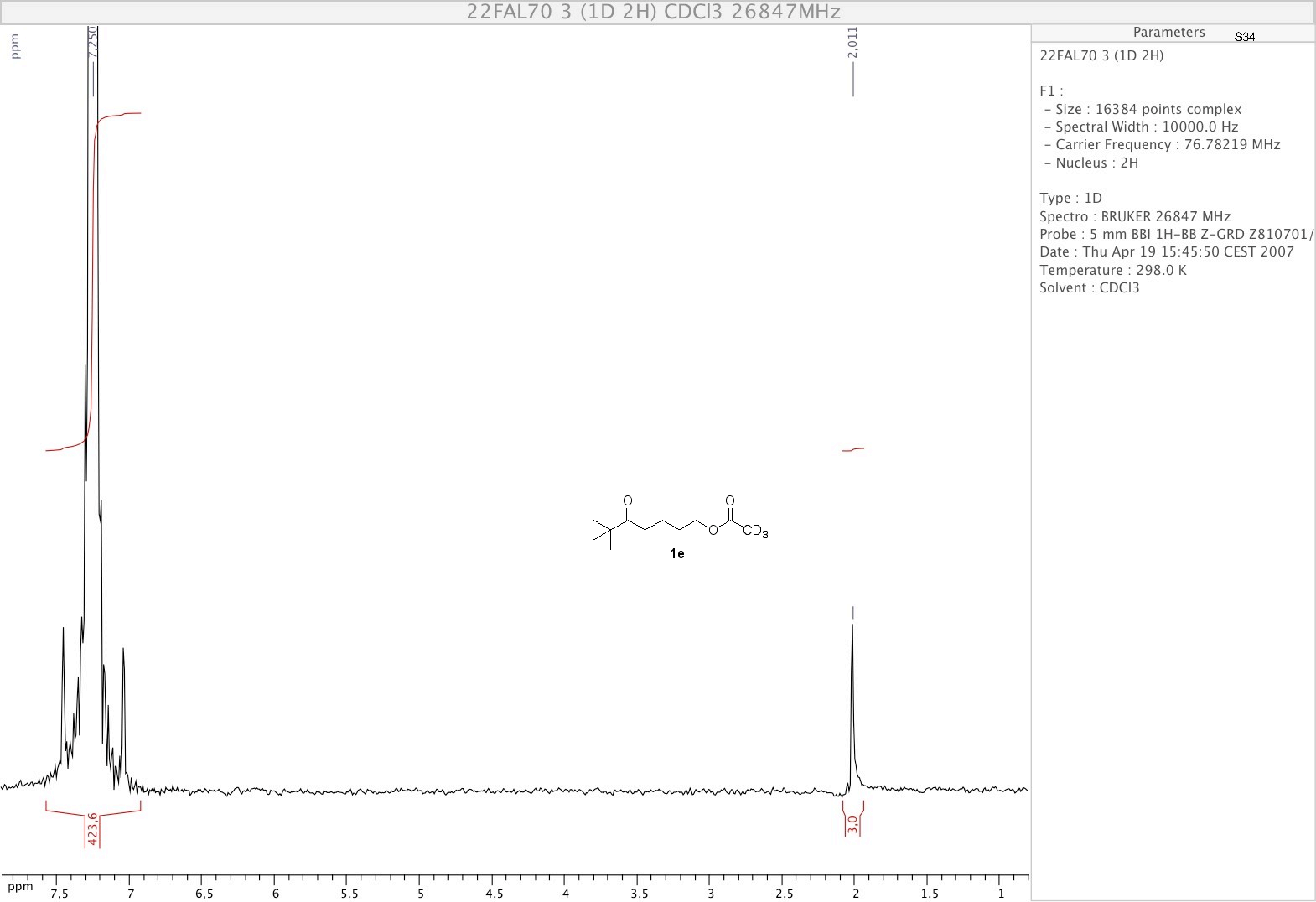
22FAL70 3 (1D 2H) CDCl3 26847MHz
ppm
7,250
2,011
423,6
3,0
O
O
O
CD3
1e
ppm 7,5 7 6,5 6 5,5 5 4,5 4 3,5 3 2,5 2 1,5 1
Parameters
22FAL70 3 (1D 2H)
F1 :
- Size : 16384 points complex
- Spectral Width : 10000.0 Hz
- Carrier Frequency : 76.78219 MHz
- Nucleus : 2H
Type : 1D
Spectro : BRUKER 26847 MHz
Probe : 5 mm BBI 1H-BB Z-GRD Z810701/
Date : Thu Apr 19 15:45:50 CEST 2007
Temperature : 298.0 K
Solvent : CDCl3

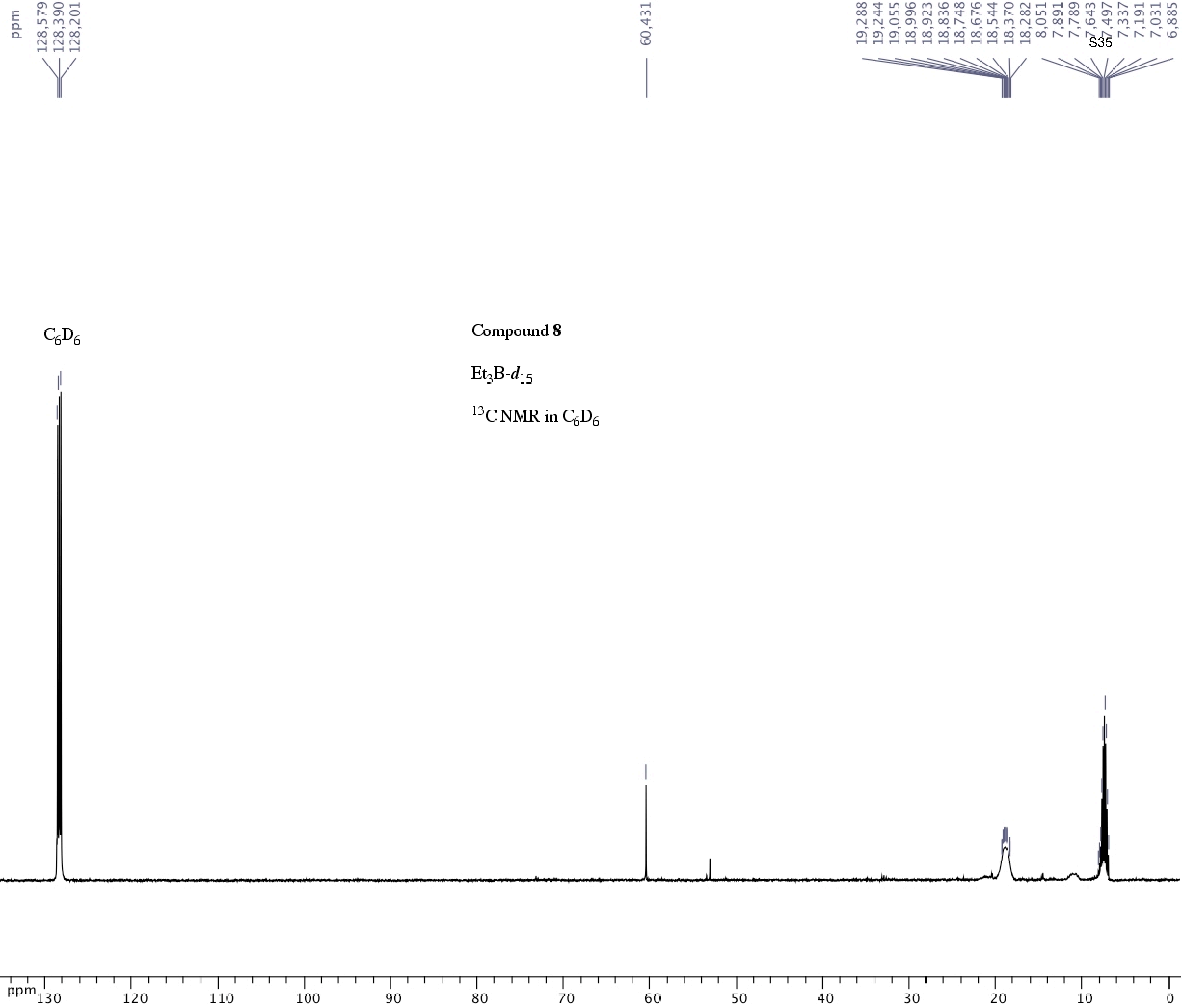

ppm
128,579
128,390
128,201
60,431
19,288
19,244
19,055
18,996
18,923
18,836
18,748
18,676
18,544
18,370
18,282
8,051
7,891
7,789
7,643
7,497
7,337
7,191
7,031
6,885
C6D6
Compound 8
Et3B-d15
13C NMR in C6D6
ppm
130
120
110
100
90
80
70
60
50
40
30
20
10
0

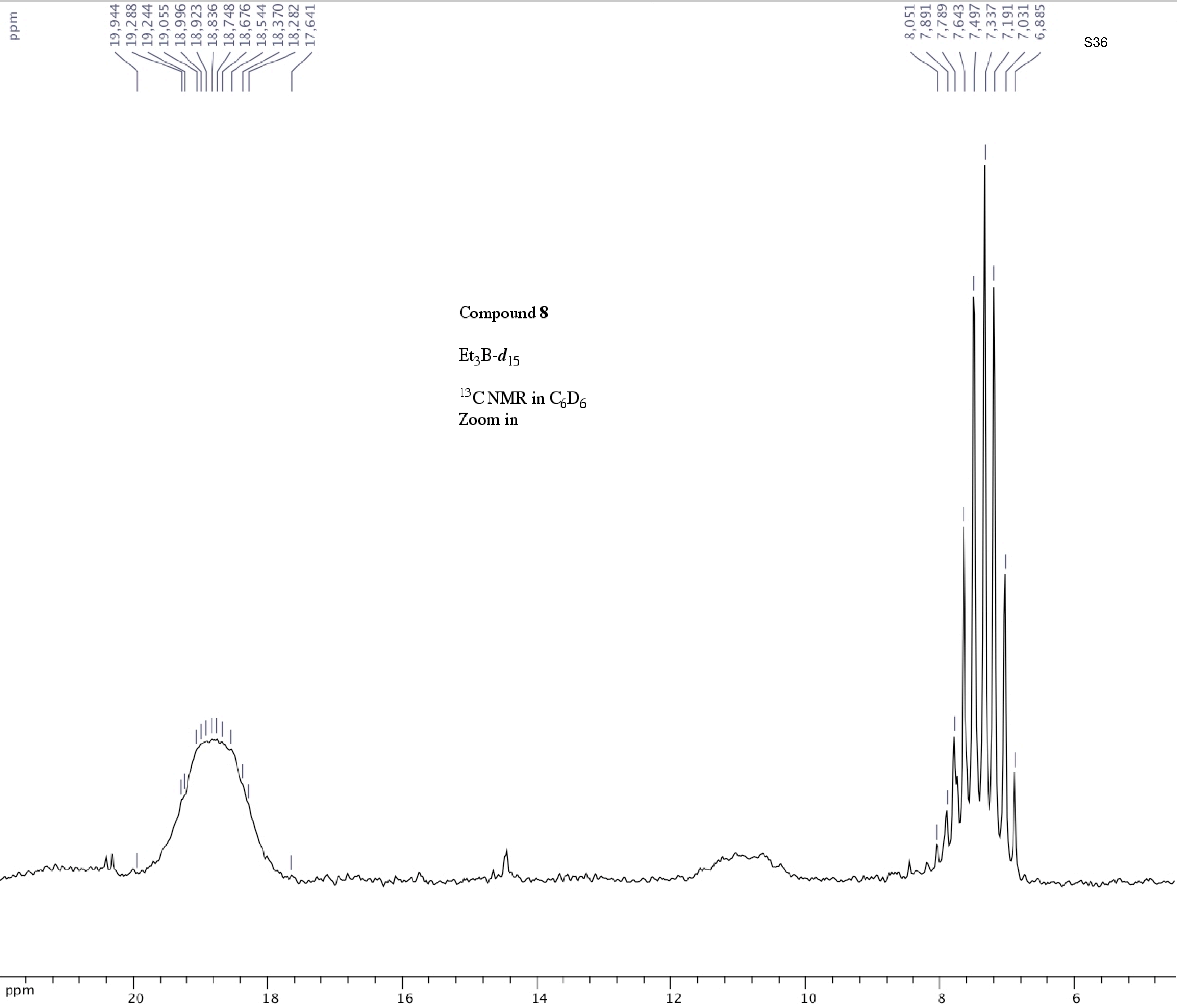

Compound 8

Et_3B-d_{15}

^{13}C NMR in C_6D_6
Zoom in

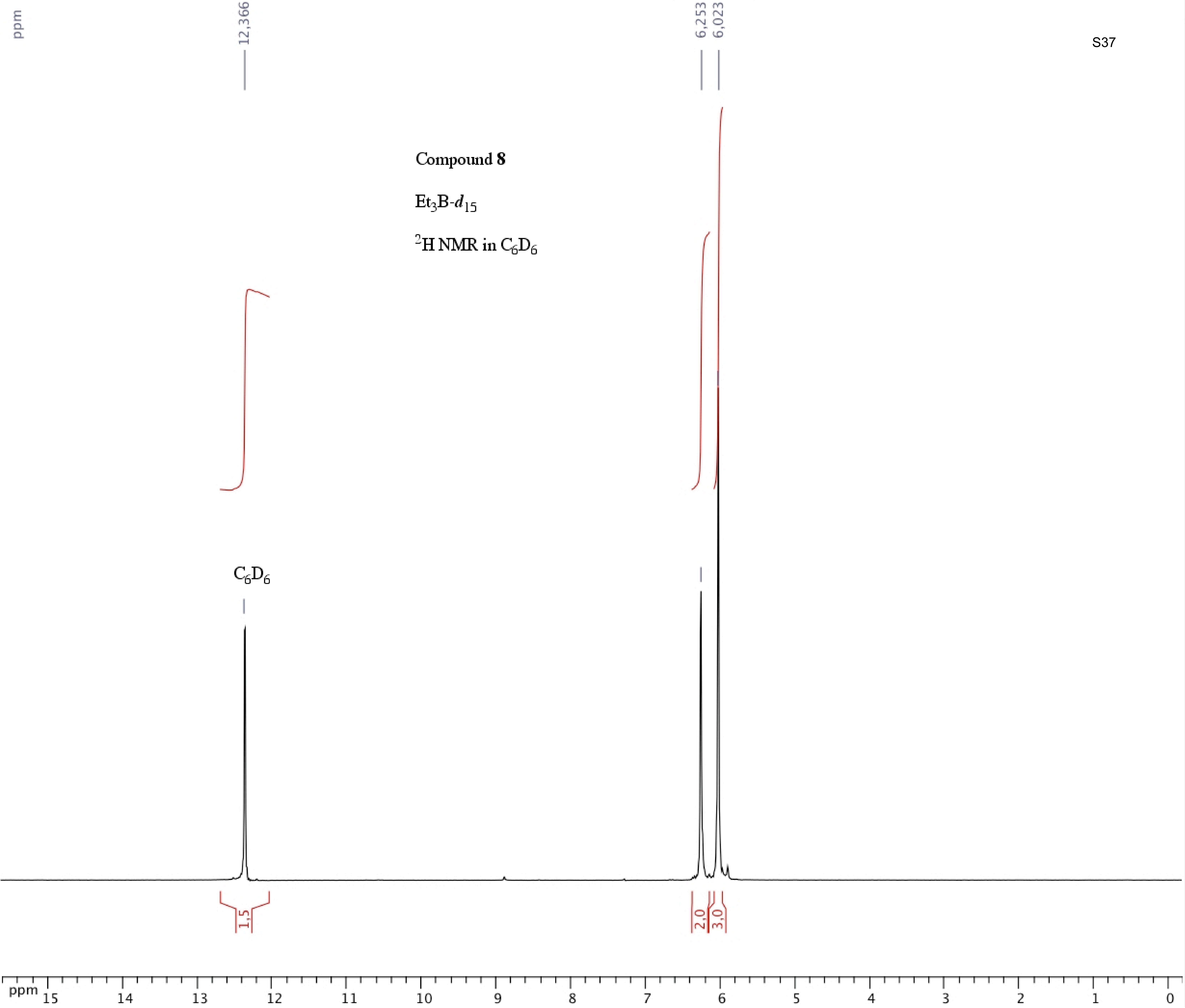

ppm
12,366
6,253
6,023
Compound **8**
Et_3B-d_{15}
^2H NMR in C_6D_6
C_6D_6
1,5
2,0
3,0
ppm 15 14 13 12 11 10 9 8 7 6 5 4 3 2 1 0

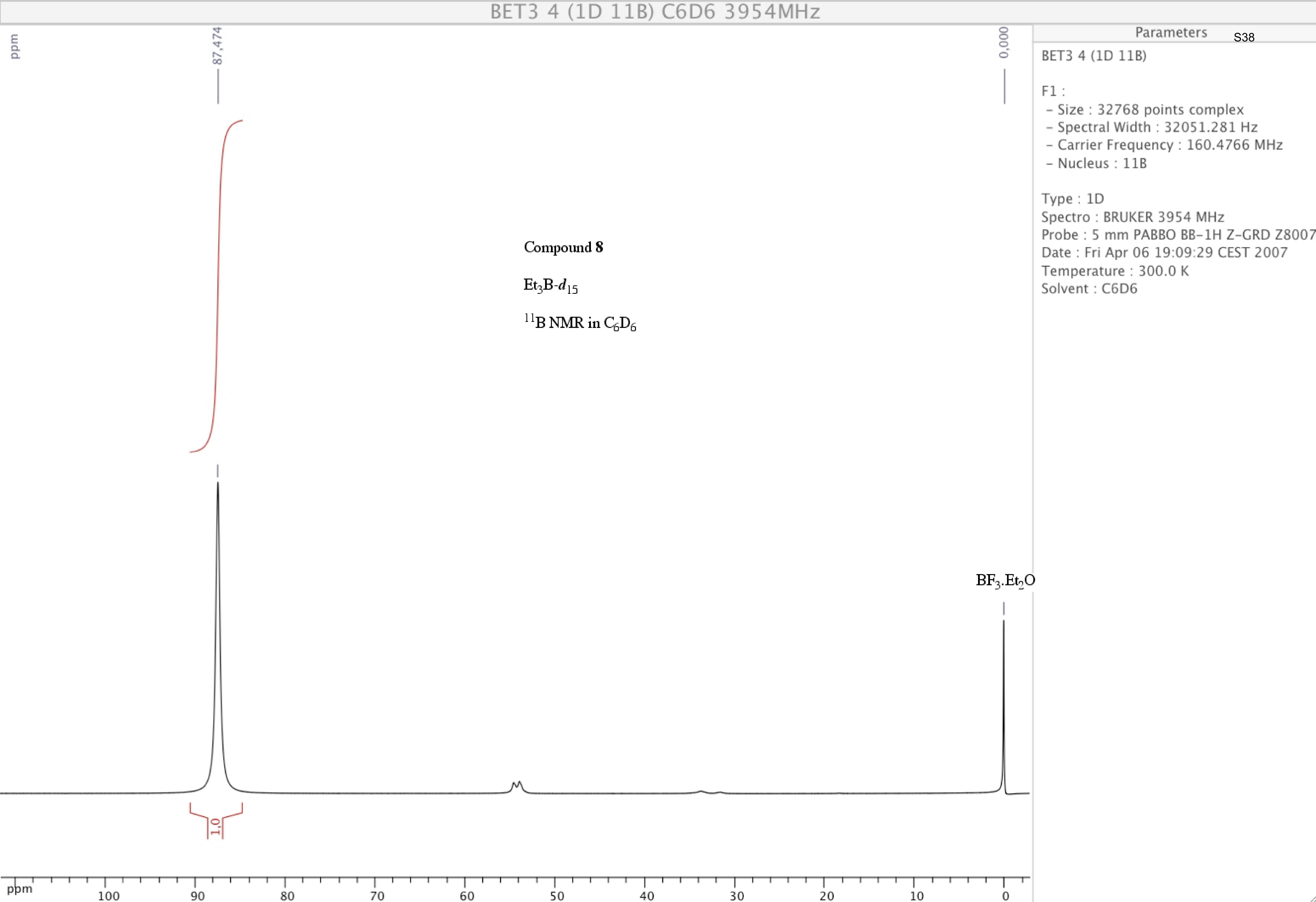
BET3 4 (1D 11B) C6D6 3954MHz
ppm
87,474
0,000
Parameters
BET3 4 (1D 11B)
F1 :
- Size : 32768 points complex
- Spectral Width : 32051.281 Hz
- Carrier Frequency : 160.4766 MHz
- Nucleus : 11B
Type : 1D
Spectro : BRUKER 3954 MHz
Probe : 5 mm PABBO BB-1H Z-GRD Z8007
Date : Fri Apr 06 19:09:29 CEST 2007
Temperature : 300.0 K
Solvent : C6D6
Compound 8
Et_3B-d_{15}
^{11}B NMR in C_6D_6
$BF_3.Et_2O$
1,0
ppm
100
90
80
70
60
50
40
30
20
10
0

B(CD2CH3)3 1 (1D 1H) CDCl3 500MHz

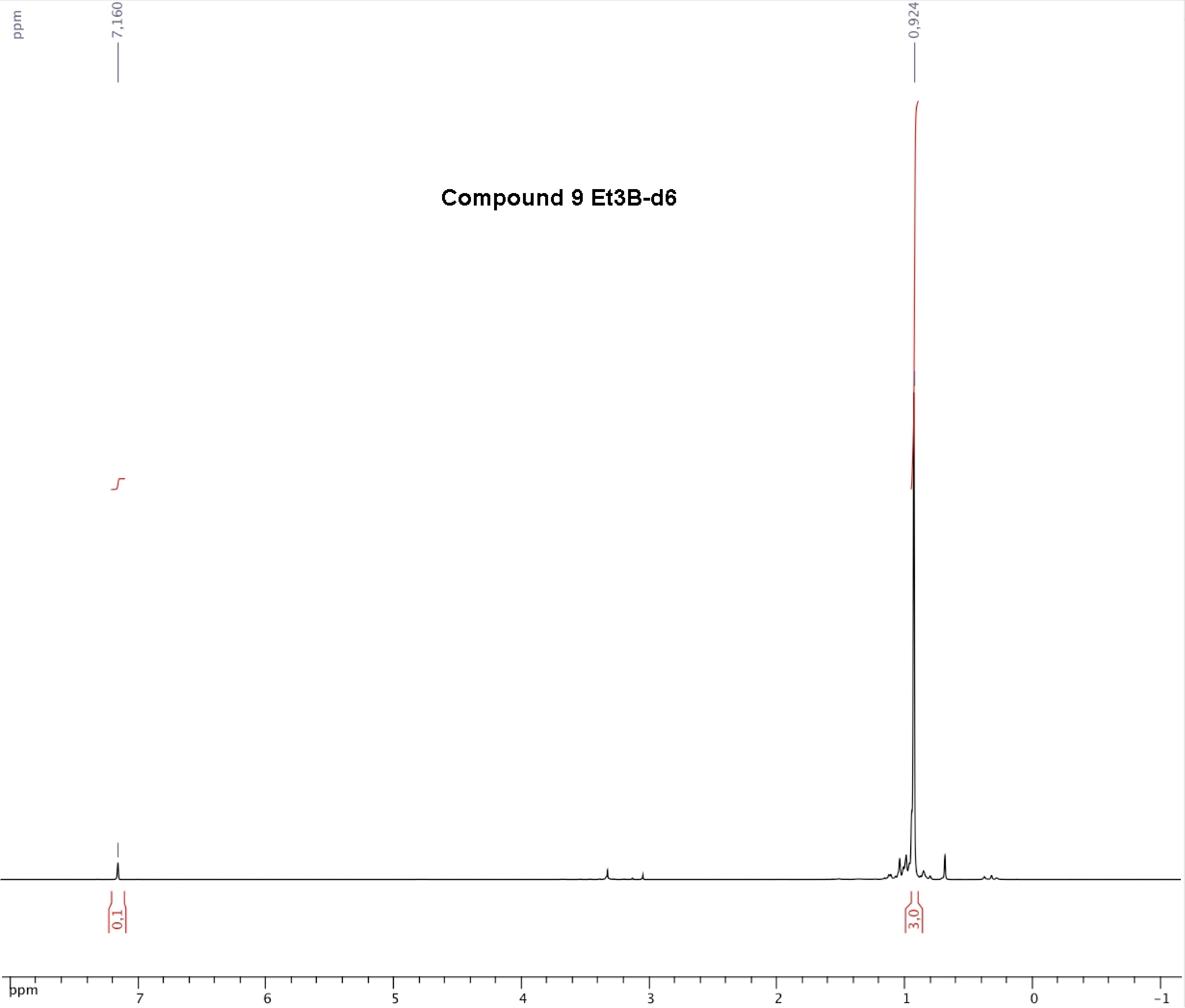

Parameters

B(CD2CH3)3 1 (1D 1H)

F1 :
- Size : 32768 points complex
- Spectral Width : 10000.0 Hz
- Carrier Frequency : 500.193 MHz
- Nucleus : 1H

Number of scans : 16

Type : 1D
Spectro : BRUKER 500 MHz
Probe : 5 mm BBI 1H-BB Z-GRD Z810701/
Date : Wed May 30 16:36:58 CEST 2007
Temperature : 298.0 K
Solvent : CDCl3

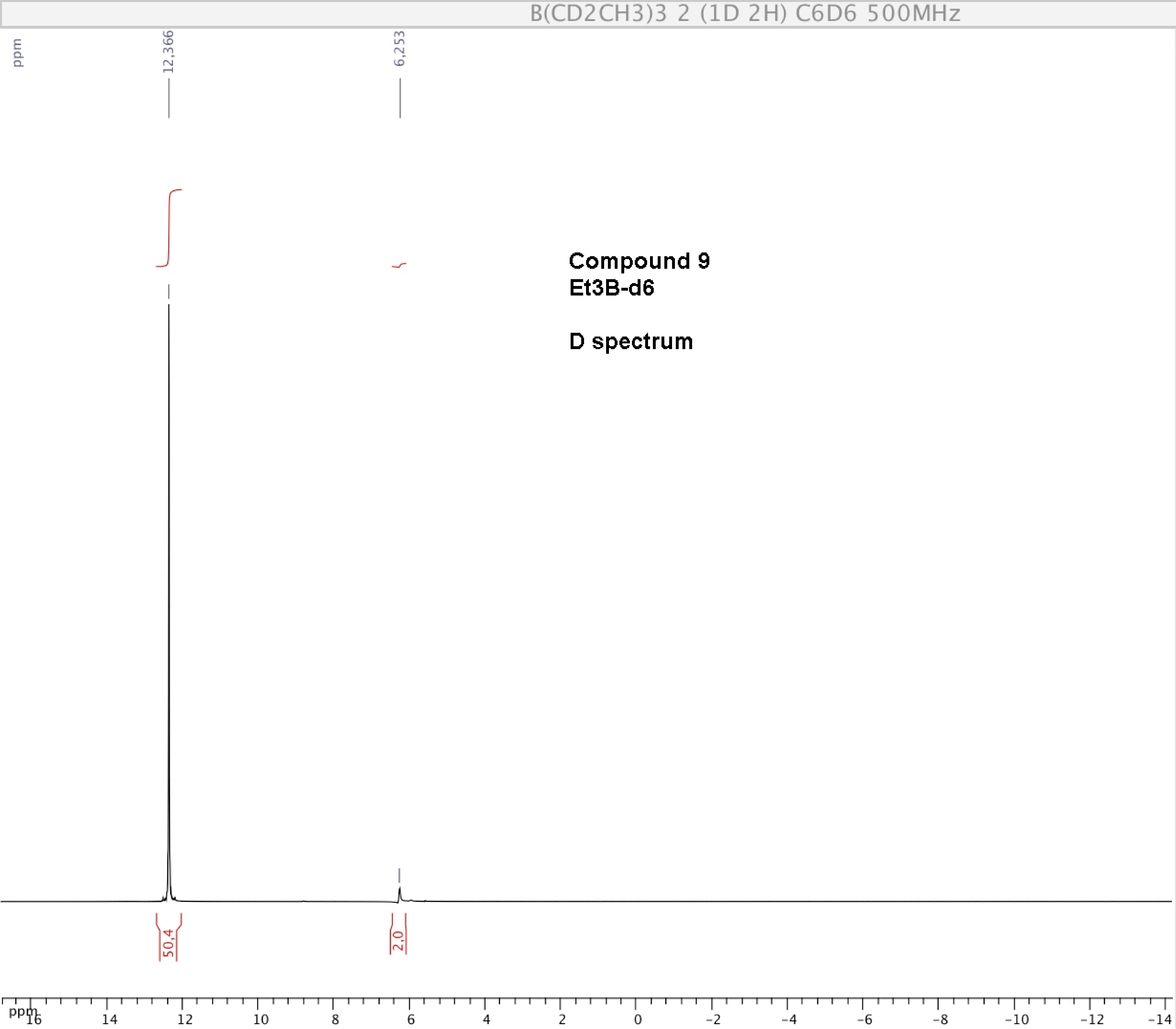

Compound 9
Et3B-d6

D spectrum

Parameters

B(CD2CH3)3 2 (1D 2H)

F1 :
- Size : 16384 points complex
- Spectral Width : 10000.0 Hz
- Carrier Frequency : 76.78219 MHz
- Nucleus : 2H

Number of scans : 16

Type : 1D
Spectro : BRUKER 500 MHz
Probe : 5 mm BBI 1H-BB Z-GRD Z810701/
Date : Wed May 30 16:45:39 CEST 2007
Temperature : 298.0 K
Solvent : C6D6

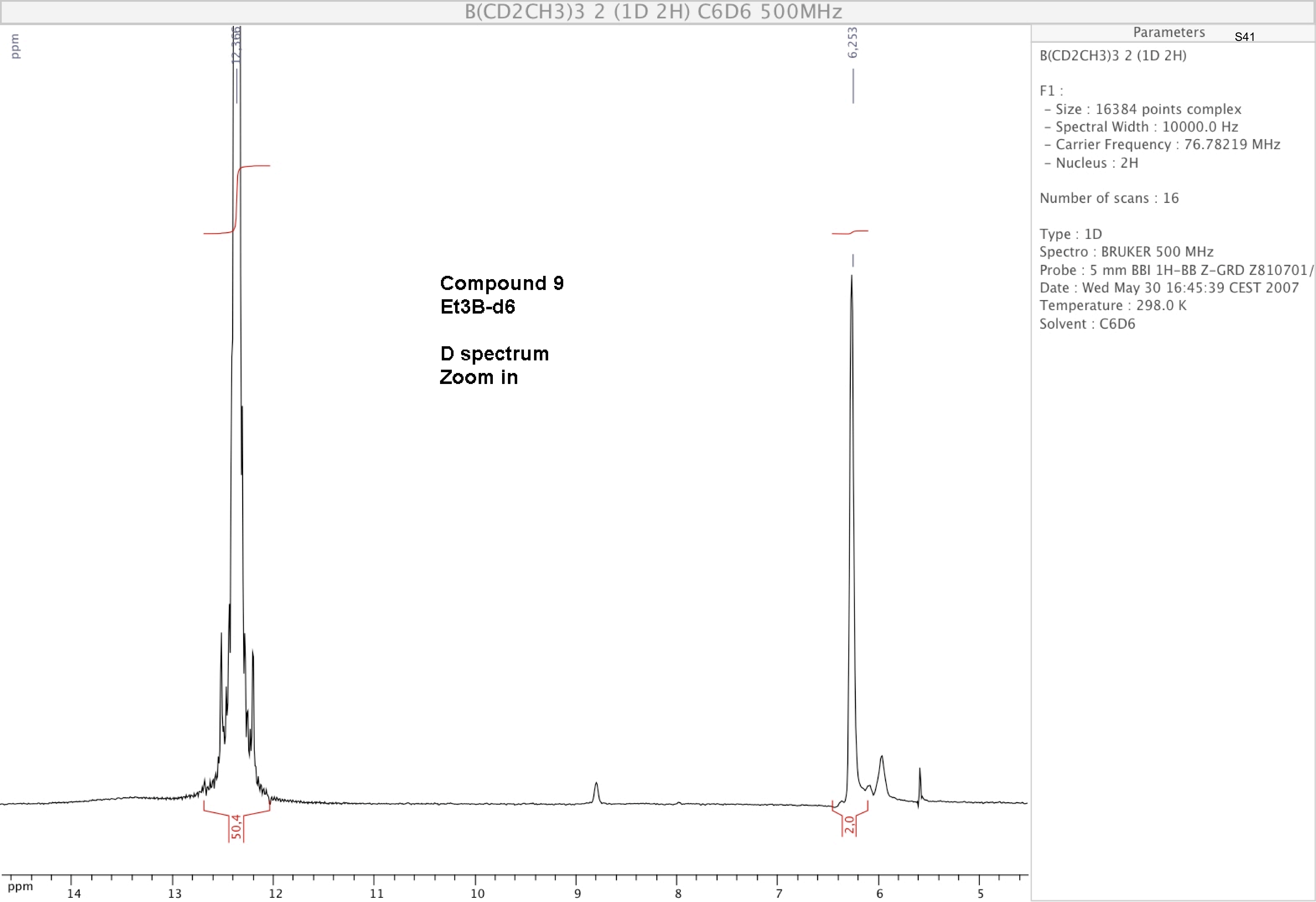
B(CD2CH3)3 2 (1D 2H) C6D6 500MHz
ppm
12,366
6,253
Compound 9
Et3B-d6
D spectrum
Zoom in
50,4
2,0
ppm
14
13
12
11
10
9
8
7
6
5
Parameters
B(CD2CH3)3 2 (1D 2H)
F1 :
- Size : 16384 points complex
- Spectral Width : 10000.0 Hz
- Carrier Frequency : 76.78219 MHz
- Nucleus : 2H
Number of scans : 16
Type : 1D
Spectro : BRUKER 500 MHz
Probe : 5 mm BBI 1H-BB Z-GRD Z810701/
Date : Wed May 30 16:45:39 CEST 2007
Temperature : 298.0 K
Solvent : C6D6

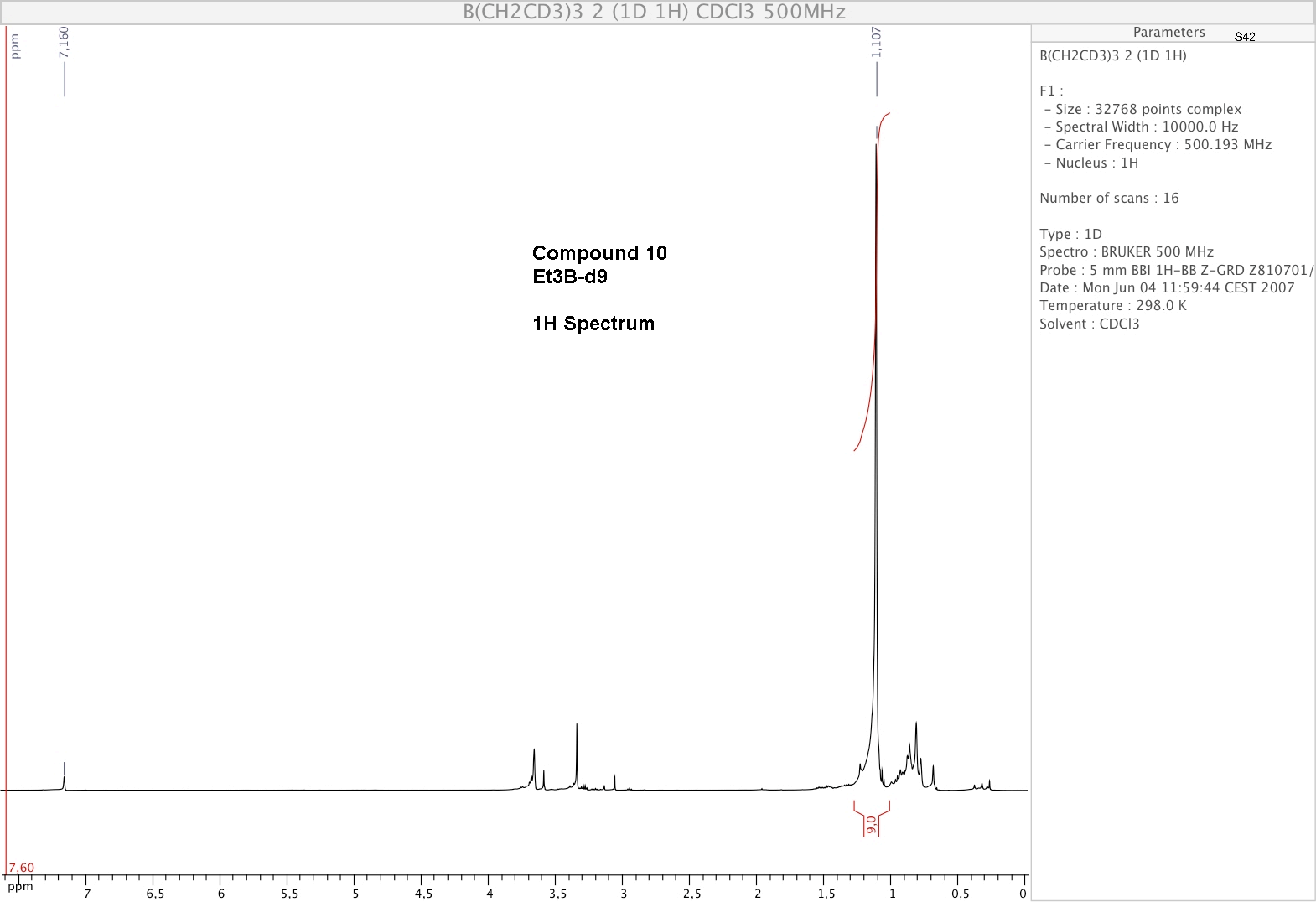
B(CH2CD3)3 2 (1D 1H) CDCl3 500MHz
7,160
1,107
Compound 10
Et3B-d9
1H Spectrum
9,0
7,60
ppm
7
6,5
6
5,5
5
4,5
4
3,5
3
2,5
2
1,5
1
0,5
0
Parameters
B(CH2CD3)3 2 (1D 1H)
F1 :
- Size : 32768 points complex
- Spectral Width : 10000.0 Hz
- Carrier Frequency : 500.193 MHz
- Nucleus : 1H
Number of scans : 16
Type : 1D
Spectro : BRUKER 500 MHz
Probe : 5 mm BBI 1H-BB Z-GRD Z810701/
Date : Mon Jun 04 11:59:44 CEST 2007
Temperature : 298.0 K
Solvent : CDCl3

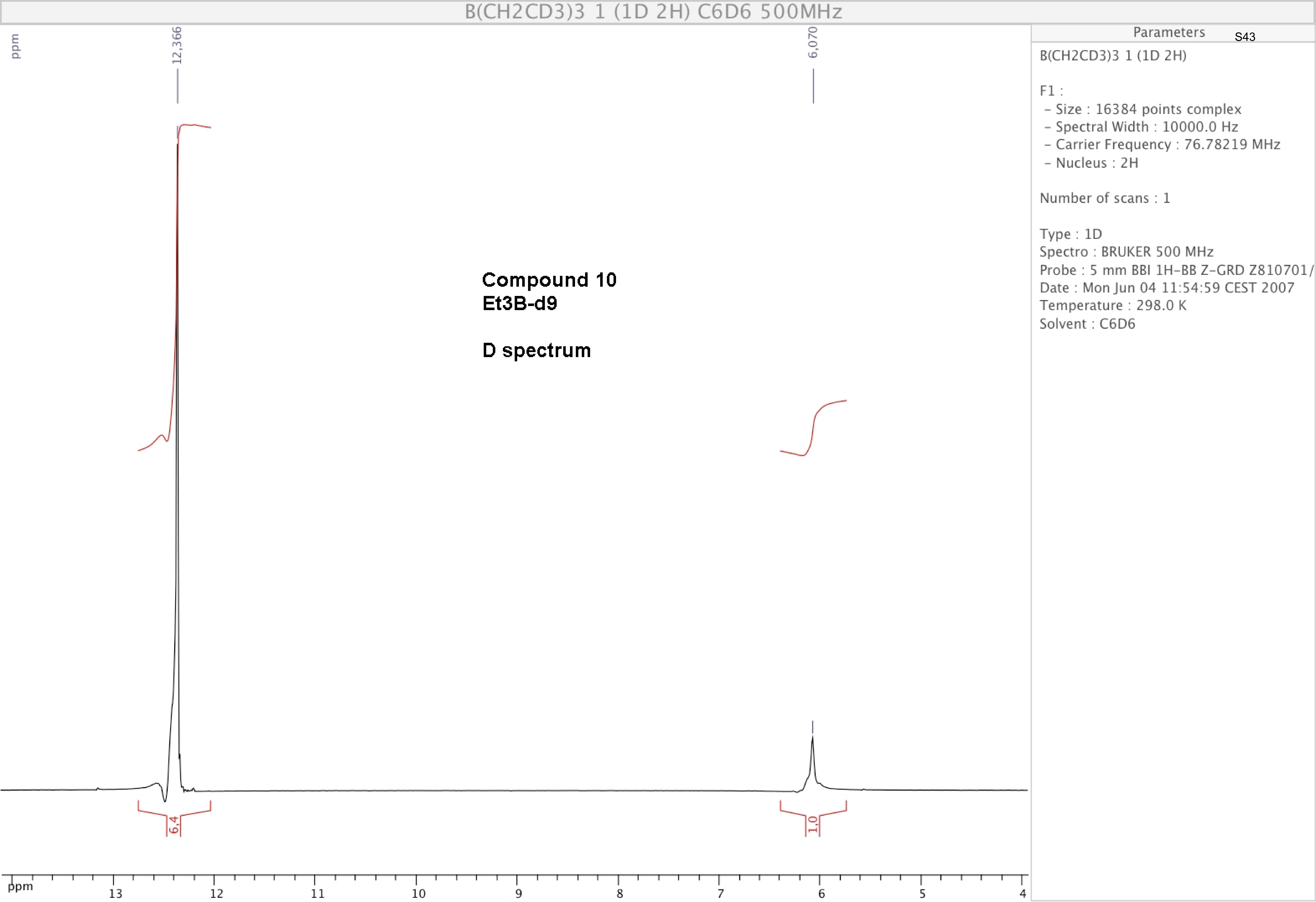
B(CH2CD3)3 1 (1D 2H) C6D6 500MHz
ppm
12,366
6,070
Compound 10
Et3B-d9
D spectrum
6,4
1,0
ppm
13
12
11
10
9
8
7
6
5
4
Parameters
B(CH2CD3)3 1 (1D 2H)
F1 :
- Size : 16384 points complex
- Spectral Width : 10000.0 Hz
- Carrier Frequency : 76.78219 MHz
- Nucleus : 2H
Number of scans : 1
Type : 1D
Spectro : BRUKER 500 MHz
Probe : 5 mm BBI 1H-BB Z-GRD Z810701/
Date : Mon Jun 04 11:54:59 CEST 2007
Temperature : 298.0 K
Solvent : C6D6
