# Supplementary material for: Part 2. Mechanistic aspects of the reduction of S-alkyl-thionocarbonates in the presence of triethylborane and air
Source: Beilstein J Org Chem. 2007 Dec 12;3:46. doi: 10.1186/1860-5397-3-46 (PMC2213662; doi:10.1186/1860-5397-3-46)

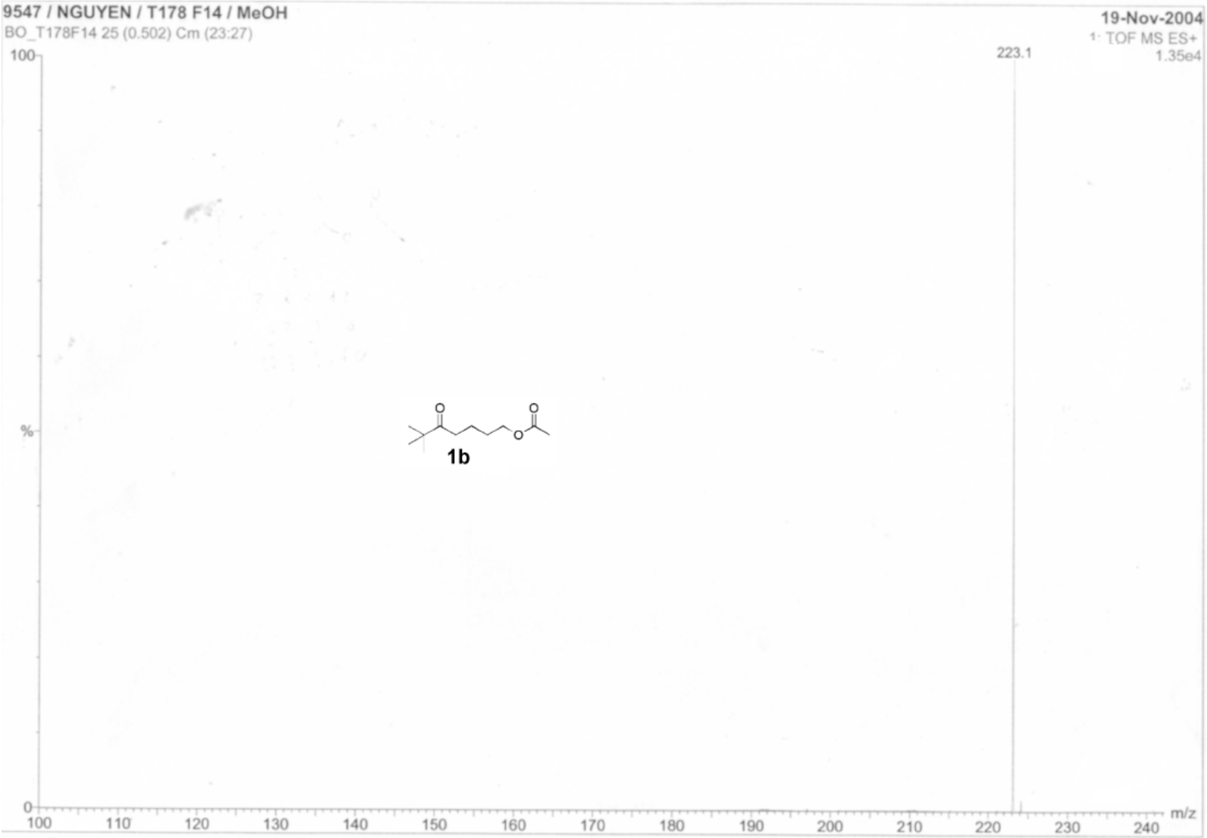
9547 / NGUYEN / T178 F14 / MeOH
BO_T178F14 25 (0.502) Cm (23:27)
19-Nov-2004
1: TOF MS ES+
1.35e4
223.1
100
%
0
1b
100 110 120 130 140 150 160 170 180 190 200 210 220 230 240
m/z

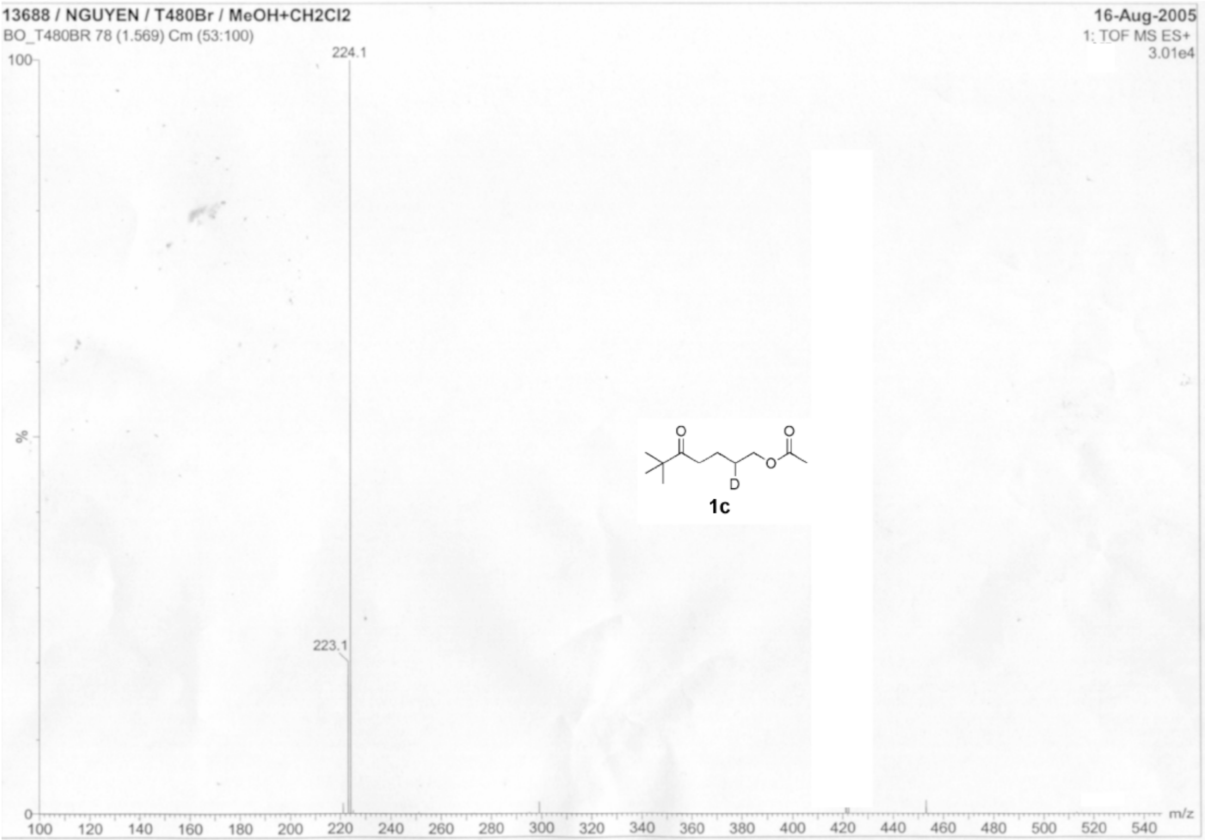
13688 / NGUYEN / T480Br / MeOH+CH2Cl2
BO_T480BR 78 (1.569) Cm (53:100)
16-Aug-2005
1: TOF MS ES+
3.01e4
224.1
223.1
100
%
0
D
1c
100 120 140 160 180 200 220 240 260 280 300 320 340 360 380 400 420 440 460 480 500 520 540
m/z

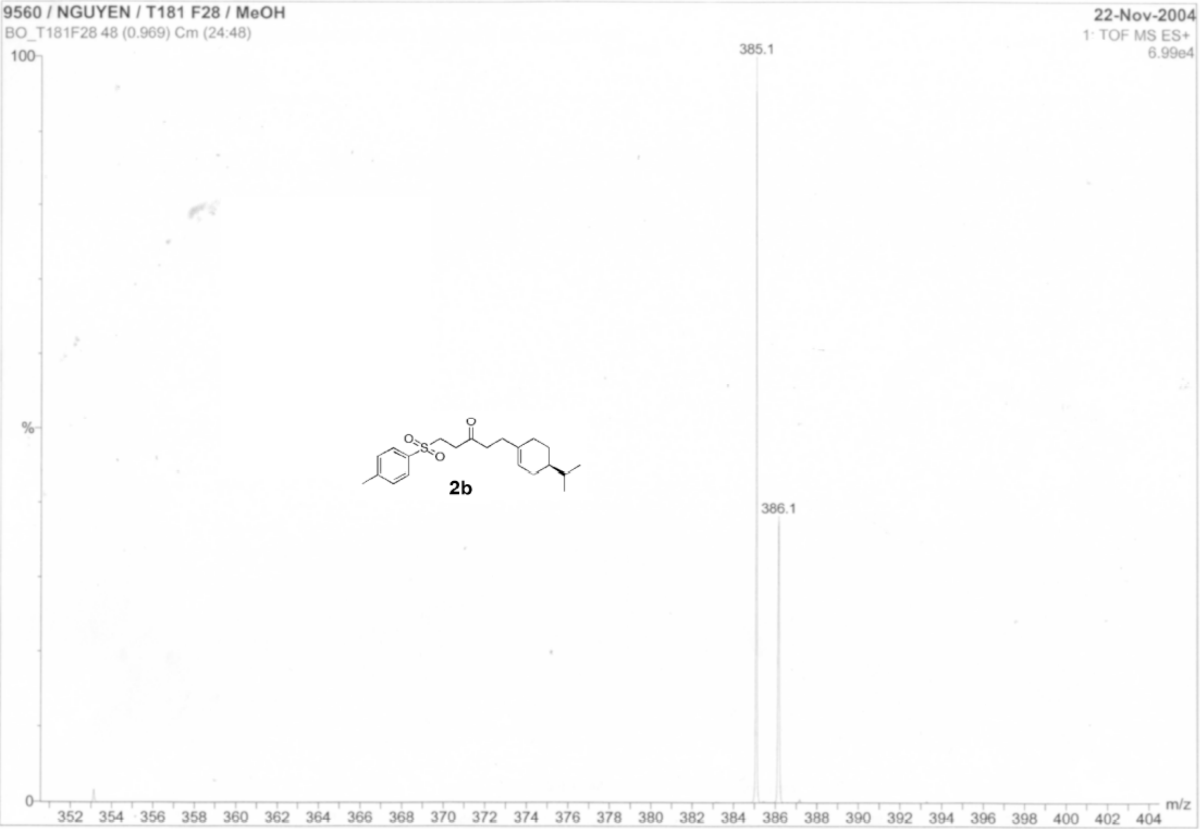
9560 / NGUYEN / T181 F28 / MeOH
22-Nov-2004
BO_T181F28 48 (0.969) Cm (24:48)
1: TOF MS ES+
6.99e4
100
%
0
385.1
386.1
2b
352 354 356 358 360 362 364 366 368 370 372 374 376 378 380 382 384 386 388 390 392 394 396 398 400 402 404
m/z

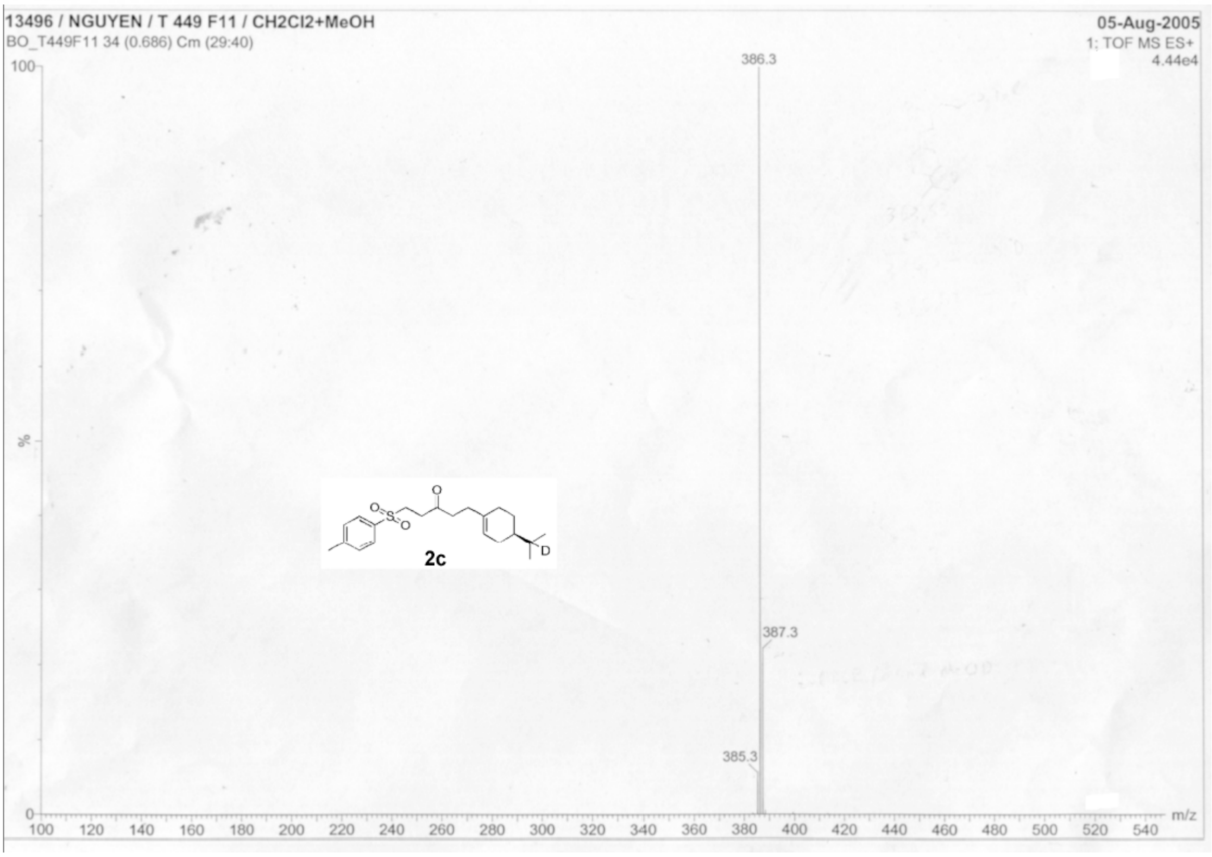
13496 / NGUYEN / T 449 F11 / CH2Cl2+MeOH
05-Aug-2005
BO_T449F11 34 (0.686) Cm (29:40)
1: TOF MS ES+
4.44e4
100
%
0
386.3
387.3
385.3
2c
D
100 120 140 160 180 200 220 240 260 280 300 320 340 360 380 400 420 440 460 480 500 520 540
m/z

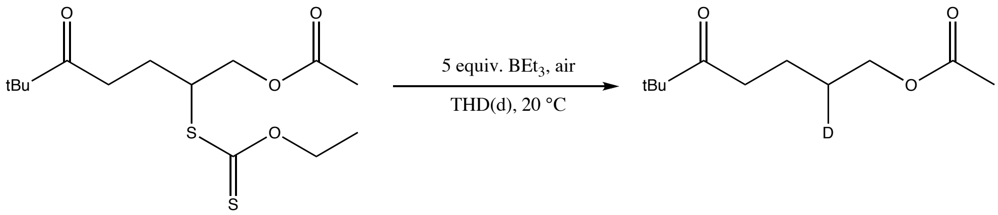

Line#:1 R.Time:6.000(Scan#:421)
MassPeaks:80
RawMode:Single 6.000(421) BasePeak:57.15(8390970)
BG Mode:None

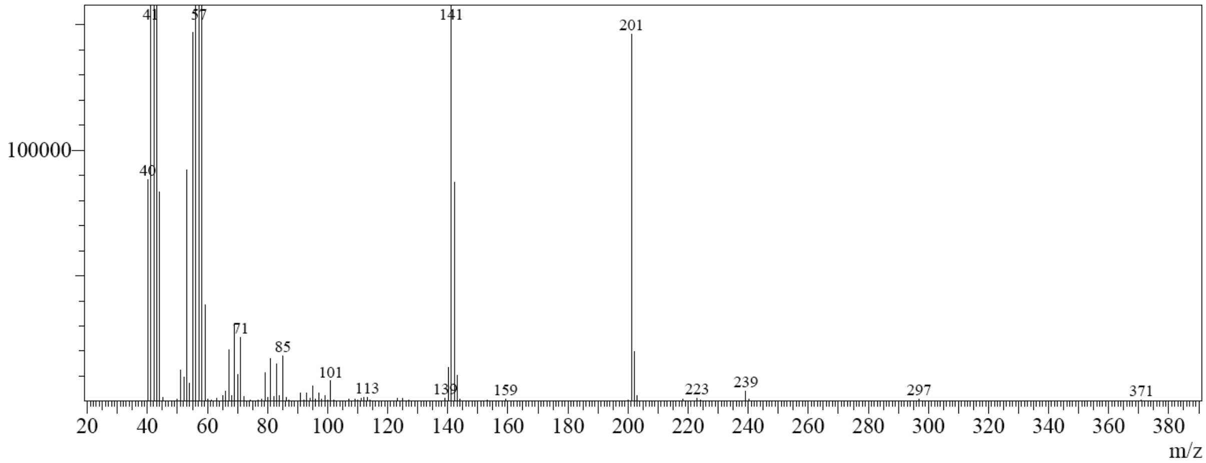

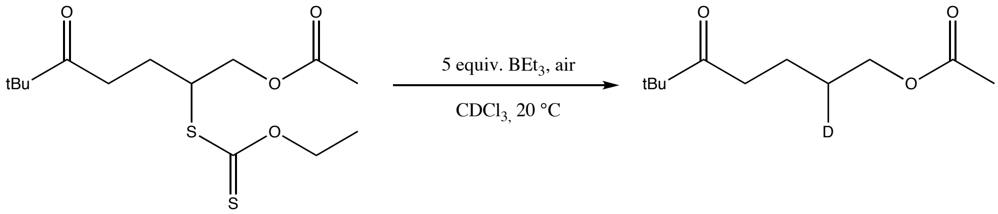

Line#:1 R.Time:5.992(Scan#:420)
MassPeaks:130
RawMode:Averaged 5.933-6.050(413-427) BasePeak:57.05(8418437)
BG Mode:None

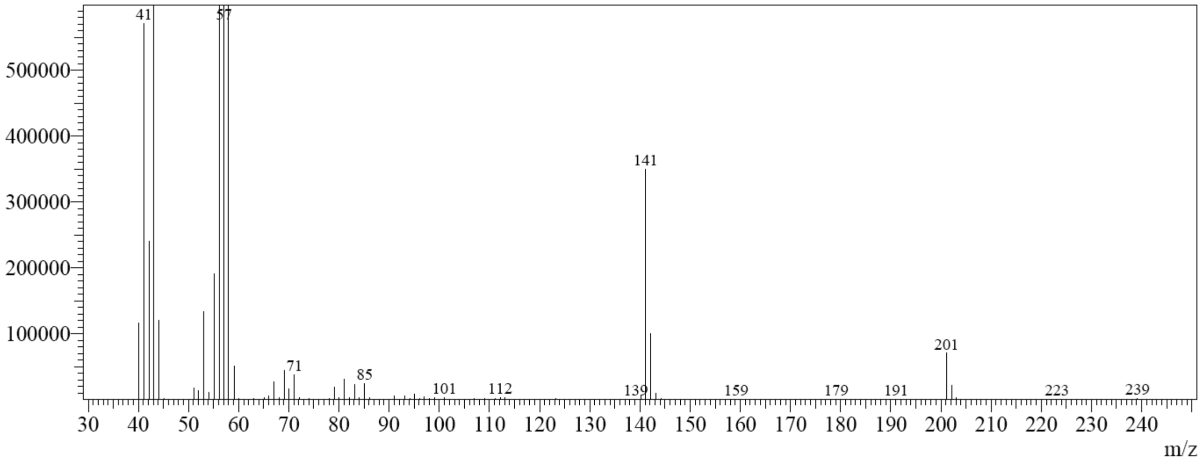

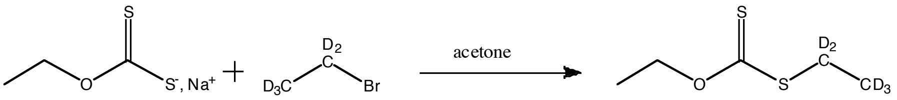

Line#:1 R.Time:5.225(Scan#:328)
MassPeaks:108
RawMode:Averaged 5.167-5.283(321-335) BasePeak:57.05(8428816)
BG Mode:None

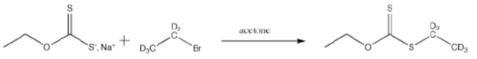

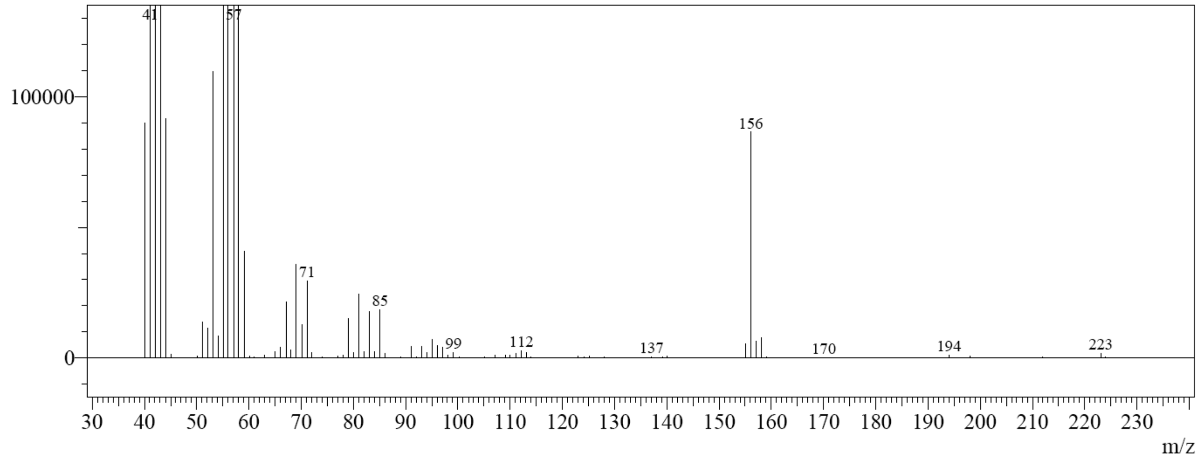

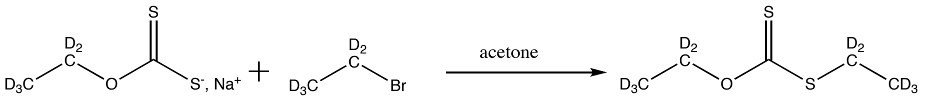

Line#:1 R.Time:5.158(Scan#:320)
MassPeaks:118
RawMode:Averaged 5.117-5.208(315-326) BasePeak:57.05(8430370)
BG Mode:None

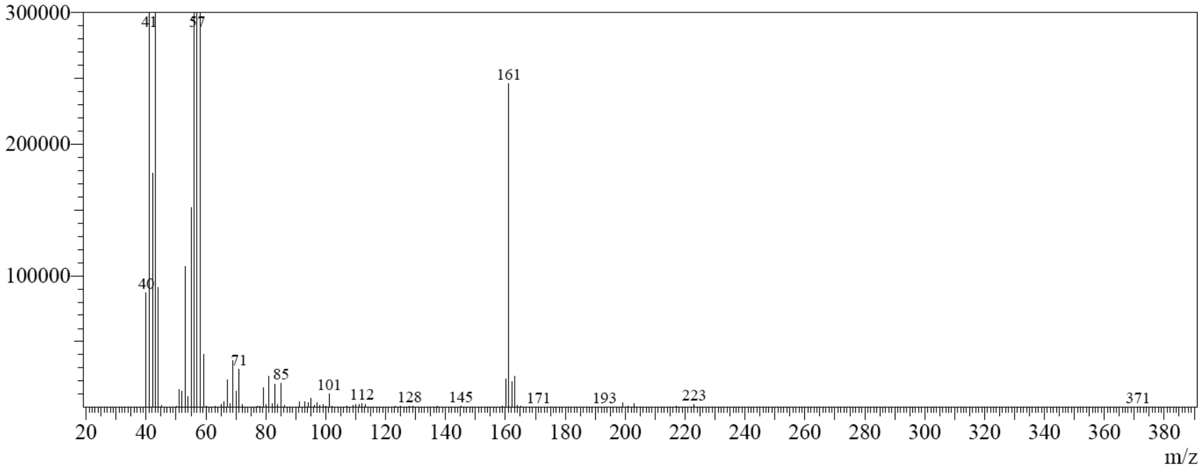

Compound **3**

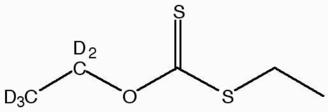

Chemical Formula: $C_5H_5D_5OS_2$
Exact Mass: 155,04869
Molecular Weight: 155,29311

Line#:1 R.Time:3.233(Scan#:89)
MassPeaks:120
RawMode:Averaged 3.217-3.283(87-95) BasePeak:57.15(8405324)
BG Mode:None

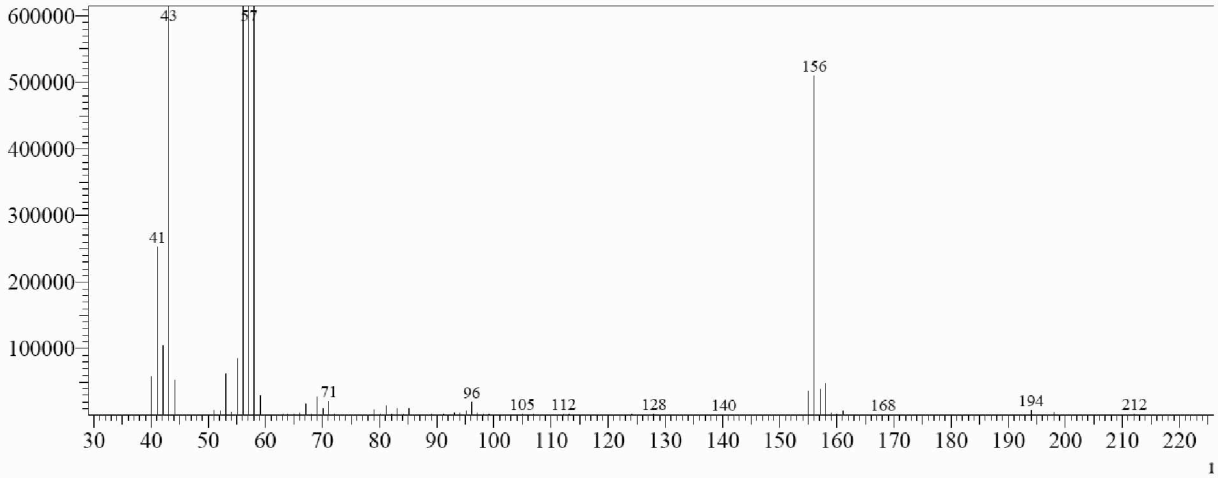

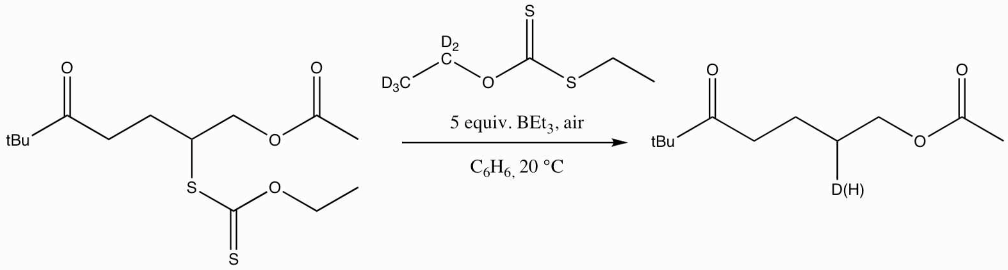

Line#:1 R.Time:6.000(Scan#:421)
MassPeaks:94
RawMode:Averaged 5.950-6.008(415-422) BasePeak:57.05(8396871)
BG Mode:None

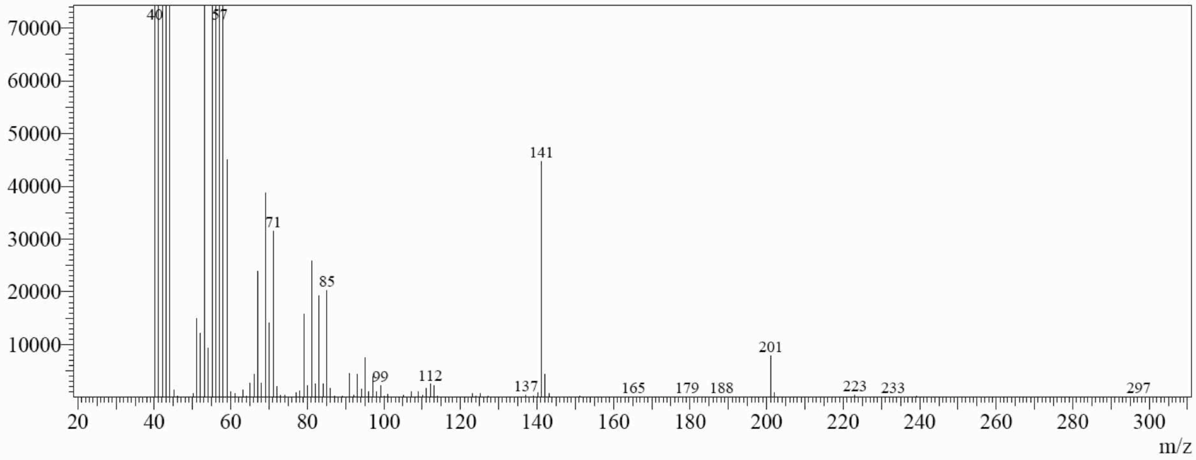

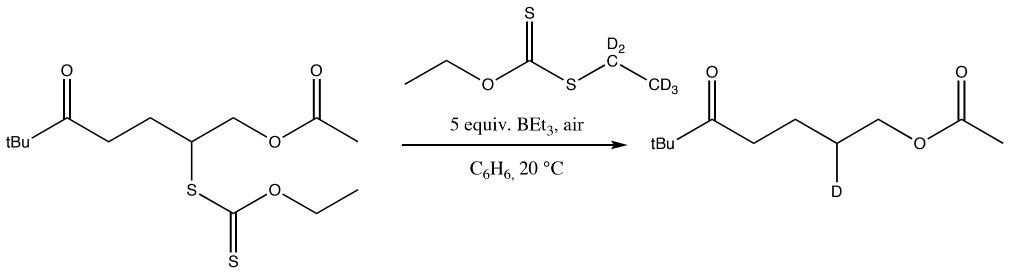

Line#:1 R.Time:5.992(Scan#:420)
MassPeaks:111
RawMode:Averaged 5.958-6.008(416-422) BasePeak:57.05(8396884)
BG Mode:None

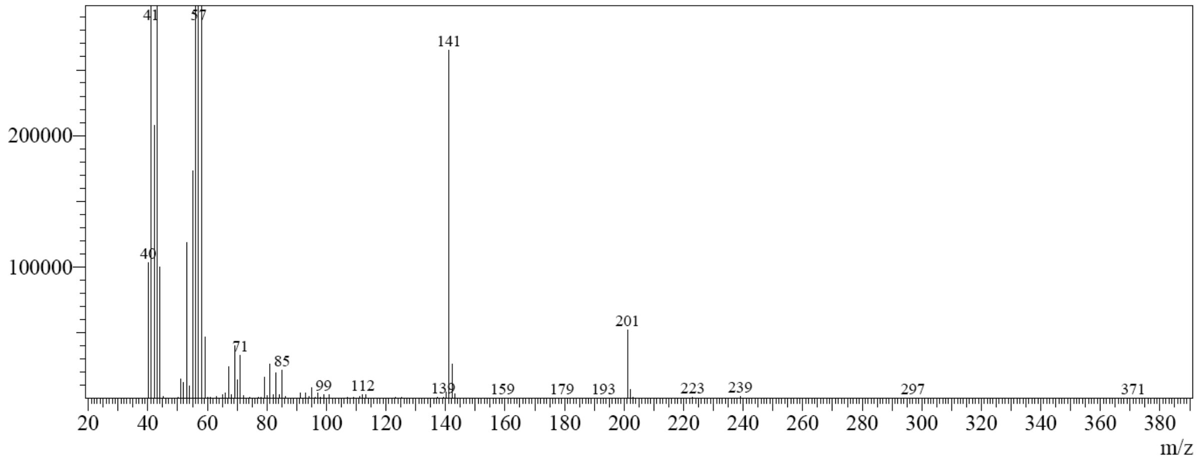

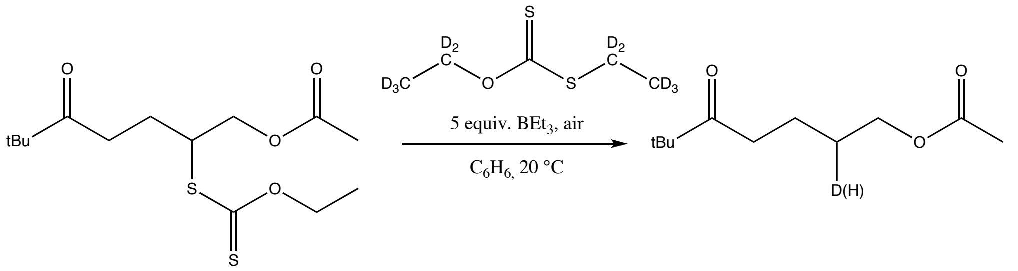

Line#:1 R.Time:5.992(Scan#:420)
MassPeaks:95
RawMode:Averaged 5.975-6.008(418-422) BasePeak:57.05(8395550)
BG Mode:None

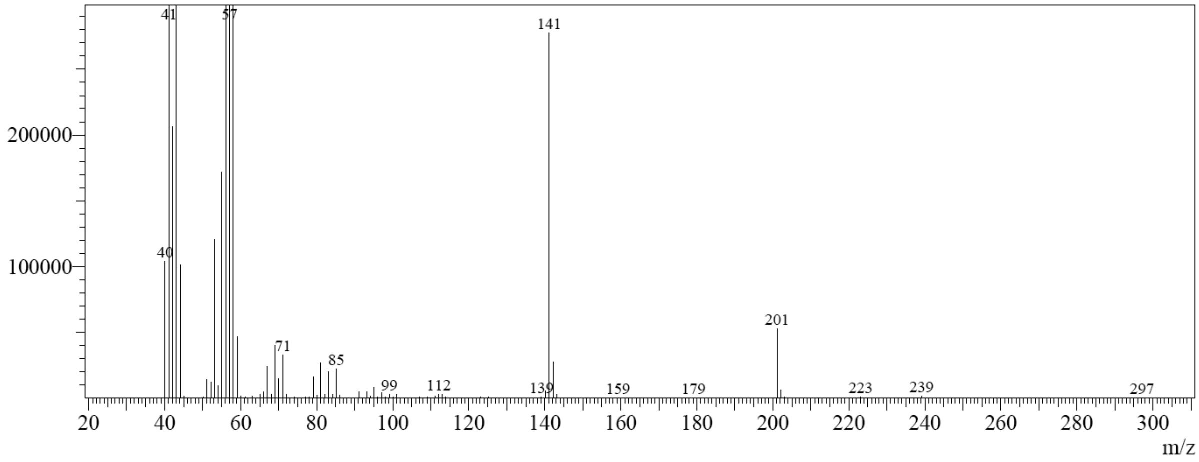

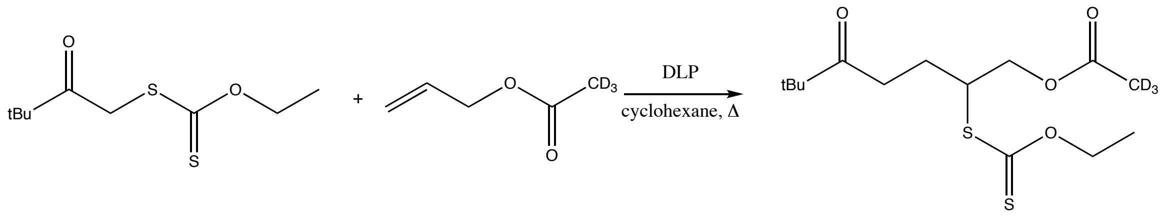

Line#:1 R.Time:15.483(Scan#:1559)
MassPeaks:108
RawMode:Single 15.483(1559) BasePeak:57.05(8431497)
BG Mode:None

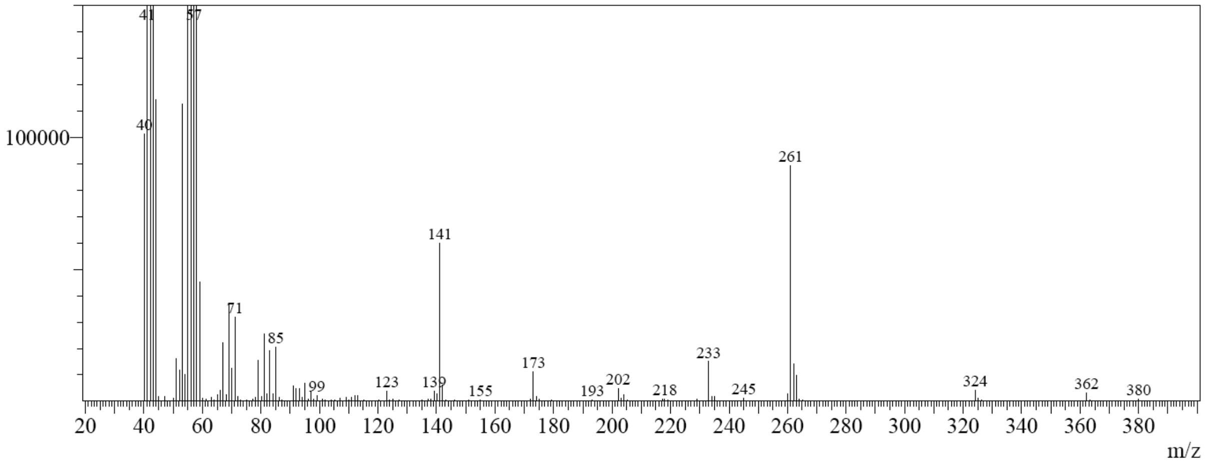

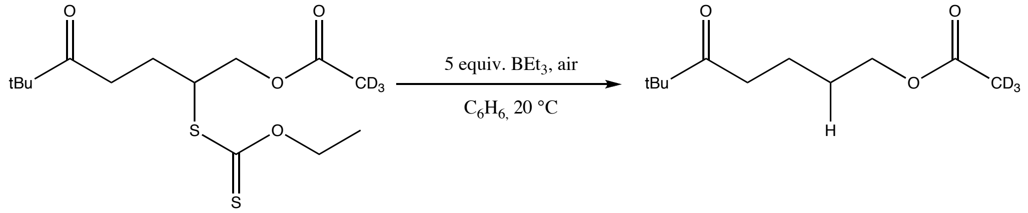

Line#:1 R.Time:5.975(Scan#:418)
MassPeaks:117
RawMode:Averaged 5.942-6.017(414-423) BasePeak:57.05(8398090)
BG Mode:None

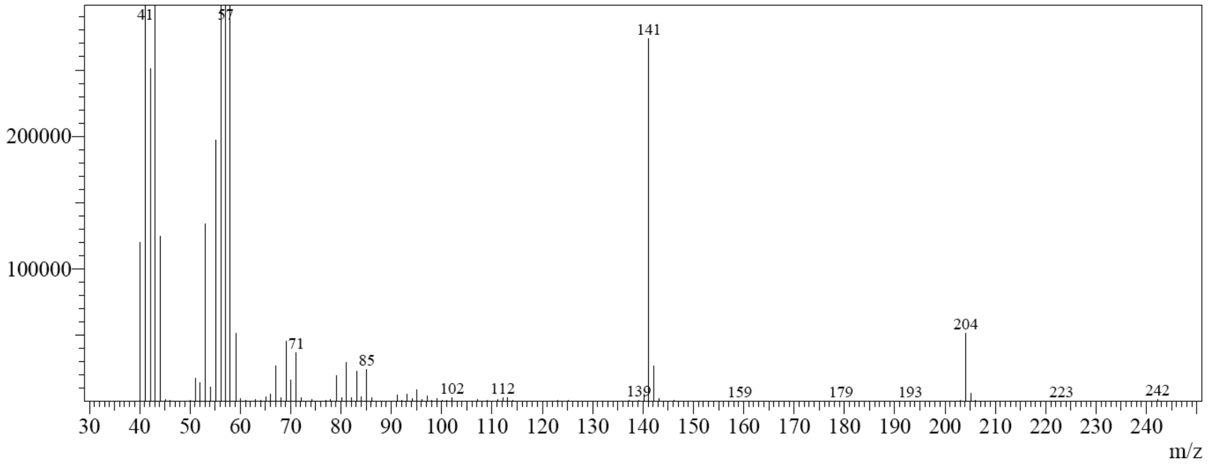

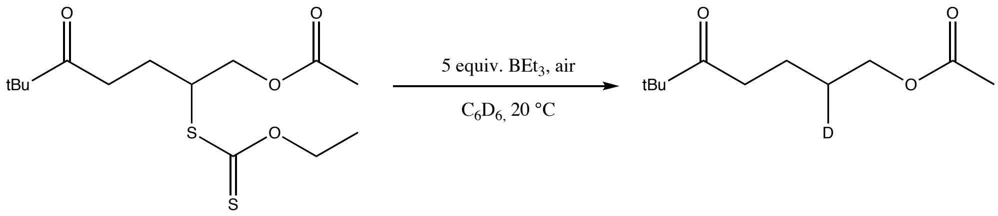

Line#:1 R.Time:5.958(Scan#:416)
MassPeaks:86
RawMode:Single 5.958(416) BasePeak:57.15(8389521)
BG Mode:None

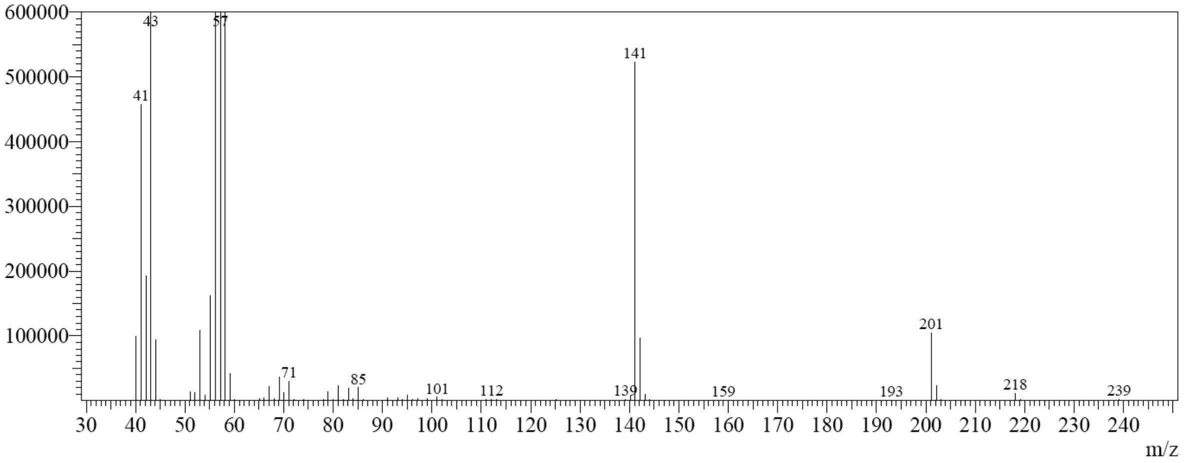

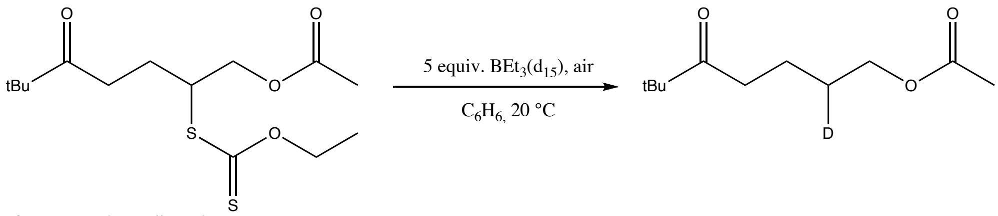

Line#:1 R.Time:5.942(Scan#:414)
MassPeaks:85
RawMode:Single 5.942(414) BasePeak:57.15(8396984)
BG Mode:None

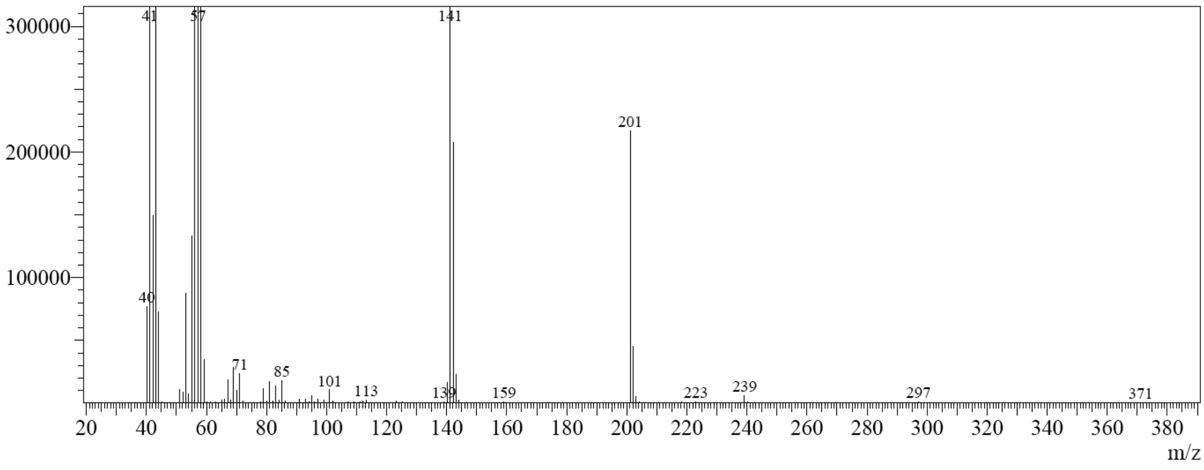

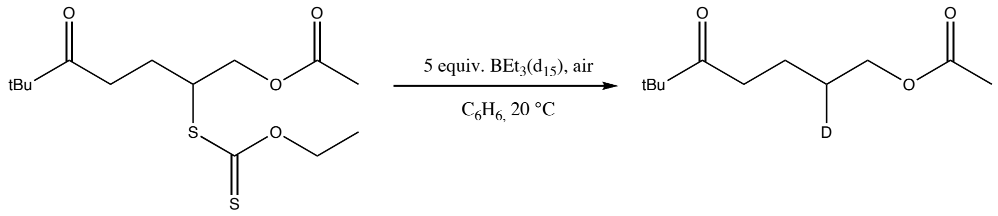

Teflon RBF

Line#:1 R.Time:5.983(Scan#:419)
MassPeaks:101
RawMode:Averaged 5.967-5.992(417-420) BasePeak:57.05(8410628)
BG Mode:None

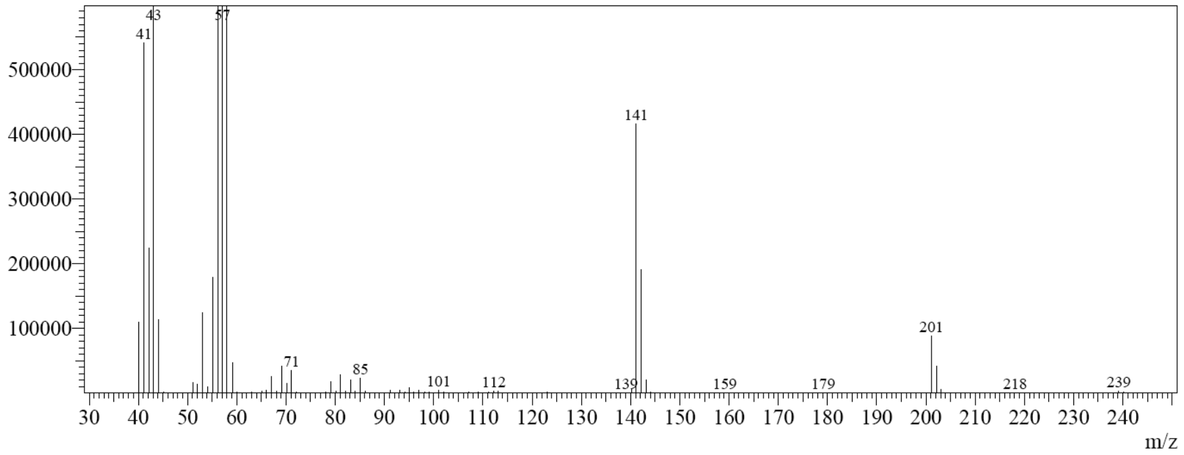

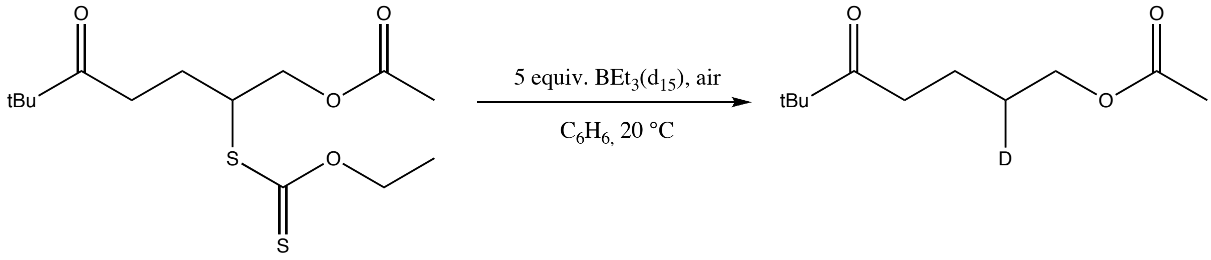

Teflon RBF + glass rings

Line#:1 R.Time:5.965(Scan#:416)
MassPeaks:85
RawMode:Single 5.965(416) BasePeak:57.14(8389514)
BG Mode:None

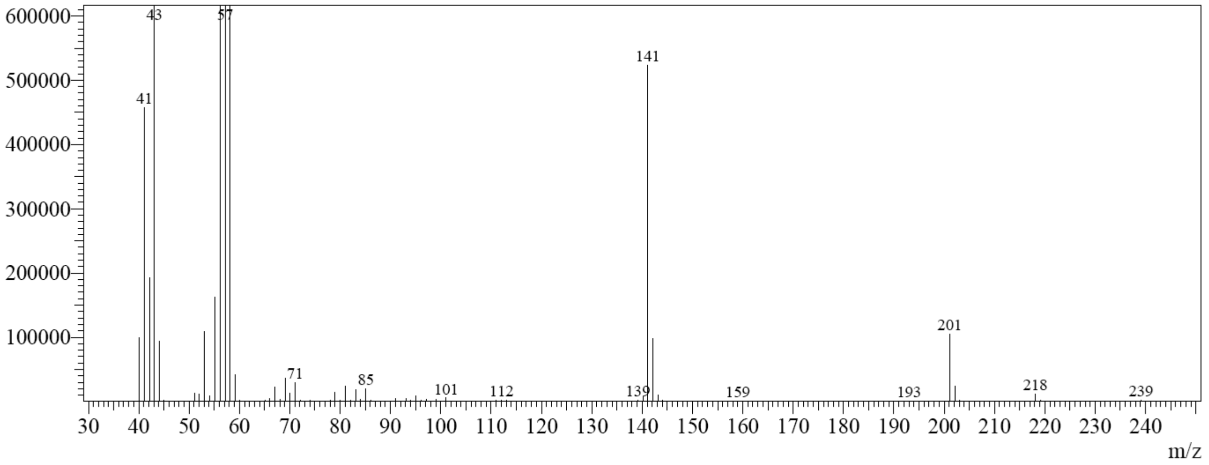

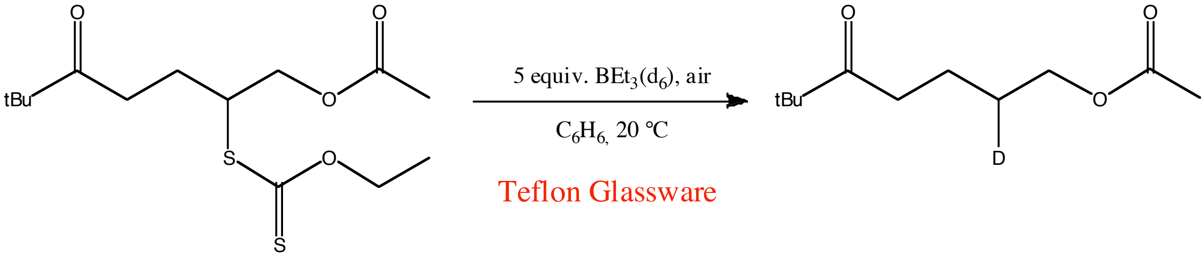

Line#:1 R.Time:5.958(Scan#:416)
MassPeaks:65
RawMode:Single 5.958(416) BasePeak:57.05(8428925)
BG Mode:None

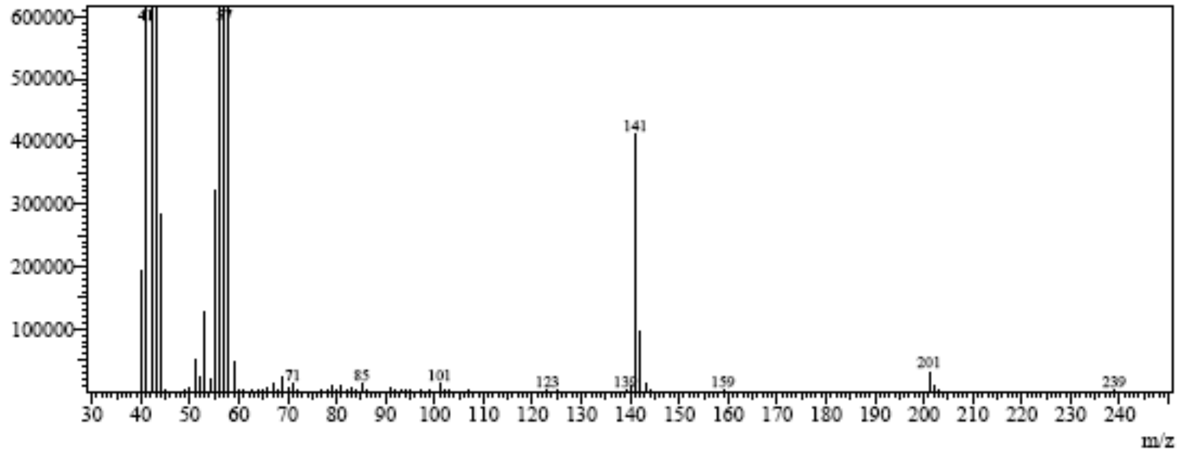

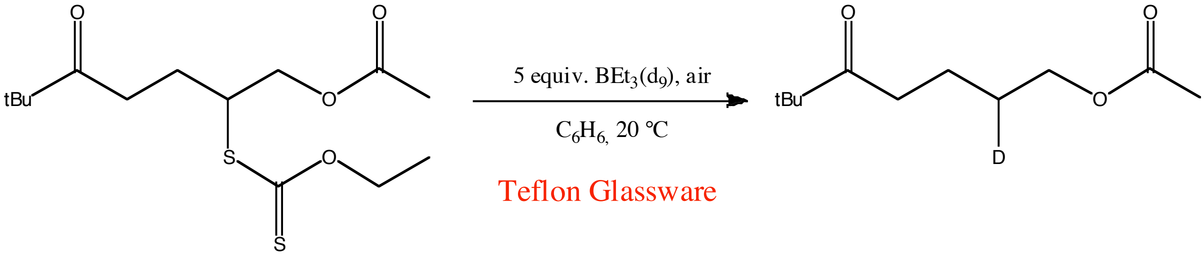

Line#:1 R.Time:5.967(Scan#:417)
MassPeaks:52
RawMode:Single 5.967(417) BasePeak:57.15(8388842)
BG Mode:None

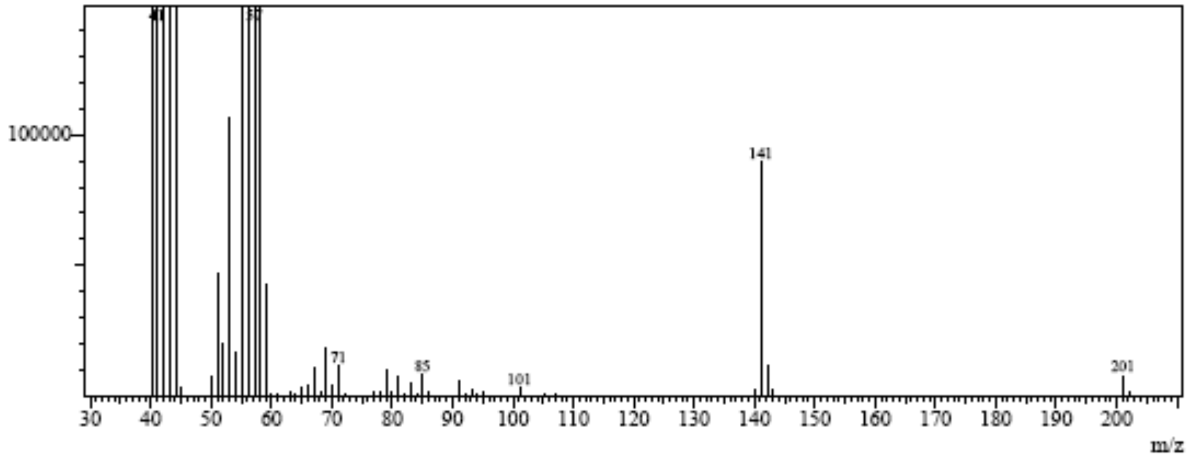

Deuterium incorporation measurements

Table	Entry	m/z 141 Integration	m/z 142 Integration	m/z 142 corrected for natural abundance	% Deuteration based on peaks 141/142	m/z 201 Integration	m/z 202 Integration	m/z 202 corrected for natural abundance	% Deuteration based on peaks 201/202	% Deuteration Average Value
1	14 [g]	350392	100713	35039,2	15,78	70477	22312	9162,01	15,72	**15,75**

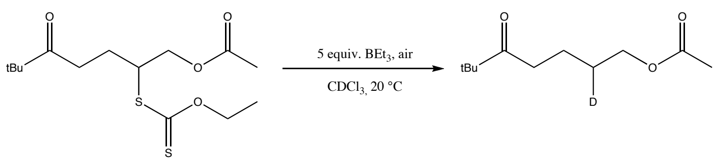

1	19	816661	87221	81666,1	0,68	146383	19694	19029,79	0,45	**0,56**

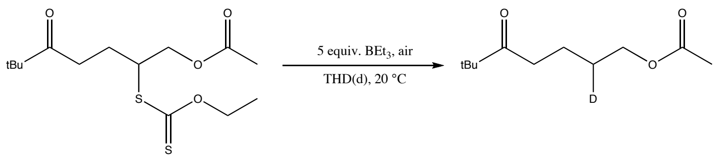

2	1	44780	4318	4478	-0,36	7795	943	1013,35	-0,91	**-0,63**

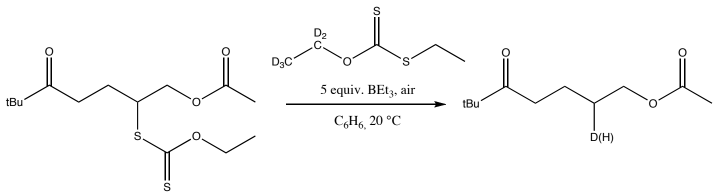

2	2	264669	25852	26466,9	-0,23	51900	6637	6747	-0,21	**-0,22**

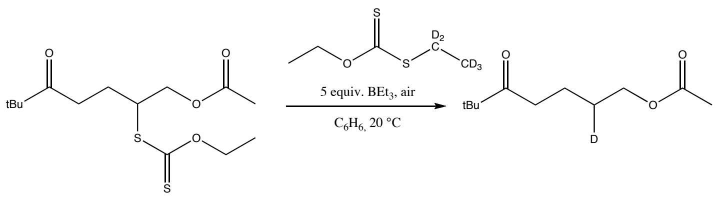

2	3	277333	27343	27733,3	-0,14	53010	6367	6891,3	-1,00	**-0,57**

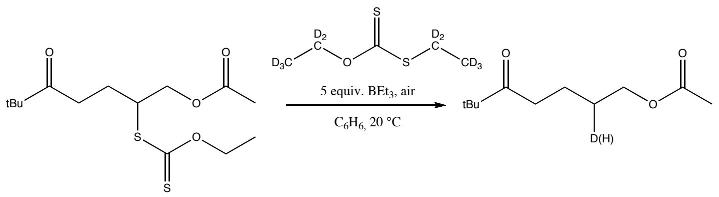

3	1	523335	97405	52333,5	7,93	104388	23466	13570,44	8,66	**8,29**

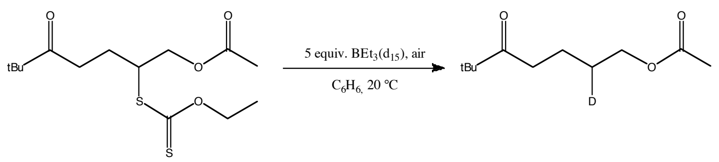

Deuterium incorporation measurements

Table	Entry	m/z 141 Integration	m/z 142 Integration	m/z 142 corrected for natural abundance	% Deuteration based on peaks 141/142	m/z 201 Integration	m/z 202 Integration	m/z 202 corrected for natural abundance	% Deuteration based on peaks 201/202	% Deuteration Average Value
3	2		50254	26881,2	8,00	68919	15497	8959,47	8,66	**8,33**
3	3	545903	255493	54590,3	26,90	108257	53232	14073,41	26,56	**26,73**
3	4	416641	190454	41664,1	26,31	87873	41393	11423,49	25,43	**25,87**
3	5	410239	94345	41023,9	11,50	31783	9420	4131,79	14,27	**12,88**
3	6	89743	11724	8974,3	2,97	7591	1303	986,83	4,00	**3,49**
3	7	523335	97405	52333,5	7,93	104388	23466	13570,44	8,66	**8,29**

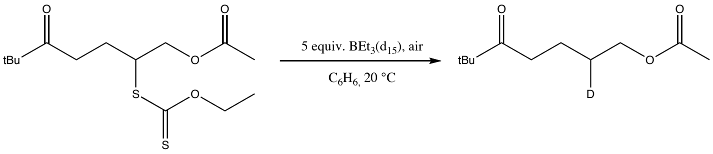

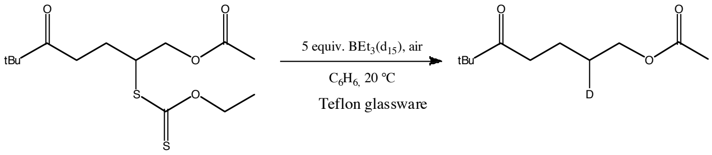

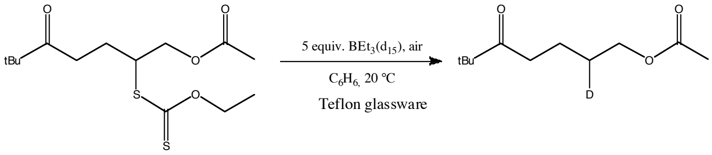

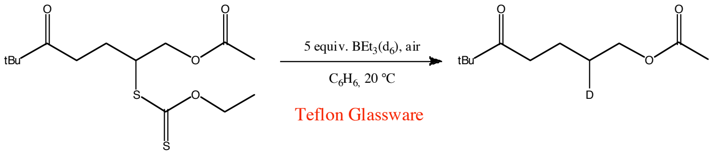

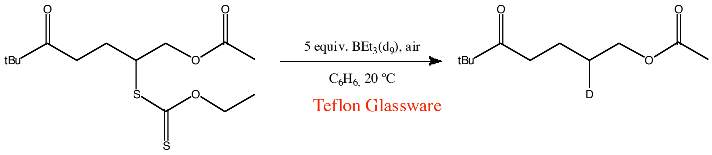

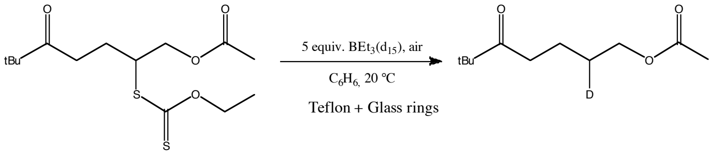

Deuterium incorporation measurements

Table	Entry	m/z 141 Integration	m/z 142 Integration	m/z 142 corrected for natural abundance	% Deuteration based on peaks 141/142	m/z 201 Integration	m/z 202 Integration	m/z 202 corrected for natural abundance	% Deuteration based on peaks 201/202	% Deuteration Average Value
Chart 2		59842	6070	5984,2	0,14	2392	337	310,96	1,08	**0,61**

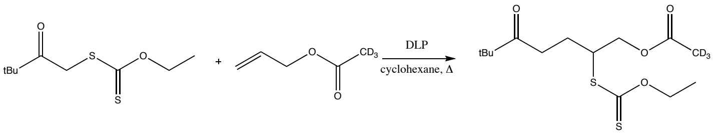

Chart 2		273903	27054	27390,3	-0,12	51747	6385	6727,11	-0,67	**-0,39**

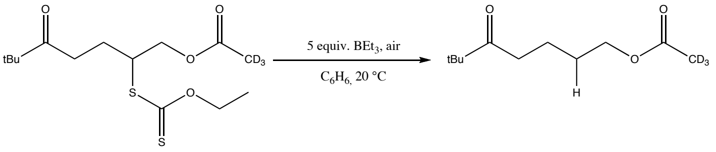

Supplement: File 4 — Part 2. Mechanistic aspects of the reduction of S-alkyl-thionocarbonates in the presence of triethylborane and air. Detailed procedures for preparation of new compounds and their spectroscopic data, pp 44–56. [file Beilstein_J_Org_Chem-03-46-s004.pdf]
